# Correction: Individual differences in associative/semantic priming: Spreading of activation in semantic memory and epistemically unwarranted beliefs

**DOI:** 10.1371/journal.pone.0330531

**Published:** 2025-08-18

**Authors:** The PLOS One Staff

## Notice of Republication

This article was republished on August 5, 2025, to correct errors in the affiliations, missing reference links, and various in-text typos.

The publisher apologizes for the errors. Please download this article again to view the correct version. The originally published, uncorrected article and the republished, corrected articles are provided here for reference.

## Supporting information

S1 File Originally published, uncorrected article. (S1_File.PDF)

S2 File Republished, corrected article. (S2_File.PDF)
