# Supplementary material for: Individual differences in associative/semantic priming: Spreading of activation in semantic memory and epistemically unwarranted beliefs
Source: PLoS One. 2025 Feb 11;20(2):e0313239. doi: 10.1371/journal.pone.0313239 (PMC11813106; doi:10.1371/journal.pone.0313239)
Supplement: S1 File — More detailed account of our analytical and technical decisions (along with relevant code). (PDF) [file pone.0313239.s001.pdf]

Individual differences in associative/semantic priming: Spreading of activation in semantic memory and epistemically unwarranted beliefs – SUPPLEMENTARY MATERIAL: Data analysis details (English version)

Daniel Huete-Pérez[1], Robert Davies[2], Javier Rodríguez-Ferreiro[3], and Pilar Ferré[1]

[1] Universitat Rovira i Virgili, Department of Psychology, Research Center for Behavior Assessment (CRAMC), Tarragona, Spain.

[2] Department of Psychology, Lancaster University, Bailrigg, Lancaster, United Kingdom.

[3] Grup de Recerca en Cognició i Llenguatge (GRECIL), Departament de Cognició, Desenvolupament i Psicologia de la Educació, Secció de Processos Cognitius, Institut de Neurociències (INUB), Universitat de Barcelona (UB), Barcelona, Spain.

Abstract: Starting from the *enhanced spreading of activation through semantic memory* (one of the explanatory mechanisms attempting to explain some manifestations observed in schizophrenia) and the *psychosis continuum* (a dimensional approach to psychotic disorders, where ‘normality’ and ‘psychopathology’ are not qualitatively different in nature but placed on varying levels of the same continuum), the main aim of the present research was to explore whether there are individual differences in associative/semantic priming in people with different levels of epistemically unwarranted beliefs (EUB). Participants varying in paranormal, pseudoscientific and conspiracy endorsement completed a primed lexical decision task containing related prime-target words (e.g., bulb-light) and unrelated prime-target words (e.g., sock-light). Bayesian linear mixed-effects models over response times (RTs) revealed a main direct priming effect (faster RTs in related pairs than in unrelated ones), a main facilitatory effect for some EUB scores (i.e., the higher the value for EUB score, the faster RTs), and an interactive effect between the experimental manipulation and some EUB scores (the higher the EUB score, the smaller the direct priming effect). These

results are consistent with predictions made from the enhanced spreading of activation explanatory mechanism, but other alternative accounts are also discussed.

Keywords: associative priming, semantic priming, paranormal beliefs, pseudoscientific beliefs, conspiracy beliefs.

1. Model construction/selection

Data were analysed with linear mixed-effects models (LMEMs), which have several advantages over more traditional analysis such as separated by-participant and by-item ANOVAs and linear regressions (see Baayen et al., 2008; Brown, 2021; Liben-Nowell et al., 2019). Model construction/selection was performed in two steps. Firstly, we specified fixed-effects structure based on our theoretical assumptions. Critical predictors were set based on our aims and predictions, while control predictors were specified based on the identification of potential confounding variables in the literature. Secondly, the maximal random-effects structure motivated by experimental design (Barr et al., 2013) was intended.

1.1. Fixed-effects structure

Starting from the *enhanced spreading of activation* explanatory mechanism (e.g., Kiang, 2010; Kreher et al., 2008; Kuperberg, 2010; Rodríguez-Ferreiro et al., 2020) and the *psychosis continuum* (e.g., Galbraith, 2021, van Os et al., 2009; dimensional approach to psychopathology, e.g., Avasthi et al., 2014), our main aim was to explore whether there are individual differences in associative/semantic priming in people with different levels of epistemically unwarranted beliefs (EUB; Lobato et al., 2014). With this purpose in mind, participants whose EUB levels were measured through self-report psychometric instruments performed a primer lexical decision task (LDT). Our critical predictors were:

- **prime-target Relatedness (related vs. unrelated).** In each trial of the main task, participants had to perform a LDT over a *target* stimulus that could be either a real Spanish word (critical trials) or a pseudoword (filler trials). Response times (RTs) and response accuracy (RA)[1] were registered in DMDX (Forster & Forster, 2003). These target stimuli were briefly preceded by a *prime* stimulus each, that was always a real Spanish word. In the critical trials, prime-target words could be

[1] Only RTs were analysed (but not RA). A first reason for this is that priming effects are mainly seen in RTs. A second reason for this is that not many errors were expected to be made by participants, so not enough variability would be available to estimate reliable effects.

associatively/semantically related (e.g., bulb-light) or unrelated (e.g., sock-light)[2]. We expected to replicate the typical direct priming effect (i.e., faster RTs for related prime-target pairs than for unrelated ones).

- **EUB.** Six EUB dimensions were measured for each participant using PEUBI (Huete-Pérez et al., 2022) and the revised version of PSEUDO (Fasce et al., 2021): Superstitions (PEUBI-S); Occultism and Pseudoscience (PEUBI-OP); Traditional Religion (PEUBI-TR); Extraordinary Life Forms (PEUBI-ELF); Conspiracy Theories (PEUBI-CT); and Pseudoscientific beliefs (PSEUDO-R). Following the enhanced spreading of activation explanatory mechanism, we hypothesised that if EUB believers generally experience a faster/greater and further reaching spreading of activation through semantic memory (e.g., Kreher et al., 2008; Kuperberg, 2010) compared to people with low levels of EUB, a main facilitatory effect of EUB would be expected. That is, high scores in EUB should be associated with shorter RTs in both related (faster/greater spreading to close associates; see Kiang, 2010) and unrelated (further reaching spreading, which results in the activation of remote associates; see Kiang, 2010) conditions.

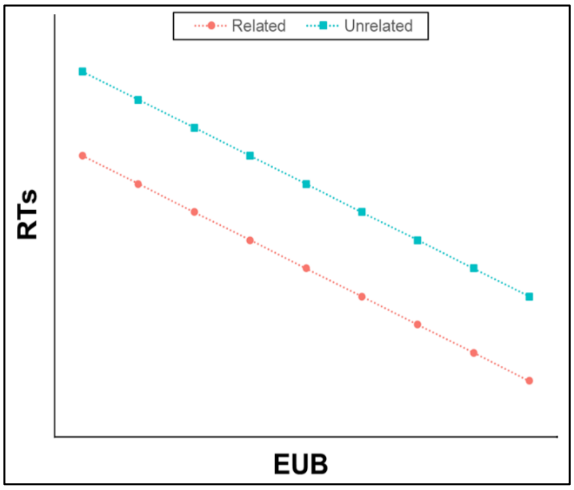

[2] In the related condition, the target word was the first associate of the prime word according to data from NALC free association norms (Díez et al., 2018; Fernández et al., 2004, 2019). In the unrelated condition, prime words were not associated with target words according to the same free association norms. Unrelated primes were selected using the Match software (van Casteren & Davis, 2007) starting from the related primes. Related and unrelated primes did not significantly differ in any of the word properties considered at the group-level on either their means (independent samples t-tests) or their distribution (two-sample independent Kolmogorov-Smirnov tests).

- **Relatedness x EUB.** Alternatively, if EUB facilitatory effects only occurs in one of these two conditions (exclusively in related pairs or in unrelated pairs), that would suggest that only one of two possible mechanisms are in place: faster/greater spreading to close associates or further reaching spreading activating remote associates. This would be indicated by an interaction between EUB and Relatedness.

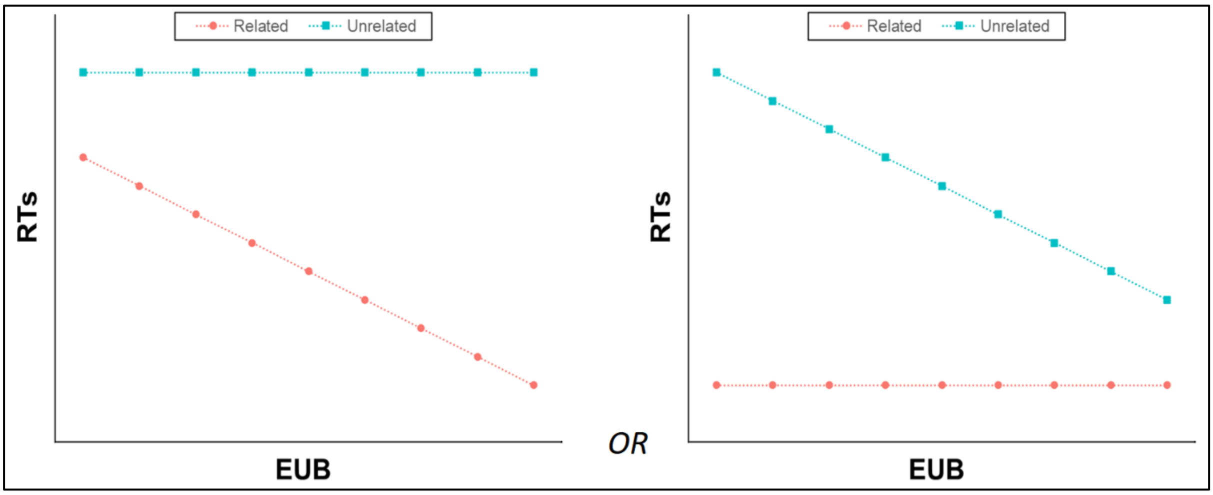

If we only considered these critical predictors in our analysis, we would be facing a serious threat to the validity of the results and conclusions: we know several other variables which are probably affecting lexical decisions that should be controlled for. Therefore, the following control predictors were also considered:

- **Word properties.** Psycholinguistics literature has identified many word properties that affect the ease with which a word is processed/recognised, such as prevalence (i.e., word's knowledge ratio in the population), lexical frequency (i.e., frequency to which a word appears in real context), sublexical frequency (i.e., how common is a pair/trio of letters in words of a given language), length (i.e., how many letters/phonemes/syllables has a word), lexical neighbourhood (i.e., number of orthographically/phonologically similar words), cognate similarity (i.e., lexical similarity between translations), age of acquisition (i.e., normative age at which a word is learnt), concreteness (i.e., degree to which a word is concrete or abstract), familiarity (i.e., perceived frequency of use/encounter), valence (i.e., degree to which

a word is emotionally positive-neutral-negative), and arousal (i.e., degree to which a word causes internal activation/excitement). For more information of these variables and their effects, see for instance Adelman (2012), Brysbaert et al. (2016), Pexman (2012), and Yap and Balota (2015).

- **Trial order.** As participants progress through the experiment, they can either improve their performance (e.g., learning/practice effects) or worsen it (e.g., fatigue effects). Including the trial number in the statistical analysis is better for reducing this confounding than simply relying on cancelling effects through randomization (see Baayen et al., 2008).
- **Previous trial.** Previous trial performance (such as previous RT and previous RA) affects the performance of the current trial in the lexical decision task (see Baayen et al., 2008).
- **List.** Each of the critical target words (e.g., light) had two possible prime words: one related (e.g., bulb-light) and one unrelated (e.g., sock-light). We did not consider it appropriate for the same participant to see both versions (is the target word processed differently the second time? For a discussion of the possible effects of repetition, see McNamara, 2005). Consequently, we created two experimental lists (A vs. B) with one prime-target version in each list. Each participant was randomly assigned to one of the two lists.
- **Relatedness x List.** This interactive effect was also added to the analysis to check that the experimental manipulation (i.e., Relatedness) is not confounded with the counterbalancing procedure (i.e., different pattern of Relatedness effects in each list).

1.2. Random-effects structure

RTs and RA observations produced in this study cannot be deemed to be independent. More specifically, the observations are grouped by-participant (i.e., the different responses of any given participant are related) and by-target (i.e., the different responses of any given

target word are related)[3]. Including this information in the statistical model improves its performance (McElreath, 2020, Chapter 13). In this sense, the maximal random-effects structure justified by design (Barr et al., 2013) would include:

- **Random intercepts (by-participant + by-target).** It seems implausible to assume that all participants or all target words have the same average/basal RTs or RA (regardless of stimulus to which one responds, there are people faster and/or with more tendency to respond 'yes' or 'no' than others; regardless the person who responds, there are items whose characteristics make them more easily to be processed/identified than others). Consequently, the most appropriate approach is letting intercepts to vary across these grouping units/factors.
- **Random slopes for Relatedness (by-participant + by-target).** Analogously to what happens with intercepts, one can argue that the effect of a predictor does not necessarily need to be identical for all participants or items. Random slopes for Relatedness could be estimated both by-participant and by-target, given that each instance of these grouping units have passed through the two conditions of Relatedness (i.e., allowing the estimation of the Relatedness effect for each participant and for each target word).
- **Random slopes for EUB (by-target).** Random slopes for EUB could be estimated by-target (i.e., each instance of this grouping unit has passed through different values of EUB). However, by-participant random slopes for EUB are *unidentifiable* (Barr et al., 2013), given that each participant has a unique value of EUB (i.e., no variation in the levels of EUB to estimate its effect in each participant).
- **Random slopes for Relatedness x EUB (none).** Random slopes for the Relatedness x EUB interactive effect are unidentifiable both by-participant (i.e., each participant

[3] One could wonder whether prime words could be included as another grouping factor. However, in line with previous priming studies without repetition, primes are simply considered as the operationalization of the Relatedness (related vs. unrelated) experimental manipulation.

passed through the two conditions of Relatedness, but has no level variation in EUB) and by-target (i.e., although each target word has passed through the two levels of Relatedness and different EUB values, given the counterbalanced design, it is implausible to assume that all possible cells of the interaction are available for each target word[4], so this variance component cannot be captured adequately).

As mentioned in Barr et al. (2013), random slopes for control predictors[5] could also be included in any LMEM, but they are not essential. Given the increase in model complexity and its associated estimation and computational problems, we follow the convention of only considering random slopes for critical predictors of the study.

1.3. Overview of the model formula

From all that has been said above, a general view of the model formula would be:

RTs ~ 1 + Prime word properties + Target word properties + Trial order + Previous trial + Relatedness + List + EUB + Relatedness:List + Relatedness:EUB + (1 + Relatedness | Participant) + (1 + Relatedness + EUB | Target)

The following word properties were considered: age of acquisition, concreteness, familiarity, valence, arousal, normalised Levenshtein distance between Spanish–Catalan translations, prevalence, two variables of lexical frequency (word frequency, contextual diversity), number of letters, three variables of lexical neighbourhood (number of orthographic neighbours, number of orthographic neighbours of higher frequency, mean Levenshtein distance of the 20 closest words), and two variables of sublexical frequency (brigram frequency, trigram frequency). Although all of them could be included in the

[4] As an illustrative example, for a PEUBI-S score of 10 the target word *abuelo* only has data for the related condition (list A): there are no participants with that specific PEUBI-S value in list B (where *abuelo* is under the unrelated condition).

[5] More concretely, the following random slopes for control predictors would be justified by design in this study: prime word properties (by-participant), target word properties (by-participant), trial order (by-participant + by-target), previous trial (by-participant + by-target), list (by-target).

analysis, proxies of the same construct were expected to be collinear[6] (i.e., the two variables of lexical frequency; the three variables of lexical neighbourhood; and the two variables of sublexical frequency). Therefore, a preventive removal of variables was performed: only one of each of these variables was finally included in the analyses. We selected word frequency as the lexical frequency measure; number of orthographical neighbours as the lexical neighbourhood measure; and bigram frequency as the sublexical frequency measure.

Regarding previous trial, we had two different variables to operationalise its effects: previous RT and previous RA.

In relation to EUB scores, we performed a separate analysis incorporating each possible EUB score on its own (PEUBI-S, PEUBI-OP, PEUBI-TR, PEUBI-ELF, PEUBI-CT, PSEUDO-R): the same analysis was repeated six times, but with varying EUB score. To the extent that EUB scores tend to be correlated (e.g., Huete-Pérez et al., 2022), the threat of collinearity prevented us to include all of them in a single analysis. Additionally, extracting a single score per participant using either sum-scores or dimension reduction techniques (e.g., PCA) was not deemed appropriate since different profiles would be masked: two individuals with the same global score could be very different in their EUB endorsement (e.g., person A high-superstition and low-conspiracy, person B low-superstition and high-conspiracy). Finally, considering that specific EUB types do not necessarily share the same underlying mechanisms (e.g., see Bensley, 2020; Huete-Pérez et al., 2023, Discussion section; Rizeq et al., 2020), we considered it important to be able to explore each EUB dimension separately.

1.4. Additional assumptions

- **Linear effects.** Notwithstanding that there is debate regarding whether the effects of some word properties are linear or non-linear (e.g., valence; see Hinojosa et al.,

[6] Even though the theoretical rationale is enough for excluding some variables, this question was further checked analytically. More concretely, pairs of word properties with a Pearson correlation coefficient of an absolute value ≥ .70 (Dormann et al., 2013) were deemed problematic. It should be noted that if a pair of variables exceeded the threshold in only one type of words (≥ .70 in prime words but not in target words, or viceversa), the excluded variable was removed both for primes and for targets.

2020), in the present data analyses only linear effects were considered.

- **Without interactions between word properties.** Even though there are some studies reporting interactive effects between word properties (e.g., word frequency x imageability/concreteness, González-Nosti et al., 2014), in the present analyses only main effects were considered.

1.5. What effects do we had a strong expectation to obtain?

Among the predictors included in the analysis, some of them have more consistent effects than others in word recognition literature (probably in part because they are big-sized effects). More concretely, we had strong expectation to obtain specific effects for:

- **Word frequency (target words).** We expected to replicate the typical facilitatory effect (i.e., the more frequent the word, the faster RTs).
- **Relatedness.** We expected to replicate the typical direct priming effect (i.e., faster RTs for related prime-target pairs that for unrelated ones).

Given that these effects are well stablished in the literature, the inability to obtain them could suggest potential unreliability/invalidity of our experimental procedure, data or statistical analysis.

1.6. What if we obtain null effects for the predictors of interest?

When null effects are obtained for a particular predictor, this does not necessarily imply that the effects do not exist: we could have been unable to accurately estimate and detect them. In the following, different alternative explanations are exposed regarding what could be happening if EUB main effects or Relatedness x EUB interactive effects ended being null:

- **No real relationship (true negative).** It is possible that there really are no individual differences by EUB (i.e., EUB has no main or interactive effects over primed LDT RTs).

- **Non-linear effects.** It is possible that the effects of EUB over primed LDT RTs do exist but in a non-linear fashion. If that was the case, we would not accurately estimate and detect these effects since we are considering EUB effects to be linear.
- **Insufficient statistical power.** It is possible that the effects of EUB over primed LDT do exist, but that they are so small that our study was not able to accurately estimate and detect them.
- **Sampling bias.** It is possible that the effects of EUB over primed LDT RTs do exist, but that the sample of participants or items are not representative of the population.
- **Attenuation of relationships: measurement error.** It is possible that the effects of EUB over primed LDT RTs do exist, but that the measures used are not reliable enough. To provide information in this regard, reliability estimates were reported for both EUB scores (McDonald's ω and Cronbach's α) and RTs (split-half).

```
### Split-half reliability (Spearman-Brown corrected) for RTs after data
trimming

splithalf(data = data, outcome = "RT", score = "average", halftype = "random",
permutations = 5000, var.RT = "rt", var.condition = "Relatedness",
conditionlist = c("Related","Unrelated"), var.participant = "subj", average =
"mean", plot = TRUE)
```

- **Attenuation of relationships: insufficient variability.** It is possible that the effects of EUB over primed LDT RTs do exist, but that for some EUB scores there is not enough variability in our sample (e.g., PEUBI-TR tends to present a high positive/right skewness because young participants tend to be mainly non-religious). To provide information in this regard, descriptive statistics of each EUB score were reported.
- **Uncontrolled variables.** It is possible that the effects of EUB over primed LDT RTs do exist, but they were masked by the effects of not controlled variables.

2. Data trimming

First, participants with an overall task error rate of >25% were excluded from the data analysis. This is done because participants who make a lot of mistakes may be indicative of

not having understood the task, not paying attention (even answering randomly), not having enough vocabulary level...

```
### Participants with >25% of errors are removed
dataRAW = dataRAW[dataRAW$subjERR <= 25,]
```

Second, filler items were only included to have the same probability of ‘yes’ or ‘no’ responses in the LDT, but they are not of interest. Consequently, only data from critical items were included in the analysis.

```
### Only critical items (i.e., real Spanish word as target) are analysed
dataRAW = dataRAW[dataRAW$desc == "critical_trials",]
```

Third, items with an overall task error rate >70% were excluded from the analysis. This is done because such a high error rate would imply that the real Spanish target word is generally not known by the sample of participants.

```
### Items with >70% of errors are removed
dataRAW = dataRAW[dataRAW$itemERR <= 70,]
```

Fourth, observations with display problems were removed (e.g., prime of a particular item being displayed longer than the set 200 ms for a particular participant). DMDX also registers when a trial had display errors, so these observations can be identified.

```
### Trials that were not correctly displayed/visualized are removed
dataRAW = dataRAW[dataRAW$display_error == "no",]
```

Fifth, only RTs of correct responses were analysed. If we start from the rationale that correct RT for a real word partly represents time needed to process and access it in participant’s lexicon (e.g., Libben, 2008), an incorrect response could imply different situations that are not of interest (e.g., involuntary motor or impulsive guessing responding before completely processing/activating/accessing the word, word not being represented in the lexicon…).

```
### Only RTs of correct responses are analysed
dataRAW = dataRAW[dataRAW$error == 0,]
```

Sixth, absolute cut-offs were applied: RTs faster than 300 ms or that reached the 2,000 ms limit were removed. Although the specific low cut-off value varies across studies (i.e., between 200 and 300 ms), these fast RTs are traditionally removed because they are interpreted as involuntary motor or impulsive guessing responding before completely processing and accessing the word (e.g., “fast guess” in Ratcliff & Hendrickson, 2021). RTs of 2,000 ms were removed because they are not real responses of participants, but the software automatically ending the trial because of reaching the time limit to respond.

```
### Absloute cut-offs: RTs performed before 300ms or that reached the time limit (2000ms) are removed

dataRAW = dataRAW[dataRAW$rt >= 300 & dataRAW$rt < 2000,]
```

Seventh, relative cut-offs were applied: RTs beyond 2.5 standard deviations of each participant’s mean were removed. Although the specific relative cut-off varies across studies (i.e., between 2 and 3 SDs), these extreme RTs are traditionally removed because of they are interpreted as outliers.

```
### Relative cut-offs: RTs that exceeded ±2.5 SD of each participant's mean are removed

dataRAW = as.data.frame(dataRAW)

data = perSubjectTrim.fnc(dataRAW, "rt", "subj", trim = 2.5)$data
```

3. Variables adjustment

First, it should be explicitly noted that analyses were performed on raw RTs. We are aware that, given the positive/right skewness of RTs distribution, they are sometimes transformed attempting to normalize the distribution (e.g., inverse and logarithmic transformations). However, concerns have been raised regarding the possibility of obtaining spurious results (both false positives and false negatives) in analyses over non-linearly transformed RTs (see Lo & Andrews, 2015; Schramm & Rouder, 2019). Although LMEMs seem to be robust to violations of distributional assumptions (see Schielzeth et al., 2020), a better solution than transforming RTs is to analyse the data using a reference distribution more like the typical right-skewed RTs distribution, such as Ex-Gaussian and Inverse

Gaussian distributions (e.g., Heathcote et al., 1991; Lo & Andrews, 2015; see also Lindeløv, 2019).

Second, to avoid that categorical variables are erroneously interpreted as numerical variables in RStudio, we applied the `as.factor()` function to all categorical variables.

```
### Defining which variables are categorical (even if not used in analysis)
data$subj = as.factor(data$subj)
data$desc = as.factor(data$desc)
data$itemN = as.factor(data$itemN)
data$error = as.factor(data$error)
data$display_error = as.factor(data$display_error)
data$prevERR = as.factor(data$prevERR)
data$prevYES = as.factor(data$prevYES)
data$ID = as.factor(data$ID)
data$Prime = as.factor(data$Prime)
data$Target = as.factor(data$Target)
data$Relatedness = as.factor(data$Relatedness)
data$List = as.factor(data$List)
data$Sex = as.factor(data$Sex)

str(data) #Checking the type of each variable
```

Third, we sum-coded the categorical variables that were going to be used as predictors in the model (for more information about contrast coding alternatives and their implications, see Brehm & Alday, 2022).

```
### Sum coding [-1, +1] for the two levels of categorical variables that will
be included as fixed effects predictors
contrasts(data$prevERR) = contr.sum(levels(data$prevERR))
contrasts(data$Relatedness) = contr.sum(levels(data$Relatedness))
contrasts(data$List) = contr.sum(levels(data$List))

contrasts(data$prevERR) # -1 = yes, +1 = no
contrasts(data$Relatedness) # -1 = Unrelated, +1 = Related
contrasts(data$List) # -1 = B, +1 = A
```

Fourth, we transformed sublexical frequencies to a $\log_{10}$ scale. The relationship between RTs and lexical and sublexical frequencies seems to follow a logarithmic scale (e.g., see Adelman, 2012; Massaro et al., 1980, Chapter 5). Although lexical frequency measures were already obtained $\log_{10}$-transformed, sublexical frequency measures were obtained in raw values.

```
### Log10 transformation of bigram and trigram frequencies
data$P_abs_tok_MBOF = log10(data$P_abs_tok_MBOF)
data$P_abs_tok_MTOF = log10(data$P_abs_tok_MTOF)
data$T_abs_tok_MBOF = log10(data$T_abs_tok_MBOF)
data$T_abs_tok_MTOF = log10(data$T_abs_tok_MTOF)
```

Fifth, we standardized all possible continuous variables to both ease interpretation (i.e., all continuous predictors had the same scale: *M* = 0 and *SD* = 1) and reduce non-essential collinearity due to scaling for interactive terms with their lower-order components (Cohen et al., 2003, Chapter 6).

```
### Standardizing all possible continuous variables (even if not used in analysis), except the DV (i.e., RTs)
data$trial = standardize(data$trial)
data$prevRT_auto = standardize(data$prevRT_auto)
data$prevRT_man = standardize(data$prevRT_man)
data$itemERR = standardize(data$itemERR)
data$subjERR = standardize(data$subjERR)
data$FSG = standardize(data$FSG)
data$Age = standardize(data$Age)
data$PEUBI_S = standardize(data$PEUBI_S)
data$PEUBI_OP = standardize(data$PEUBI_OP)
data$PEUBI_TR = standardize(data$PEUBI_TR)
data$PEUBI_ELF = standardize(data$PEUBI_ELF)
data$PEUBI_CT = standardize(data$PEUBI_CT)
data$PSEUDO_R = standardize(data$PSEUDO_R)
data$P_AoA = standardize(data$P_AoA)
data$P_Conc = standardize(data$P_Conc)
data$P_Fam = standardize(data$P_Fam)
```

```
data$P_Val = standardize(data$P_Val)
data$P_Aro = standardize(data$P_Aro)
data$P_NLD_Spanish_Catalan = standardize(data$P_NLD_Spanish_Catalan)
data$P_prevalence_nts = standardize(data$P_prevalence_nts)
data$P_log_frq = standardize(data$P_log_frq)
data$P_num_letters = standardize(data$P_num_letters)
data$P_N = standardize(data$P_N)
data$P_NHF = standardize(data$P_NHF)
data$P_Lev_N = standardize(data$P_Lev_N)
data$P_abs_tok_MBOF = standardize(data$P_abs_tok_MBOF)
data$P_abs_tok_MTOF = standardize(data$P_abs_tok_MTOF)
data$P_log_Ctx_div = standardize(data$P_log_Ctx_div)
data$T_AoA = standardize(data$T_AoA)
data$T_Conc = standardize(data$T_Conc)
data$T_Fam = standardize(data$T_Fam)
data$T_Val = standardize(data$T_Val)
data$T_Aro = standardize(data$T_Aro)
data$T_NLD_Spanish_Catalan = standardize(data$T_NLD_Spanish_Catalan)
data$T_prevalence_nts = standardize(data$T_prevalence_nts)
data$T_log_frq = standardize(data$T_log_frq)
data$T_num_letters = standardize(data$T_num_letters)
data$T_N = standardize(data$T_N)
data$T_NHF = standardize(data$T_NHF)
data$T_Lev_N = standardize(data$T_Lev_N)
data$T_abs_tok_MBOF = standardize(data$T_abs_tok_MBOF)
data$T_abs_tok_MTOF = standardize(data$T_abs_tok_MTOF)
data$T_log_Ctx_div = standardize(data$T_log_Ctx_div)

describe(data) #Checking standardization
```

4. Frequentist LMEMs specifications

4.1. Model formula in *lme4* package + Random-effects structure

When using the *lme4* package (Bates et al., 2015), convergence and singularity issues are quite common for complex random-effects structures (Meteyard & Davies, 2020). In

many cases this prevents to use the maximal random-effects structure justified by design (Barr et al., 2013). It must be noted that how to address convergence and singularity issues is still not standardized (Bates et al., 2015; Meteyard & Davies, 2020). In the present study, in case of a maximal model returning convergence or singular fit warnings, the maximal random-effects structure was iteratively simplified by dropping the random effect with lower variance (until there were no convergence or singularity issues)[7].

```
### Determining the random effects structure that does not produce warnings
starting from the maximal justified by design (Barr et al., 2013) partially
following Bates et al. (2018)

Model = lmer(rt ~ 1 + P_AoA + P_Conc + P_Fam + P_Val + P_Aro +
P_NLD_Spanish_Catalan + P_prevalence_nts + P_log_frq + P_num_letters + P_N
+ P_abs_tok_MBOF + T_AoA + T_Conc + T_Fam + T_Val + T_Aro +
T_NLD_Spanish_Catalan + T_prevalence_nts + T_log_frq + T_num_letters + T_N
+ T_abs_tok_MBOF + trial + prevRT_man + prevERR + Relatedness + List + EUB
+ Relatedness:List + Relatedness:EUB + (1 + Relatedness | subj) + (1 +
Relatedness + EUB | Target), data = data))

# Warning? Refit the Model dropping the random effect with lower variance
```

As previously stated, analyses were performed on raw RTs (see *Variables adjustment* section). The default `lmer()` reference distribution was used (i.e., Gaussian distribution).

4.2. Model checks

LMEMs statistical assumptions were checked with *performance* package (Lüdecke et al., 2021): absence of outliers, linearity, normality, homoscedasticity, and absence of collinearity.

```
### Model assumptions check

check_outliers(Model)

check_model(Model)
```

It must be said that LMEMs seem to be robust to violations of distributional assumptions (see Schielzeth et al., 2020).

[7] This strategy is an adaptation of the proposal described by Bates et al. (2018). More concretely, their proposal implies the following sequence of steps until the model does not produce convergence or singularity warnings. First, fit the maximal model. Second, refit the model forcing correlations between random effects to be zero. Third, refit the model removing the random effect with lower variance (repeating this step as many times as necessary). Finally, when the model is already non-problematic, allow the remaining random effects to be correlated (if problematic once again, undo this step). In our case we skipped the part of forcing random effects to be uncorrelated, given that this seems not to work well with categorical predictors.

4.3. Extracting information from the model

Following the tables for reporting LMEMs of Meteyard and Davies (2020), information from fixed effects' estimates, random effects' estimates and model fit were extracted from the model.

```
### Extracting information from the model

summary(Model)

confint.merMod(Model, level = 0.95, method = c("Wald"), quiet = FALSE,
oldNames = FALSE) #Obtain the confidence intervals of fixed effects

r2(Model) #Obtain the proportion of variance explained by the model (R²)
```

Although main effects are relatively easy to be directly interpreted from the slope coefficient (i.e., b > 0 implies that the higher the value of the predictor, the higher/slower RTs; a b < 0 implies that the higher the value of the predictor, the smaller/faster RTs), interactive effects are not that intuitive and it is better to plot them.

```
### Plotting the effects of interest

plot(ggemmeans(Model, terms = c("EUB[-2,-1.5,-1,-0.5,0,0.5,1,1.5,2]",
"Relatedness"), ci_level = 0.95)) + ylim(450,650) #Estimated marginal means
and their 95% CI
```

5. Bayesian LMEMs specifications

Bayesian LMEMs have been argued to have many advantages over frequentist LMEMs, such as more flexibility in model specification, quantification of uncertainty around estimated parameters, possibility of including prior knowledge, or avoiding the problems associated with multiple comparisons (Nalborczyk et al., 2019; Vasishth et al., 2018). However, on a more practical level, probably the greatest advantage is that, when using the *brms* package (Bürkner, 2017), Bayesian LMEMs do not present the convergence or singularity issues of frequentist LMEMs regarding fitting the maximal random-effects structure justified by design (e.g., see Nalborczyk et al., 2019; Vasishth et al., 2018).

5.1. Selecting the reference distribution for the response/dependent variable

As previously stated, analyses were performed on raw RTs (see *Variables adjustment*

section). To account for the typical positive/right skewness of RTs distribution, we opted to use an Ex-Gaussian distribution, since it seems specifically adequate to model RTs of speeded two-choice tasks (see Matzke & Wagenmakers, 2009). Posterior predictive checks were performed over models to ensure that this distribution was appropriate to our data (see *Model checks* subsection).

5.2. Prior distributions

Weakly informative priors seem to be a good choice when specifying prior distributions for the parameters of the model: they provide a bit of information by discarding very unlikely extreme values (which contributes to model regularization, that is, to avoid overfitting), but they still allow a wide range of values (which lets the data to influence the model to a great extent, thus avoiding underfitting) (see McElreath, 2020, Chapter 7; Stan Development Team, 2023; Vasishth et al., 2018).

```
### Custom priors
custom_priors = c(set_prior("normal(700, 200)", class = "Intercept"),
                  set_prior("normal(0, 50)",    class = "b"),
                  set_prior("normal(0, 50)",    class = "sd"),
                  set_prior("lkj(2)",           class = "cor"))
```

- **Intercept ~ Normal(700, 200).** With this prior distribution we considered that 95% of the values for intercepts would lie between [300, 1100] ms, given that the 95% of the values of a normal distribution lie ±2 *SD* of the *M*. The lower limit of 300 ms was driven by of our absolute cut-offs (see *Data trimming* section). The upper limit of 1100 ms was driven by our previous experience regarding lexical decision task (i.e., average/mean RTs are unlikely to be more than 1 s).[8]

```
### Visualizing the prior distribution N(700,200)
library(BayesTools)
```

[8] A weakly informative distribution for the Intercept could have been Normal(1150, 425), given the full range of possible values would be [300, 2000] ms given our absolute cut-offs. However, in this parameter we allowed ourselves to be more informative given our prior knowledge of the phenomenon.

```
plot(prior(distribution = "normal", parameters =
list(mean = 700, sd = 200)))
```

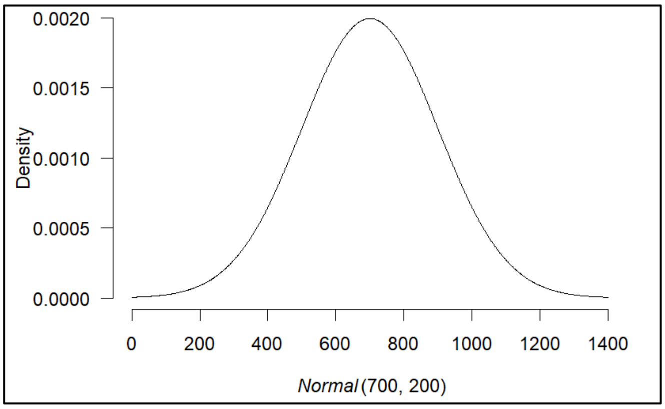

- **Slopes ~ Normal(0, 50).** With this prior distribution we considered that 95% of the values for fixed effects estimates would be between [-100, +100] ms by unit change in the predictor. Given the RTs scale, a predictor involving an effect of ±100 ms by unit change would be surprisingly very large (e.g., see González-Nosti et al., 2014).

```
### Visualizing the prior distribution N(0,50)

library(BayesTools)

plot(prior(distribution = "normal", parameters =
list(mean = 0, sd = 50)))
```

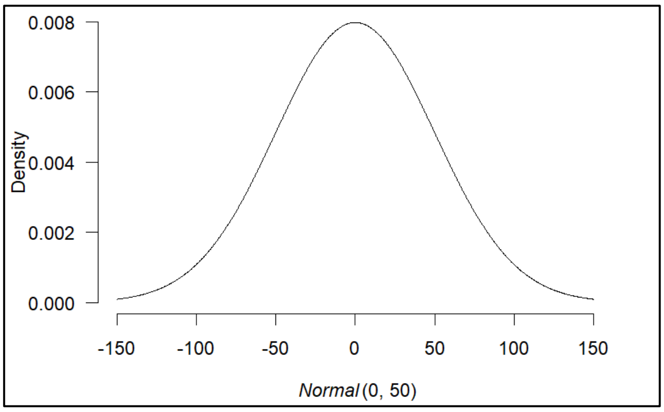

- **Standard deviation for random effects ~ Normal$_{+}$(0, 50).** With this prior distribution we considered that 95% of the values for standard deviation estimates for random effects would be between [0, +100][9]. Therefore, we are assuming that variability in random effects is most likely to be 0, but that it could be large (although as its value becomes bigger, its likelihood decreases).

[9] The *brms* package automatically restricts `class = sd` estimates to positive values only. Therefore, you can specify any distribution, but considering that only values ≥0 will be allowed.

```
### Visualizing the prior distribution N+(0,50)

library(BayesTools)

plot(prior(distribution = "normal", parameters =
list(mean = 0, sd = 50), truncation = list(0, Inf)))
```

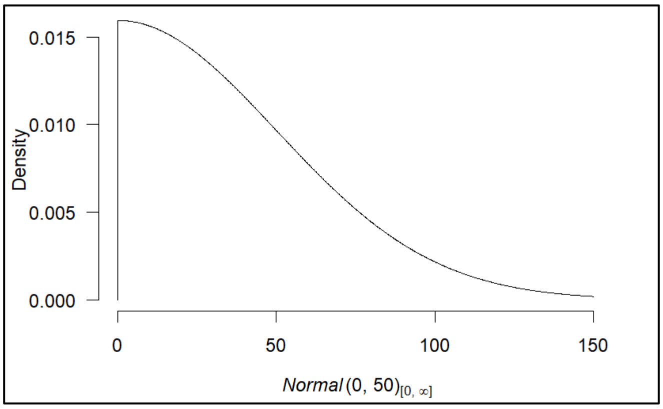

- **Correlation between random effects ~ LKJ(2).** The default LKJ(1) prior of *brms* implies that all the range of correlation values (i.e., [-1, +1]) are equiprobable (see Bürkner, 2017). However, following Nalborczyk et al. (2019) and Vasishth et al. (2018), by specifying LKJ(2) we considered that perfect correlations (i.e., ±1.00) between random effects are very unlikely. This specification "ensures that the correlation parameter(s) can generally be estimated, even when data are relatively sparse" (Vasishth et al., 2018, p. 150), which is one of the underlying reasons for frequentist LMEMs returning warnings.

```
### Visualizing the prior distribution LKJ(2)

library(rethinking)

dens(rlkjcorr(n = 1000000, K = 2, eta = 2)[,1,2])
```

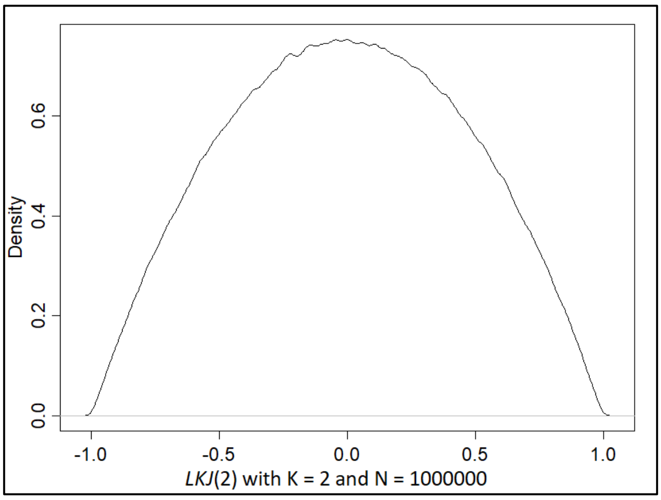

We tried to motivate our choices in prior distributions, but other people could disagree with us. Nevertheless, it must be highlighted that the influence of prior distributions over the posterior distribution decreases as the amount of data points increases (e.g., Kruschke, 2015, Chapter 6; Smid & Winter, 2020), so prior choices should not make a difference given the large amount of data points in this study (but caution with highly informative priors, see Smeets & van de Schoot, 2019)[10].

5.3. Model formula in *brms* package

The basic syntax of the *brms* package is identical to *lme4* package, but some distinct additional arguments may be needed to specify the Bayesian LMEMs:

```
### Fitting and saving the model with the maximal random effects structure
justified by design (Barr et al., 2013)

Model = brm(rt ~ 1 + P_AoA + P_Conc + P_Fam + P_Val + P_Aro +
P_NLD_Spanish_Catalan + P_prevalence_nts + P_log_frq + P_num_letters + P_N
+ P_abs_tok_MBOF + T_AoA + T_Conc + T_Fam + T_Val + T_Aro +
T_NLD_Spanish_Catalan + T_prevalence_nts + T_log_frq + T_num_letters + T_N
+ T_abs_tok_MBOF + trial + prevRT_man + prevERR + Relatedness + List + EUB
+ Relatedness:List + Relatedness:EUB + (1 + Relatedness | subj) + (1 +
Relatedness + EUB | Target), data = data, family = exgaussian(), prior =
custom_priors, iter = 10000, warmup = 2000, chains = 4, cores = 4)

saveRDS(Model, file = "Model.rds")
```

- **Family.** We set the Ex-Gaussian distribution as the reference distribution for RTs.
- **Prior.** We set the usage of prior distributions specified in the previous subsection (instead of *brms* default priors).
- **Iter.** We set the total number of iterations/samples that the MCMC procedure uses for

[10] Smeets & van de Schoot (2019) show another way to visualize prior distributions: the argument `sample_priors = "yes"` must be added to the `brm()` function (to indicate the MCMC procedure that samples must be drawn from the prior distributions too, since by default it only draws samples from posterior distributions) and, then, use the function `plot(hypothesis(Model, "predictor > 0"))`. For illustrative purposes, the code to plot the distributions for a slope (e.g., fixed effect of EUB score) would be `plot(hypothesis(Model, "EUB > 0", class = "b"))`, the code to plot the distributions for a standard deviation of a random effect (e.g., by-target random slopes for Relatedness) would be `plot(hypothesis(Model, "Target__Relatedness1 > 0", class = "sd"))`, and the code to plot the distributions for a correlation between random effects (e.g., correlation between by-target random slopes for Relatedness and EUB) would be `plot(hypothesis(Model, "Target__Relatedness1__EUB > 0", class = "cor"))`. However, this way of visualizing prior distributions has at least two disadvantages. First, the `sample_priors = "yes"` argument, in our experience, increased the time necessary to perform the MCMC procedure. Second, the prior distribution for the intercept cannot be sampled for technical reasons (see *brms* documentation) and, therefore, it won't be available to be visualized.

each independent sampling chain to 10,000 (*brms* default is 2,000)[11]. This value sums (1) the number of iterations/samples used for the warm-up phase and (2) the number of iterations/samples used for the Posterior distribution approximation phase (McElreath, 2020, Chapter 9).

- **Warmup.** We set the number of iterations/samples that the MCMC procedure uses for the warm-up phase in each chain/independent sampling to 2,000 (*brms* default is `Iter/2`)[11]. Given that these warm-up iterations/samples are not considered to approximate the Posterior distribution (Nalborczyk et al., 2019; Vasishth et al., 2018), in this case the number of iterations/samples used to approximate the Posterior distribution in each chain is `Iter – Warmup = 8000`.
- **Chains.** We set the number of independent sampling chains to 4 (corresponds to *brms* default)[12].
- **Cores.** We set the number of cores to 4 (instead of the *brms* default) to have all chains processed in parallel[13].
- **SaveRDS.** Since complex Bayesian LMEMs can take much time to fit (in this case, about 5 hours each model), it is appropriate to save it as a .rds file, enabling to charge it again later.

5.4. Model checks

Firstly, we checked **variance inflation factors (VIFs)** to ensure that there were no important degrees of collinearity between predictors.

```
### Model checks

check_collinearity(Model) #VIFs (all must be ≤5, and better if ≤3)
```

[11] The *brms* default number of iterations was first used (i.e., `iter = 2000`, `warmup = 1000`). However, since a low ESS warning appeared, we followed the recommendation of running more iterations (Stan Development Team, 2022).

[12] Given the stochastic nature of MCMC procedure, a single sampling chain could behave idiosyncratically and produce non-reliable results. Consequently, it is recommended to use >1 chain to ensure that separate independent samplings converge to the same solution (see McElreath, 2020, Chapter 9).

[13] The number of cores varies between computers, so you may need to adjust this value if you run this code. If you ask yourself how many cores has your computer, this can be checked with `detectCores()` function.

Secondly, we performed a **posterior predictive check** to ensure that the chosen reference distribution for the model was appropriate to our data.

```
### Model checks
pp_check(Model, ndraws = 100) #Posterior predictive check
```

Thirdly, some diagnostics were also inspected to check whether the MCMC procedure had any convergence and/or efficiency issue (see McElreath, 2020, Chapter 9): **trace and trank plots**, **R-hat ($\hat{R}$)** Gelman-Rubin convergence diagnostic, **effective sample size (ESS)**.

```
### Model checks
plot(Model, combo = c("trace","rank_overlay")) #Trace and trank plots (to see only one parameter at a time, add the argument N = 1)
summary(Model) #R-hat (all must be <1.01) + ESS (all must be >100 times the number of chains) --> https://mc-stan.org/misc/warnings
```

5.5. Extracting information from the model

Although the information from the model can be extracted in a tabulated form (as in frequentist LMEMs), at least for fixed effects it may be appropriate to additionally plot posterior distributions (keep in mind that in Bayesian LMEMs the emphasis is on the whole distribution of possible values, not just on central tendency point estimates).

```
### Extracting information from the model
summary(Model)
plot(Model, combo = c("dens","hist")) #Posterior distribution of parameters (to see only one parameter at a time, add the argument N = 1)
plot(eti(Model, effects = "fixed", ci = 0.95)) + xlim(-40,40) #Posterior distributions of fixed effects with 95% CrI (in terms of Equally-Tailed Intervals)
```

As in frequentist LMEMs, interactive effects were plotted to better comprehend them.[14]

```
### Plotting the effects of interest
plot(ggemmeans(Model, terms = c("EUB[-2,-1.5,-1,-0.5,0,0.5,1,1.5,2]", "Relatedness"), ci_level = 0.95)) + ylim(475,600) #Estimated marginal medians and their 95% CrI (in terms of Highest Posterior Density Interval)
```

[14] In this case, although the formula is the same as in frequentist LMEMs, with Bayesian LMEMs point estimates are medians (instead of means) and the CI argument produces credible intervals (instead of confidence intervals).

6. References

Adelman, J. S. (2012). Methodological issues with words. In: J. S. Adelman (Ed.) *Visual word recognition (vol. 1): Models and methods, orthography and phonology* (pp. 116-138). Psychology Press.

Avasthi, A., Sarkar, S., & Grover, S. (2014). Approaches to psychiatric nosology: A viewpoint. *Indian Journal of Psychiatry, 56*(3), 301-304. https://doi.org/10.4103/0019-5545.120560

Baayen, R. H., Davidson, D. J., & Bates, D. M. (2008). Mixed-effects modeling with crossed random effects for subjects and items. *Journal of Memory and Language, 59*(4), 390-412. https://doi.org/10.1016/j.jml.2007.12.005

Barr, D. J., Levy, R., Scheepers, C., & Tily, H. J. (2013). Random effects structure for confirmatory hypothesis testing: Keep it maximal. *Journal of Memory and Language, 68*(3), 255-278. https://doi.org/10.1016/j.jml.2012.11.001

Bates, D., Kliegl, R., Vasishth, S., Baayen, R. H. (2018). *Parsimonious mixed models.* arXiv. https://doi.org/10.48550/arXiv.1506.04967

Bates, D., Mächler, M., Bolker, B. M., Walker, S. C. (2015). Fitting linear mixed-effects models using lme4. *Journal of Statistical Software, 67*(1), 1–48. https://doi.org/10.18637/jss.v067.i01

Bensley, D. A., Lilienfeld, S. O., Rowan, K. A., Masciocchi, C. M., & Grain, F. (2020). The generality of belief in unsubstantiated claims. *Applied Cognitive Psychology, 34*(1), 16-28. https://doi.org/10.1002/acp.3581

Brehm, L., & Alday, P. M. (2022). Contrast coding choices in a decade of mixed models. *Journal of Memory and Language, 125*, 104334. https://doi.org/10.1016/j.jml.2022.104334

Brown, V. A. (2021). An introduction to linear mixed-effects modeling in R. *Advances in Methods and Practices in Psychological Science, 4*(1), 2515245920960351. https://doi.org/10.1177/2515245920960351

Brysbaert, M., Stevens, M., Mandera, P., & Keuleers, E. (2016). The impact of word prevalence on lexical decision times: Evidence from the Dutch Lexicon Project 2. *Journal of Experimental Psychology: Human Perception and Performance, 42*(3), 441–458. https://doi.org/10.1037/xhp0000159

Bürkner, P. C. (2017). brms: An R package for Bayesian multilevel models using Stan. *Journal of Statistical Software, 80*(1), 1–28. https://doi.org/10.18637/jss.v080.i01

Cohen, J., Cohen, P., West, S. G., & Aiken, L. S. (2003). *Applied multiple regression/correlation analysis for the behavioral sciences* (3rd ed.). Routledge.

Díez, E., Alonso, M. A., Rodríguez, N., & Fernández, A. (2018). *Free-association norms for a large set of words in Spanish.* Unpublished. https://doi.org/10.13140/RG.2.2.17703.70560

Dormann, C. F., Elith, J., Bacher, S., Buchmann, C., Carl, G., Carré, G., García-Márquez, J. R., Gruber, B., Lafourcade, B., Leitão, P. J., Münkemüller, T., McClean, C., Osborne, P. E., Reineking, B., Schröder, B., Skidmore, A. K., Zurell, D., & Lautenbach, S. (2013). Collinearity: a review of methods to deal with it and a simulation study evaluating their performance. *Ecography, 36*(1), 27-46. https://doi.org/10.1111/j.1600-0587.2012.07348.x

Fasce, A., Avendaño, D., & Adrián-Ventura, J. (2021). Revised and short versions of the pseudoscientific belief scale. *Applied Cognitive Psychology, 35*(3), 828-832. https://doi.org/10.1002/acp.3811

Fernández, A., Díez, E., & Alonso, M. A. (2019). *Normas de Asociación Libre en Castellano (NALC) de la Universidad de Salamanca* [Online database]. Retrieved February 17, 2023, from http://campus.usal.es/gimc/nalc

Fernández, A., Díez, E., Alonso, M. A., & Beato, M. S. (2004). Free-association norms for the Spanish names of the Snodgrass and Vanderwart pictures. *Behavior Research Methods, Instruments, & Computers, 36*, 577-583. https://doi.org/10.3758/BF03195604

Forster K. I., & Forster, J. C. (2003). DMDX: A Windows display program with millisecond accuracy. *Behavior Research Methods, Instruments, & Computers, 35*(1), 116–124. https://doi.org/10.3758/BF03195503

Galbraith, N. (2021). Delusions and pathologies of belief: Making sense of conspiracy beliefs via the psychosis continuum. In V. Cardella & A. Gangemi (Eds.), *Psychopathology and Philosophy of Mind* (pp. 117-144). Routledge. https://doi.org/10.4324/9781003009856-8

González-Nosti, M., Barbón, A., Rodríguez-Ferreiro, J., & Cuetos, F. (2014). Effects of the psycholinguistic variables on the lexical decision task in Spanish: A study with 2,765 words. *Behavior Research Methods, 46*, 517-525. https://doi.org/10.3758/s13428-013-0383-5

Heathcote, A., Popiel, S. J., & Mewhort, D. J. (1991). Analysis of response time distributions: An example using the Stroop task. *Psychological Bulletin, 109*(2), 340-347. https://doi.org/10.1037/0033-2909.109.2.340

Hinojosa, J. A., Moreno, E. M., & Ferré, P. (2020). Affective neurolinguistics: towards a framework for reconciling language and emotion. *Language, Cognition and Neuroscience, 35*(7), 813-839. https://doi.org/10.1080/23273798.2019.1620957

Huete-Pérez, D., & Ferré, P. (2023). Individual differences in visual word recognition: the role of epistemically unwarranted beliefs on affective processing and signal detection. *Language and Cognition, 15*(2), 314-336. https://doi.org/10.1017/langcog.2022.38

Huete-Pérez, D., Morales-Vives, F., Gavilán, J. M., Boada, R., & Haro, J. (2022). Popular Epistemically Unwarranted Beliefs Inventory (PEUBI): A psychometric instrument for assessing paranormal, pseudoscientific and conspiracy beliefs. *Applied Cognitive Psychology, 36*(6), 1260-1276. https://doi.org/10.1002/acp.4010

Kiang, M. (2010). Schizotypy and language: A review. *Journal of Neurolinguistics, 23*(3), 193-203. https://doi.org/10.1016/j.jneuroling.2009.03.002

Kreher, D. A., Holcomb, P. J., Goff, D., & Kuperberg, G. R. (2008). Neural evidence for faster and further automatic spreading activation in schizophrenic thought disorder. *Schizophrenia Bulletin*, *34*(3), 473-482. https://doi.org/10.1093/schbul/sbm108

Kruschke, J. (2015). *Doing Bayesian data analysis: A tutorial with R, JAGS, and Stan* (2nd ed.). Academic Press.

Kuperberg, G. R. (2010). Language in schizophrenia part 1: an introduction. *Language and Linguistics Compass*, *4*(8), 576-589. https://doi.org/10.1111/j.1749-818X.2010.00216.x

Libben, G. (2008). Disorders of lexis. In B. Stemmer & H. A. Whitaker (Eds.), *Handbook of the Neuroscience of Language* (pp. 147-154). Elsevier. https://doi.org/10.1016/B978-0-08-045352-1.00014-8

Liben-Nowell, D., Strand, J., Sharp, A., Wexler, T., & Woods, K. (2019). The danger of testing by selecting controlled subsets, with applications to spoken-word recognition. *Journal of Cognition*, *2*(1), 2. https://doi.org/10.5334%2Fjoc.51

Lindeløv, J. K. (2019). *Reaction time distributions: An interactive overview*. https://lindeloev.github.io/shiny-rt/

Lo, S., & Andrews, S. (2015). To transform or not to transform: Using generalized linear mixed models to analyse reaction time data. *Frontiers in Psychology*, *6*, 1171. https://doi.org/10.3389/fpsyg.2015.01171

Lobato, E., Mendoza, J., Sims, V., & Chin, M. (2014). Examining the relationship between conspiracy theories, paranormal beliefs, and pseudoscience acceptance among a university population. *Applied Cognitive Psychology*, *28*(5), 617–625. https://doi.org/10.1002/acp.3042

Lüdecke, D., Ben-Shachar, M. S., Patil, I., Waggoner, P., Makowski, D., (2021). performance: An R package for assessment, comparison and testing of statistical models. *Journal of Open Source Software*, *6*(60), 3139. https://doi.org/10.21105/joss.03139

Massaro, D. W., Taylor, G. A., Venezky, R. L., Jastrzembski, J. E., Lucas, P. A. (1980). *Letter and word perception: Orthographic structure and visual processing in reading*. North-Holland.

Matzke, D., & Wagenmakers, E. J. (2009). Psychological interpretation of the ex-Gaussian and shifted Wald parameters: A diffusion model analysis. *Psychonomic Bulletin & Review, 16*, 798-817. https://doi.org/10.3758/PBR.16.5.798

McElreath, R. (2020). *Statistical rethinking: A Bayesian course with examples in R and STAN* (2nd Ed.). Champan and Hall/CRC Press. https://doi.org/10.1201/9780429029608

McNamara, T. P. (2005). *Semantic priming: Perspectives from memory and word recognition*. Psychology Press.

Meteyard, L., & Davies, R. A. I. (2020). Best practice guidance for linear mixed-effects models in psychological science. *Journal of Memory and Language, 112,* 104092. https://doi.org/10.1016/j.jml.2020.104092

Nalborczyk, L., Batailler, C., Lœvenbruck, H., Vilain, A., & Bürkner, P. C. (2019). An introduction to Bayesian multilevel models using brms: A case study of gender effects on vowel variability in standard Indonesian. *Journal of Speech, Language, and Hearing Research, 62*(5), 1225-1242. https://doi.org/10.1044/2018_JSLHR-S-18-0006

Pexman, P. M. (2012). Meaning-based influences on visual word recognition. In: J. S. Adelman (Ed.) *Visual word recognition (vol. 2): Meaning and context, individuals and development* (pp. 24-43). Psychology Press.

Ratcliff, R., & Hendrickson, A. T. (2021). Do data from mechanical Turk subjects replicate accuracy, response time, and diffusion modeling results? *Behavior Research Methods, 53*(6), 2302-2325. https://doi.org/10.3758/s13428-021-01573-x

Rizeq, J., Flora, D. B., & Toplak, M. E. (2020). An examination of the underlying dimensional structure of three domains of contaminated mindware: Paranormal

beliefs, conspiracy beliefs, and anti-science attitudes. *Thinking & Reasoning*, *27*(2), 187–211. https://doi.org/10.1080/13546783.2020.1759688

Rodríguez-Ferreiro, J., Aguilera, M., & Davies, R. (2020). Semantic priming and schizotypal personality: Reassessing the link between thought disorder and enhanced spreading of semantic activation. *PeerJ, 8*, e9511. https://doi.org/10.7717/peerj.9511

Schielzeth, H., Dingemanse, N. J., Nakagawa, S., Westneat, D. F., Allegue, H., Teplitsky, C., Réale, D., Dochtermann, N. A., Garamszegi, L. Z., Araya-Ajoy, Y. G. (2020). Robustness of linear mixed-effects models to violations of distributional assumptions. *Methods in Ecology and Evolution, 11*(9), 1141-1152. https://doi.org/10.1111/2041-210X.13434

Schramm, P., & Rouder, J. N. (2019). *Are reaction time transformations really beneficial?* https://doi.org/10.31234/osf.io/9ksa6

Smeets, L., & van de Schoot, R. (2019). *Influence of priors: Popularity data.* https://www.rensvandeschoot.com/tutorials/brms-priors/

Smid, S. C., & Winter, S. D. (2020). Dangers of the defaults: A tutorial on the impact of default priors when using Bayesian SEM with small samples. *Frontiers in Psychology*, *11*, 611963. https://doi.org/10.3389/fpsyg.2020.611963

Stan Development Team (2022). *Runtime warnings and convergence problems.* https://mc-stan.org/misc/warnings.html

Stan Development Team (2023). *Prior choice recommendations.* https://github.com/stan-dev/stan/wiki/Prior-Choice-Recommendations

van Casteren, M., & Davis, M. H. (2007). Match: A program to assist in matching the conditions of factorial experiments. *Behavior Research Methods, 39*(4), 973–978. https://doi.org/10.3758/BF03192992

van Os, J., Linscott, R. J., Myin-Germeys, I., Delespaul, P., & Krabbendam, L. (2009). A systematic review and meta-analysis of the psychosis continuum: Evidence for a psychosis proneness–persistence–impairment model of psychotic disorder.

Psychological Medicine, *39*(2), 179-195. https://doi.org/10.1017/S0033291708003814

Vasishth, S., Nicenboim, B., Beckman, M. E., Li, F., & Kong, E. J. (2018). Bayesian data analysis in the phonetic sciences: A tutorial introduction. *Journal of Phonetics*, *71*, 147-161. https://doi.org/10.1016/j.wocn.2018.07.008

Yap, M. J., & Balota, D. A. (2015). Visual word recognition. In: A. Pollatsek, R. Treiman (Eds.). *The Oxford handbook of reading* (pp. 26-43). Oxford University Press.
