# Supplementary material for: Individual differences in associative/semantic priming: Spreading of activation in semantic memory and epistemically unwarranted beliefs
Source: PLoS One. 2025 Feb 11;20(2):e0313239. doi: 10.1371/journal.pone.0313239 (PMC11813106; doi:10.1371/journal.pone.0313239)
Supplement: S2 File — The same document as S1 but in Spanish. (PDF) [file pone.0313239.s002.pdf]

Individual differences in associative/semantic priming: Spreading of activation in semantic memory and epistemically unwarranted beliefs – SUPPLEMENTARY MATERIAL: Data analysis details (Spanish version)

Daniel Huete-Pérez[1], Robert Davies[2], Javier Rodríguez-Ferreiro[3], and Pilar Ferré[1]

[1] Universitat Rovira i Virgili, Department of Psychology, Research Center for Behavior Assessment (CRAMC), Tarragona, Spain.

[2] Department of Psychology, Lancaster University, Bailrigg, Lancaster, United Kingdom.

[3] Grup de Recerca en Cognició i Llenguatge (GRECIL), Departament de Cognició, Desenvolupament i Psicologia de la Educació, Secció de Processos Cognitius, Institut de Neurociències (INUB), Universitat de Barcelona (UB), Barcelona, Spain.

Abstract: Starting from the *enhanced spreading of activation through semantic memory* (one of the explanatory mechanisms attempting to explain some manifestations observed in schizophrenia) and the *psychosis continuum* (a dimensional approach to psychotic disorders, where ‘normality’ and ‘psychopathology’ are not qualitatively different in nature but placed on varying levels of the same continuum), the main aim of the present research was to explore whether there are individual differences in associative/semantic priming in people with different levels of epistemically unwarranted beliefs (EUB). Participants varying in paranormal, pseudoscientific and conspiracy endorsement completed a primed lexical decision task containing related prime-target words (e.g., bulb-light) and unrelated prime-target words (e.g., sock-light). Bayesian linear mixed-effects models over response times (RTs) revealed a main direct priming effect (faster RTs in related pairs than in unrelated ones), a main facilitatory effect for some EUB scores (i.e., the higher the value for EUB score, the faster RTs), and an interactive effect between the experimental manipulation and some EUB scores (the higher the EUB score, the smaller the direct priming effect). These

results are consistent with predictions made from the enhanced spreading of activation explanatory mechanism, but other alternative accounts are also discussed.

Keywords: associative priming, semantic priming, paranormal beliefs, pseudoscientific beliefs, conspiracy beliefs.

1. Construcción/Selección de modelos

Los datos se analizaron con modelos lineales de efectos mixtos (LMEMs), los cuales tienen múltiples ventajas sobre análisis más tradicionales como ANOVAs y regresiones lineales por sujetos y por ítems (véase Baayen et al., 2008; Brown, 2021; Liben-Nowell et al., 2019). La construcción/selección de los modelos se realizó en dos pasos. En primer lugar, especificamos la estructura de efectos fijos basándonos en nuestras suposiciones teóricas. Los predictores críticos se determinaron según nuestros objetivos y predicciones, mientras que los predictores de control se determinaron en base a la identificación en la literatura de posibles variables de confusión. En segundo lugar, intentamos utilizar la estructura máxima de efectos aleatorios justificada por el diseño experimental (Barr et al., 2013).

1.1. Estructura de efectos fijos

Partiendo del mecanismo explicativo de *propagación exacerbada de la activación* (p. ej., Kiang, 2010; Kreher et al., 2008; Kuperberg, 2010; Rodríguez-Ferreiro et al., 2020) y del *continuo de la psicosis* (p. ej., Galbraith, 2021, van Os et al., 2009; perspectiva dimensional de la psicopatología, p. ej. Avasthi et al., 2014), nuestro objetivo principal fue explorar si existen diferencias individuales en *priming* asociativo/semántico en personas con diferentes niveles de creencias epistémicamente injustificadas (EUB; Lobato et al., 2014). Con este propósito, participantes cuyos niveles de EUB se midieron con instrumentos psicométricos de autoinforme realizaron una tarea de decisión léxica (LDT) con *priming*. Nuestros predictores críticos fueron:

- **Relación *prime-target* (relacionados vs. no relacionados).** En cada ensayo de la tarea principal, los participantes realizaban una LDT sobre un estímulo *target* que podía ser una palabra española real (ensayos críticos) o una pseudopalabra (ensayos de relleno). Los tiempos de respuesta (RTs) y la precisión de respuesta (RA)[1] se

[1] Solo se analizaron los RTs (pero no la RA). Una primera razón para ello es que los efectos de *priming* se observan principalmente en los RTs. Una segunda razón es que no se esperaba que los participantes cometieran muchos errores, por lo que no se dispondría de suficiente variabilidad para estimar efectos fiables.

registraron en DMDX (Forster & Forster, 2003). Estos estímulos *target* iban precedidos brevemente de un estímulo *prime* cada uno, que siempre era una palabra española real. En los ensayos críticos, las palabras *prime-target* podían estar relacionados asociativa/semánticamente (p. ej., bombilla-luz) o no estarlo (p. ej., calcetín-luz)[2]. Esperábamos replicar el típico efecto de *priming* directo (i.e., RTs más rápidos para los pares relacionados que para los no relacionados).

- **EUB.** Se midieron seis dimensiones de EUB en cada participante utilizando PEUBI (Huete-Pérez et al., 2022) y la versión revisada de PSEUDO (Fasce et al., 2021): Supersticiones (PEUBI-S), Ocultismo y Pseudociencia (PEUBI-OP), Religión Tradicional (PEUBI-TR), Formas de Vida Extraordinarias (PEUBI-ELF), Teorías Conspirativas (PEUBI-CT), y Pseudociencia (PSEUDO-R). Siguiendo el mecanismo explicativo de la propagación exacerbada de la activación, hipotetizamos que si los creyentes en EUB generalmente experimentan una propagación más rápida/potente y de mayor alcance de la activación a través de la memoria semántica (p. ej., Kreher et al., 2008; Kuperberg, 2010) en comparación con personas con niveles bajos de EUB, se esperaría un efecto principal facilitador de EUB. Es decir, puntuaciones altas de EUB se asociarían con RTs más cortos tanto en la condición relacionada (propagación más rápida/potente en los asociados cercanos; véase Kiang, 2010) como en la condición no relacionada (propagación de mayor alcance, que resultaría en la activación de asociados remotos; véase Kiang, 2010).

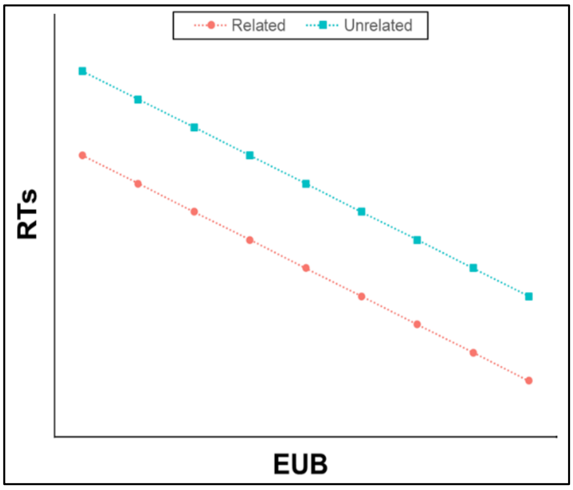

[2] En la condición relacionada, la palabra *target* era el primer asociado de la palabra *prime* según las normas de asociación libre NALC (Díez et al., 2018; Fernández et al., 2004, 2019). En la condición no- relacionada, las palabras *prime* y *target* no estaban asociadas también según NALC. Los *primes* de la condición no relacionada se seleccionaron utilizando el software Match (van Casteren & Davis, 2007) partiendo de los *primes* de la condición relacionada. Los *primes* relacionados y no relacionados no difirieron significativamente a nivel grupal en ninguna de las propiedades de las palabras consideradas, ni en sus medias (pruebas t de muestras independientes) ni en su distribución (pruebas Kolmogorov-Smirnov de dos muestras independientes).

- **Relación x EUB.** Alternativamente, si los efectos facilitadores de EUB solo se producen en una de las dos condiciones (exclusivamente en pares relacionados o en pares no relacionados), eso sugeriría que solo está ocurriendo uno de los dos mecanismos: propagación más rápida/potente en los asociados cercanos o propagación de mayor alcance activando asociados remotos. Esto se reflejaría en una interacción entre EUB y Relación.

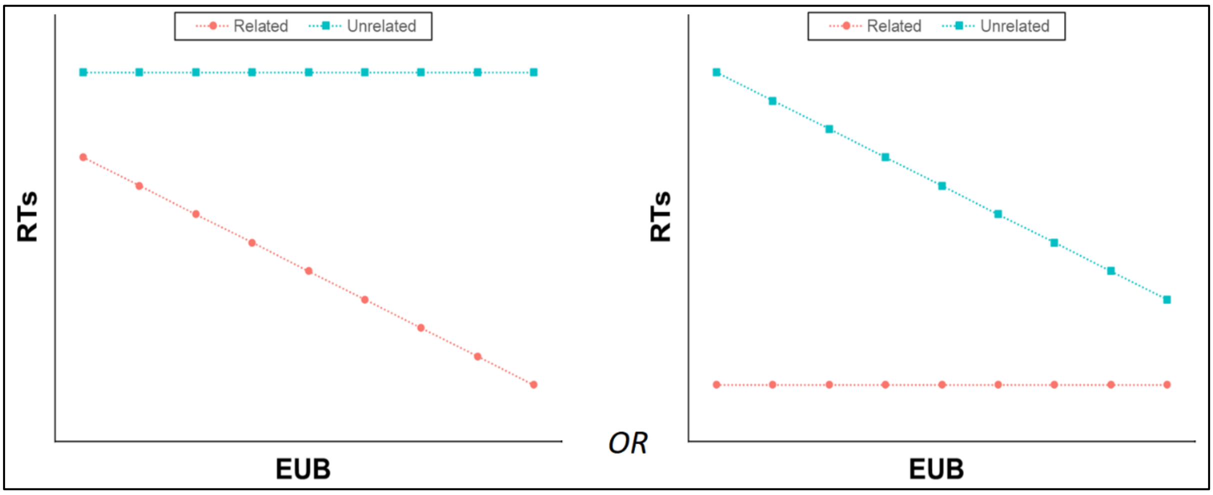

Si solo considerásemos estos predictores críticos en el análisis, estaríamos ante una seria amenaza para la validez de los resultados y las conclusiones: sabemos de otras variables que probablemente están afectando a la decisión léxica y, por ende, deberían controlarse. Por lo tanto, también se consideraron los siguientes predictores de control:

- **Propiedades de las palabras.** En la literatura psicolingüística se han identificado muchas propiedades de las palabras que afectan a la facilidad con la que se procesa/reconoce una palabra, tales como prevalencia (i.e., proporción de conocimiento en la población), frecuencia léxica (i.e., frecuencia con la que aparece en contextos reales), frecuencia subléxica (i.e., que tan común es un par/trío de letras en las palabras de una lengua determinada), longitud (i.e., cuántas letras/fonemas/sílabas tiene), vecindad léxica (i.e., número de palabras similares ortográficamente/fonológicamente), cognación (i.e., grado de similitud léxica entre traducciones), edad de adquisición (i.e., edad en la que se suele aprender), concreción (i.e., grado en que

es concreta-abstracta), familiaridad (i.e., frecuencia percibida de uso/encuentro), valencia (i.e., grado en que es emocionalmente positiva-neutra-negativa), y arousal (i.e., grado en que provoca activación/excitación interna). Para más información sobre estas variables y sus efectos, véase por ejemplo Adelman (2012), Brysbaert et al. (2016), Pexman (2012) y Yap & Balota (2015).

- **Orden de ensayo.** A medida que los participantes avanzan en el experimento, pueden mejorar su rendimiento (p. ej., aprendizaje/práctica) o empeorarlo (p. ej., fatiga). Incluir el número de ensayo en el análisis estadístico es mejor para reducir esta variable de confusión que simplemente confiar en su cancelación por aleatorización (véase Baayen et al., 2008).
- **Ensayo previo.** El rendimiento en ensayos anteriores (p. ej., RT anterior y RA anterior) afecta al rendimiento en el ensayo actual de la LDT (Baayen et al., 2008).
- **Lista.** Cada una de las palabras *target* críticas (p. ej., luz) tenía dos posibles *primes*: uno relacionado (p. ej., bombilla-luz) y otro no relacionado (p. ej., calcetín-luz). No consideramos apropiado que el mismo participante viera ambas versiones (¿se procesa el *target* de forma diferente la segunda vez? Para una discusión de los posibles efectos de repetición, véase McNamara, 2005). Por ello, creamos dos listas experimentales (A vs. B) con una versión *prime-target* en cada lista. Cada participante fue asignado aleatoriamente a una de las dos listas.
- **Relación x Lista.** Este efecto interactivo también se añadió al análisis para comprobar que la manipulación experimental (i.e., Relación) no se confunde con el método de contrabalanceo (i.e., efectos de Relación distintos en cada lista experimental).

1.2. Estructura de efectos aleatorios

Las observaciones de RTs y RA producidas en este estudio no son independientes. Más concretamente, las observaciones se agrupan por participante (i.e., las respuestas de un mismo participante están relacionadas) y por palabra *target* (i.e., las respuestas a un mismo

target están relacionadas)[3]. Incluir esta información en el modelo estadístico lo mejora (McElreath, 2020, Capítulo 13). En este sentido, la estructura máxima de efectos aleatorios justificada por diseño (Barr et al., 2013) incluiría:

- **Interceptos aleatorios (participantes + *targets*).** Es poco plausible suponer que todos los participantes o todas las palabras *target* tengan los mismos RTs o RA medios/basales (con independencia del estímulo al que se responda, hay personas más rápidas y/o con más tendencia a responder "sí" o "no" que otras; con independencia de la persona que responda, hay ítems cuyas características hacen que se procesen/ identifiquen más fácilmente que otros). En consecuencia, lo más adecuado es dejar que los interceptos varíen en función de estas unidades/factores de agrupación.
- **Pendientes aleatorias para Relación (participantes + *targets*).** De forma análoga a lo que ocurre con los interceptos, el efecto de un predictor no tiene por qué ser idéntico para todos los participantes o ítems. Pendientes aleatorias para Relación pueden ser estimadas tanto a través de participantes como a través de *targets*, dado que cada ejemplar de estas unidades de agrupación ha pasado por las dos condiciones de Relación (i.e., permitiendo así estimar el efecto de Relación para cada participante y para cada *target*).
- **Pendientes aleatorias para EUB (*targets*).** Las pendientes aleatorias para EUB pueden estimarse a través de *targets* (i.e., cada ejemplar de esta unidad de agrupación ha pasado por diferentes valores de EUB). Sin embargo, las pendientes aleatorias para EUB a través de participantes no son identificables (Barr et al., 2013), ya que cada participante tiene un valor único de EUB (i.e., efecto no estimable por participante).
- **Pendientes aleatorias para Relación x EUB (ninguno/a).** Las pendientes aleatorias para el efecto interactivo Relación x EUB no son identificables a través de

[3] Cabría preguntarse si las palabras *prime* podrían incluirse como otro factor de agrupación. Sin embargo, en consonancia con los estudios previos de *priming* sin repetición, los *primes* se consideran simplemente como la operacionalización de la manipulación experimental Relación (relacionados vs. no relacionados).

participantes (i.e., cada participante ha pasado por las dos condiciones de Relación, pero no tiene variabilidad en el nivel de EUB) ni a través de *targets* (i.e., aunque cada palabra *target* ha pasado por los dos niveles de Relación y por diferentes valores de EUB, dado el diseño contrabalanceado, no es plausible suponer que todas las posibles celdas de la interacción estén disponibles para cada *target*[4], por lo que este componente de la varianza puede capturarse adecuadamente).

Tal como exponen Barr et al. (2013), las pendientes aleatorias para los predictores de control[5] también podrían incluirse en cualquier LMEM, pero no son esenciales. Dado el aumento de la complejidad del modelo y sus problemas de estimación y computación asociados, seguimos la convención de considerar únicamente las de los predictores críticos.

1.3. Visión general de la fórmula del modelo

De todo lo dicho anteriormente, una visión general de la fórmula del modelo sería:

RTs ~ 1 + Propiedades de las palabras *prime* + Propiedades de las palabras *target* + Orden de ensayo + Ensayo previo + Relación + Lista + EUB + Relación:Lista + Relación:EUB + (1 + Relación | Participante) + (1 + Relación + EUB | Target)

Las siguientes propiedades de las palabras fueron consideradas: edad de adquisición, concreción, familiaridad, valencia, arousal, distancia normalizada de Levenshtein entre las traducciones español-catalán, prevalencia, dos variables de frecuencia léxica (frecuencia de palabra, diversidad contextual), número de letras, tres variables de vecindad léxica (número de vecinos ortográficos, número de vecinos ortográficos de mayor frecuencia, distancia media de Levenshtein para las 20 palabras más cercanas), y dos variables de frecuencia subléxica (frecuencia bigrámica, frecuencia trigrámica). Aunque todas ellas podrían incluirse

[4] Como ejemplo ilustrativo, para una puntuación PEUBI-S de 10 la palabra *target* “abuelo” solo tiene datos para la condición relacionada (lista A): no hay participantes con ese valor específico de PEUBI-S en la lista B (donde “abuelo” está en la condición no relacionada).

[5] Más concretamente, las siguientes pendientes aleatorias para predictores de control estarían justificadas por el diseño de este estudio: propiedades de las palabras *prime* (participantes), propiedades de las palabras *target* (participantes), orden de ensayo (participantes + *targets*), ensayo previo (participantes + *targets*), lista (*targets*).

en el análisis, se esperaba que los *proxies* de un mismo constructo fueran colineales[6] (i.e., las dos variables de frecuencia léxica, las tres variables de vecindad léxica, y las dos variables de frecuencia subléxica). Por ende, se realizó una eliminación preventiva de variables: solo se incluyó una variable de cada uno de estos grupos en los análisis. Seleccionamos frecuencia de palabra como medida de frecuencia léxica, número de vecinos ortográficos como medida de vecindad léxica, y frecuencia bigrámica como medida de frecuencia subléxica.

En cuanto al ensayo previo, teníamos dos variables distintas para operacionalizar sus efectos: RT previo y RA previa.

Realizamos un análisis separado con cada posible puntuación de EUB por sí sola (PEUBI-S, PEUBI-OP, PEUBI-TR, PEUBI-ELF, PEUBI-CT, PSEUDO-R): el mismo análisis se repitió seis veces, variando la puntuación de EUB en cada caso. En la medida en que las puntuaciones de EUB tienden a estar correlacionadas (p. ej., Huete-Pérez et al., 2022), la amenaza de colinealidad nos impidió incluirlas todas en un único análisis. Además, la extracción de una única puntuación por participante mediante técnicas de suma total o de reducción de dimensiones (p. ej., PCA) no se consideró apropiada ya que se enmascararían diferentes perfiles: dos individuos con la misma puntuación global podrían ser muy diferentes en sus EUB (p. ej., persona A alta superstición y baja conspiración, persona B baja superstición y alta conspiración). Por último, teniendo en cuenta que las diferentes dimensiones de EUB no necesariamente comparten los mismos mecanismos subyacentes (p. ej., Bensley, 2020; Huete-Pérez et al., 2023; Rizeq et al., 2020), consideramos importante poder explorar cada dimensión de EUB por separado.

1.4. Asunciones adicionales

- **Efectos lineales.** A pesar del debate existente sobre si los efectos de algunas

[6] Aunque la justificación teórica es suficiente para excluir algunas variables, esta cuestión se comprobó analíticamente. Más concretamente, se consideraron problemáticos los pares de propiedades de palabras con un coeficiente de correlación de Pearson de un valor absoluto ≥ .70 (Dormann et al., 2013). Cabe señalar que si un par de variables superaba el umbral solo en un tipo de palabra (≥ .70 en palabras *prime* pero no en palabras *target*, o viceversa), la variable se eliminaba tanto para los *primes* como para los *target*.

propiedades de las palabras son lineales o no lineales (p. ej., valencia; véase Hinojosa et al., 2020), en el presente análisis de datos solo se consideraron efectos lineales.

- **Sin interacciones entre propiedades de palabras.** Aunque algunos estudios han observado efectos interactivos entre algunas propiedades de las palabras (p. ej., frecuencia x imaginabilidad/concreción, González-Nosti et al., 2014), en el presente análisis de datos solo se consideraron efectos principales.

1.5. ¿Qué efectos teníamos grandes expectativas de obtener?

Para algunos de los predictores incluidos en el análisis los efectos en la literatura sobre reconocimiento de palabras son más consistentes que otros (probablemente en parte porque son efectos grandes). Más concretamente, esperábamos efectos específicos para:

- **Frecuencia de las palabras *target*.** Esperábamos replicar el típico efecto facilitador (i.e., cuanto más frecuente el *target*, más rápidos los RTs).
- **Relación.** Esperábamos replicar el típico efecto de *priming* directo (i.e., RTs más rápidos para pares *prime-target* relacionados que para no relacionados).

Dado que estos efectos están bien establecidos en la literatura, la incapacidad para obtenerlos podría sugerir una posible invalidez/falta de fiabilidad de nuestro procedimiento experimental, de los datos o del análisis estadístico.

1.6. ¿Qué pasa si obtenemos efectos nulos para los predictores de interés?

Cuando se obtienen efectos nulos para un predictor concreto, esto no implica necesariamente que los efectos no existan: podríamos no haber sido capaces de detectarlos y estimarlos con precisión. A continuación, se exponen diferentes explicaciones alternativas sobre lo que podría estar pasando si los efectos principales de EUB o los efectos interactivos Relación x EUB acabaran siendo nulos:

- **Sin efecto real (negativo verdadero).** Es posible que realmente no haya diferencias individuales por EUB (i.e., EUB no tiene efectos principales o interactivos sobre los RTs de la LDT con *priming*).

- **Efectos no lineales.** Es posible que los efectos de EUB sobre los RTs de la LDT con *priming* existan, pero de forma no lineal. En la medida que hemos considerado los efectos de EUB como lineales, no podríamos detectar dichos efectos no lineales.
- **Insuficiente potencia estadística.** Es posible que los efectos de EUB sobre los RTs de la LDT con *priming* existan, pero que sean tan pequeños que nuestro estudio no haya sido capaz de detectarlos y estimarlos con precisión.
- **Sesgo de muestreo.** Es posible que los efectos de EUB sobre los RTs de la LDT con *priming* existan, pero que la muestra de participantes o palabras no sea representativa.
- **Atenuación de relaciones: error de medida.** Es posible que los efectos de EUB sobre los RTs de la LDT con *priming* existan, pero que las medidas utilizadas no sean lo suficientemente fiables. Para informar en este sentido, se reportan las estimaciones de fiabilidad tanto para las puntuaciones de EUB (ω de McDonald y α de Cronbach) como para los RTs (método de las dos mitades).

```
### Fiabilidad con método de las dos mitades (corrección de Spearman-Brown) para los RTs tras la limpieza de datos

splithalf(data = data, outcome = "RT", score = "average", halftype = "random", permutations = 5000, var.RT = "rt", var.condition = "Relatedness", conditionlist = c("Related","Unrelated"), var.participant = "subj", average = "mean", plot = TRUE)
```

- **Atenuación de relaciones: variabilidad insuficiente.** Es posible que los efectos de EUB sobre los RTs de la LDT con *priming* existan, pero que en algunas dimensiones de EUB no haya suficiente variabilidad en la muestra (p. ej., PEUBI-TR tiende a presentar una asimetría positiva/derecha elevada porque los participantes jóvenes suelen ser principalmente no religiosos). Para informar en este sentido, se reportan los estadísticos descriptivos de las diferentes puntuaciones de EUB.
- **Variables no controladas.** Es posible que los efectos de EUB sobre los RTs de la LDT con *priming* existan, pero que estén camuflados por variables no controladas.

2. Limpieza de datos

Primero, se excluyeron del análisis de datos a los participantes con una tasa global de

error >25%. Esto se hace porque cometer muchos errores puede ser indicativo de no haber entendido la tarea, no prestar atención (incluso responder al azar), vocabulario insuficiente…

```
### Eliminar participantes con >25% de error
dataRAW = dataRAW[dataRAW$subjERR <= 25,]
```

Segundo, los ítems de relleno solo se incluyeron para tener la misma probabilidad de respuestas “sí” o “no” en la LDT, pero no son de interés. En consecuencia, solo se incluyeron en el análisis los datos de los ítems críticos.

```
### Solo los ítems críticos (i.e., palabra española real como target)
dataRAW = dataRAW[dataRAW$desc == "critical_trials",]
```

Tercero, se excluyeron del análisis los ítems con una tasa global de error >70%. Esto se hace porque una tasa de error tan alta implicaría que la palabra *target* generalmente no es conocida por la muestra de participantes.

```
### Eliminar ítems con >70% de error
dataRAW = dataRAW[dataRAW$itemERR <= 70,]
```

Cuarto, se excluyeron del análisis las observaciones con problemas de presentación (p. ej., *prime* de un ítem concreto presentado durante más de los 200 ms establecidos para un participante concreto). En la medida que DMDX registra cuando un ensayo tiene errores de presentación, estas observaciones pudieron ser identificadas.

```
### Eliminar ensayos con problemas de presentación
dataRAW = dataRAW[dataRAW$display_error == "no",]
```

Quinto, solo se incluyeron en el análisis los RTs de respuestas correctas. Si asumimos que el RT de una respuesta correcta a una palabra real representa, en parte, el tiempo necesario para procesarla y acceder a ella en el lexicón del participante (p. ej., Libben, 2008), una respuesta incorrecta podría implicar diferentes situaciones que no son de interés (p. ej., respuesta motriz involuntaria o impulsiva adivinatoria antes de procesar/activar/acceder completamente la palabra, palabra no representada en el lexicón…).

```
### Solo los RTs de respuestas correctas
dataRAW = dataRAW[dataRAW$error == 0,]
```

Sexto, se aplicaron umbrales absolutos: se eliminaron aquellos RTs más rápidos a 300 ms y aquellos que alcanzaron el límite de 2000 ms. Aunque el umbral inferior varía según el estudio (i.e., entre 200 y 300 ms), estos RTs tan rápidos tradicionalmente se han eliminado al ser interpretados como respuestas motrices involuntarias o impulsivas adivinatorias antes de procesar/activar/acceder completamente la palabra (p. ej., “fast guess” en Ratcliff & Hendrickson, 2021). Los RTs de 2000 ms se eliminaron porque no son respuestas reales de los participantes, sino el software finalizando automáticamente el ensayo por haber alcanzado el límite de tiempo para responder.

```
### Umbrales absolutos: eliminar los RTs inferiores a 300 ms o que hayan alcanzado el límite de tiempo para responder (2000 ms)

dataRAW = dataRAW[dataRAW$rt >= 300 & dataRAW$rt < 2000,]
```

Séptimo, se aplicaron umbrales relativos: se eliminaron aquellos RTs que excedían 2,5 desviaciones típicas de la media de cada participante. Aunque el umbral relativo varía según el estudio (i.e., entre 2 y 3 DT), estos RTs extremos tradicionalmente se han eliminado al ser interpretados como valores atípicos.

```
### Umbrales relativos: eliminar los RTs que excedan ±2.5 DT de la media de cada participante

dataRAW = as.data.frame(dataRAW)

data = perSubjectTrim.fnc(dataRAW, "rt", "subj", trim = 2.5)$data
```

3. Ajuste de variables

Primero, debe destacarse que los análisis se realizaron sobre los RTs brutos. Somos conscientes que, dada la asimetría positiva/derecha de la distribución de los RTs, a veces se trasforman intentando normalizar la distribución (p. ej., transformaciones inversa y logarítmica). Sin embargo, existe la posibilidad de obtener resultados espurios (tanto falsos positivos como falsos negativos) en análisis sobre RTs transformados no linealmente (véase Lo & Andrews, 2015; Schramm & Rouder, 2019). Aunque los LMEMs parecen ser robustos a las violaciones de los supuestos distribucionales (véase Schielzeth et al., 2020), una solución mejor a transformar los RTs es analizarlos utilizando una distribución de referencia

más parecida a la distribución típicamente sesgada a la derecha de los RTs, como las distribuciones Ex-Gaussiana y Gaussiana Inversa (p. ej., Heathcote et al., 1991; Lo & Andrews, 2015; véase también Lindeløv, 2019).

Segundo, para evitar que las variables categóricas se interpreten erróneamente como numéricas en RStudio, aplicamos la función `as.factor()` a todas las variables categóricas.

```
### Delimitar las variables categóricas (aunque no se usen en el análisis)
data$subj = as.factor(data$subj)
data$desc = as.factor(data$desc)
data$itemN = as.factor(data$itemN)
data$error = as.factor(data$error)
data$display_error = as.factor(data$display_error)
data$prevERR = as.factor(data$prevERR)
data$prevYES = as.factor(data$prevYES)
data$ID = as.factor(data$ID)
data$Prime = as.factor(data$Prime)
data$Target = as.factor(data$Target)
data$Relatedness = as.factor(data$Relatedness)
data$List = as.factor(data$List)
data$Sex = as.factor(data$Sex)

str(data) #Comprobar el tipo de cada variable
```

Tercero, codificamos mediante *sum-coding* aquellas variables categóricas que se iban a utilizar como predictores en el modelo (para más información sobre las alternativas de codificación de variables categóricas y sus implicaciones, véase Brehm & Alday, 2022).

```
### Codificación [-1, +1] para los dos niveles de variables categóricas que
se incluirán como predictores de efectos fijos
contrasts(data$prevERR) = contr.sum(levels(data$prevERR))
contrasts(data$Relatedness) = contr.sum(levels(data$Relatedness))
contrasts(data$List) = contr.sum(levels(data$List))

contrasts(data$prevERR) # -1 = yes, +1 = no
contrasts(data$Relatedness) # -1 = Unrelated, +1 = Related
contrasts(data$List) # -1 = B, +1 = A
```

Cuarto, transformamos las frecuencias subléxicas a una escala logarítmica con base 10 ($\log_{10}$). La relación entre los RTs y las frecuencias léxicas y subléxicas parecen seguir una escala logarítmica (p. ej., véase Adelman, 2012; Massaro et al., 1980, Capítulo 5). Aunque las medidas de frecuencia léxica ya se obtuvieron transformadas en escala $\log_{10}$, las medidas de frecuencia subléxica se obtuvieron en valores brutos.

```
### Transformación log10 de las frecuencias bigrámicas y trigrámicas

data$P_abs_tok_MBOF = log10(data$P_abs_tok_MBOF)

data$P_abs_tok_MTOF = log10(data$P_abs_tok_MTOF)

data$T_abs_tok_MBOF = log10(data$T_abs_tok_MBOF)

data$T_abs_tok_MTOF = log10(data$T_abs_tok_MTOF)
```

Quinto, estandarizamos todas las variables continuas tanto para facilitar la interpretación (i.e., todos los predictores continuos en la misma escala: *M* = 0 y *DT* = 1) como para reducir la colinealidad no esencial debido a la escala de los términos interactivos con sus componentes de orden inferior (Cohen et al., 2003, Capítulo 6).

```
### Estandarizar todas las variables continuas (aunque no se usen en el análisis), excepto la VD (i.e., RTs)

data$trial = standardize(data$trial)

data$prevRT_auto = standardize(data$prevRT_auto)

data$prevRT_man = standardize(data$prevRT_man)

data$itemERR = standardize(data$itemERR)

data$subjERR = standardize(data$subjERR)

data$FSG = standardize(data$FSG)

data$Age = standardize(data$Age)

data$PEUBI_S = standardize(data$PEUBI_S)

data$PEUBI_OP = standardize(data$PEUBI_OP)

data$PEUBI_TR = standardize(data$PEUBI_TR)

data$PEUBI_ELF = standardize(data$PEUBI_ELF)

data$PEUBI_CT = standardize(data$PEUBI_CT)

data$PSEUDO_R = standardize(data$PSEUDO_R)

data$P_AoA = standardize(data$P_AoA)

data$P_Conc = standardize(data$P_Conc)

data$P_Fam = standardize(data$P_Fam)
```

```
data$P_Val = standardize(data$P_Val)
data$P_Aro = standardize(data$P_Aro)
data$P_NLD_Spanish_Catalan = standardize(data$P_NLD_Spanish_Catalan)
data$P_prevalence_nts = standardize(data$P_prevalence_nts)
data$P_log_frq = standardize(data$P_log_frq)
data$P_num_letters = standardize(data$P_num_letters)
data$P_N = standardize(data$P_N)
data$P_NHF = standardize(data$P_NHF)
data$P_Lev_N = standardize(data$P_Lev_N)
data$P_abs_tok_MBOF = standardize(data$P_abs_tok_MBOF)
data$P_abs_tok_MTOF = standardize(data$P_abs_tok_MTOF)
data$P_log_Ctx_div = standardize(data$P_log_Ctx_div)
data$T_AoA = standardize(data$T_AoA)
data$T_Conc = standardize(data$T_Conc)
data$T_Fam = standardize(data$T_Fam)
data$T_Val = standardize(data$T_Val)
data$T_Aro = standardize(data$T_Aro)
data$T_NLD_Spanish_Catalan = standardize(data$T_NLD_Spanish_Catalan)
data$T_prevalence_nts = standardize(data$T_prevalence_nts)
data$T_log_frq = standardize(data$T_log_frq)
data$T_num_letters = standardize(data$T_num_letters)
data$T_N = standardize(data$T_N)
data$T_NHF = standardize(data$T_NHF)
data$T_Lev_N = standardize(data$T_Lev_N)
data$T_abs_tok_MBOF = standardize(data$T_abs_tok_MBOF)
data$T_abs_tok_MTOF = standardize(data$T_abs_tok_MTOF)
data$T_log_Ctx_div = standardize(data$T_log_Ctx_div)

describe(data) #Comprobar la estandarización
```

4. Especificaciones para los LMEMs frecuentistas

4.1. Fórmula del modelo en el paquete *lme4* + Estructura de efectos aleatorios

Cuando se utiliza el paquete *lme4* (Bates et al., 2015), los problemas de convergencia y singularidad son bastante comunes para estructuras de efectos aleatorios complejas

(Meteyard & Davies, 2020). En muchos casos, esto impide utilizar la estructura máxima de efectos aleatorios justificada por diseño (Barr et al., 2013). Cabe señalar que la forma de abordar estos problemas aún no está estandarizada (Bates et al., 2015; Meteyard & Davies, 2020). En este estudio, si un modelo máximo producía problemas de convergencia o singularidad, la estructura máxima se simplificó iterativamente eliminando el efecto aleatorio con menor varianza (hasta que no haya problemas de convergencia o singularidad)[7].

```
### Determinar la estructura de efectos aleatorios que no produce problemas
empezando por la máxima justificada por diseño (Barr et al., 2013)
siguiendo parcialmente a Bates et al. (2018)

Model = lmer(rt ~ 1 + P_AoA + P_Conc + P_Fam + P_Val + P_Aro +
P_NLD_Spanish_Catalan + P_prevalence_nts + P_log_frq + P_num_letters + P_N
+ P_abs_tok_MBOF + T_AoA + T_Conc + T_Fam + T_Val + T_Aro +
T_NLD_Spanish_Catalan + T_prevalence_nts + T_log_frq + T_num_letters + T_N
+ T_abs_tok_MBOF + trial + prevRT_man + prevERR + Relatedness + List + EUB
+ Relatedness:List + Relatedness:EUB + (1 + Relatedness | subj) + (1 +
Relatedness + EUB | Target), data = data))

# ¿Problemas? Repite el modelo sin el efecto aleatorio de menor varianza
```

Como se ha dicho en la sección anterior, los análisis se realizaron sobre los RTs brutos. Se utilizó la distribución de referencia por defecto de `lmer()` (i.e., Gaussiana).

4.2. Comprobaciones del modelo

Los supuestos estadísticos de los LMEMs se comprobaron con el paquete *performance* (Lüdecke et al., 2021): ausencia de valores atípicos, linealidad, normalidad, homocedasticidad, y ausencia de colinealidad.

```
### Comprobar los supuestos del modelo

check_outliers(Model)

check_model(Model)
```

Hay que decir que los LMEMs parecen ser robustos a las violaciones de los supuestos distribucionales (véase Schielzeth et al., 2020).

[7] Esta estrategia es una adaptación de Bates et al. (2018). Su propuesta implica seguir los siguientes pasos hasta que el modelo no produzca problemas de convergencia o singularidad. Primero, modelo máximo. Segundo, repite el modelo forzando las correlaciones entre efectos aleatorios a ser cero. Tercero, elimina del modelo el efecto aleatorio con menor varianza (repite este paso tantas veces como sea necesario). Por último, cuando el modelo ya no sea problemático, permite que los efectos aleatorios restantes estén correlacionados (si vuelve a ser problemático, deshaz este paso). En nuestro caso nos saltamos la parte de forzar que los efectos aleatorios no estén correlacionados, dado que esto no parece funcionar correctamente con predictores categóricos.

4.3. Extraer información del modelo

Siguiendo las tablas para reportar LMEMs de Meteyard & Davies (2020), se extrajo del modelo la información de las estimaciones de los efectos fijos, las estimaciones de los efectos aleatorios y el ajuste del modelo.

```
### Extraer información del modelo

summary(Model)

confint.merMod(Model, level = 0.95, method = c("Wald"), quiet = FALSE,
oldNames = FALSE) #Intervalos de confianza de los efectos fijos

r2(Model) #Proporción de varianza explicada por el modelo (R²)
```

Aunque los efectos principales son relativamente fáciles de interpretar directamente (i.e., b > 0 implica que cuanto mayor es el valor del predictor, mayores/más lentos son los RTs; b < 0 implica que cuanto mayor es el valor del predictor, menores/más rápidos son los RTs), los efectos interactivos no son tan intuitivos y es mejor representarlos gráficamente.

```
### Representar gráficamente los efectos de interés

plot(ggemmeans(Model, terms = c("EUB[-2,-1.5,-1,-0.5,0,0.5,1,1.5,2]",
"Relatedness"), ci_level = 0.95)) + ylim(450,650) #Medias marginales
estimadas y sus intervalos de confianza al 95%
```

5. Especificaciones para los LMEMs Bayesianos

Los LMEMs Bayesianos tienen varias ventajas sobre los frecuentistas, como mayor flexibilidad para especificar el modelo, cuantificación de la incertidumbre en torno a los parámetros estimados, posibilidad de incluir conocimiento previo, o evitar problemas asociados a las comparaciones múltiples (Nalborczyk et al., 2019; Vasishth et al., 2018). Ahora bien, probablemente la mayor ventaja es que, cuando se utiliza el paquete *brms* (Bürkner, 2017), los LMEMs Bayesianos no producen los problemas de convergencia o singularidad de los LMEMs frecuentistas al intentar utilizar la estructura máxima de efectos aleatorios justificada por diseño (p. ej., véase Nalborczyk et al., 2019; Vasishth et al., 2018).

5.1. Selección de la distribución de referencia para la variable dependiente/de respuesta

Como se ha dicho anteriormente, los análisis se realizaron sobre los RTs brutos (i.e.,

sin transformar). Para tener en cuenta la asimetría positiva/derecha típica de la distribución de RTs, optamos por utilizar una distribución Ex-Gaussiana, ya que parece adecuada para modelar específicamente los RTs de tareas rápidas con dos opciones de respuesta (véase Matzke & Wagenmakers, 2009). Se realizaron comprobaciones para garantizar que esta distribución era adecuada para nuestros datos (véase la subsección 5.4).

5.2. Distribuciones a priori (*priors*)

Las distribuciones débilmente informativas parecen adecuadas para especificar las *priors* de los parámetros del modelo: dan un poco de información al descartar valores extremos muy improbables (contribuyendo así a la regularización del modelo, es decir, a evitar el sobreajuste), pero siguen permitiendo un amplio rango de valores (lo que permite que los datos influyan en gran medida al modelo, evitando así el infraajuste) (véase McElreath, 2020, Capítulo 7; Stan Development Team, 2023; Vasishth et al., 2018).

```
### Distribuciones a priori personalizadas
custom_priors = c(set_prior("normal(700, 200)", class = "Intercept"),
                  set_prior("normal(0, 50)",    class = "b"),
                  set_prior("normal(0, 50)",    class = "sd"),
                  set_prior("lkj(2)",           class = "cor"))
```

- **Intercepto ~ Normal(700, 200).** Con esta *prior* consideramos que el 95% de los valores para las estimaciones de interceptos se situarán entre [300, 1100] ms, dado que el 95% de los valores de una distribución normal están entre ±2 *DT* de la *M*. El límite inferior de 300 ms se determinó a partir de nuestros umbrales absolutos (véase sección 2). El límite superior de 1100 ms se determinó por nuestra experiencia previa con la LDT (i.e., es poco probable que los RTs medios sean superiores a 1 s).[8]

```
### Visualizar la distribución N(700,200)
library(BayesTools)
```

[8] Una *prior* débilmente informativa podría haber sido Normal (1150, 425), dado que el rango completo de valores posible dado nuestros umbrales absolutos sería [300, 2000] ms. Sin embargo, en este parámetro nos permitimos ser más informativos dado nuestro conocimiento previo del fenómeno.

```
plot(prior(distribution = "normal", parameters =
list(mean = 700, sd = 200)))
```

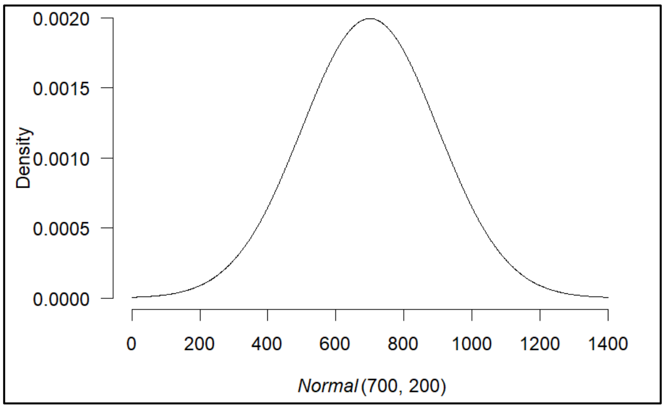

- **Pendientes ~ Normal(0, 50).** Con esta *prior* consideramos que el 95% de los valores para las estimaciones de efectos fijos se situarían entre [-100, +100] ms por unidad de cambio en el predictor. Dado la escala de los RTs, un predictor que implicara un efecto de ±100 ms por unidad sería enorme (p. ej., González-Nosti et al., 2014).

```
### Visualizar la distribución N(0,50)

library(BayesTools)

plot(prior(distribution = "normal", parameters =
list(mean = 0, sd = 50)))
```

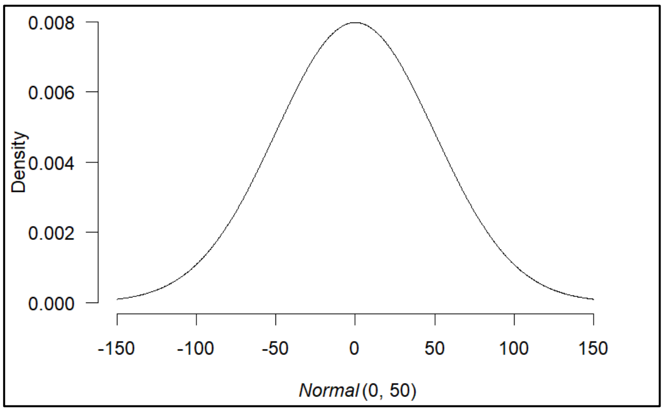

- **Desviaciones de efectos aleatorios ~ Normal$_+$(0, 50).** Con esta *prior* consideramos que el 95% de los valores para las estimaciones de las desviaciones de efectos aleatorios se situarían entre [0, +100][9]. Por lo tanto, suponemos que lo más probable es que la variabilidad de los efectos aleatorios sea cero, pero que podría ser grande (aunque será menos probable cuanto mayor sea su valor).

[9] El paquete *brms* restringe automáticamente las estimaciones de tipo `class = "sd"` solo a valores positivos. Por lo tanto, en especificar la distribución debe tenerse en cuenta que solo se permitirán valores ≥0.

```
### Visualizar la distribución N₊(0,50)

library(BayesTools)

plot(prior(distribution = "normal", parameters =
list(mean = 0, sd = 50), truncation = list(0, Inf)))
```

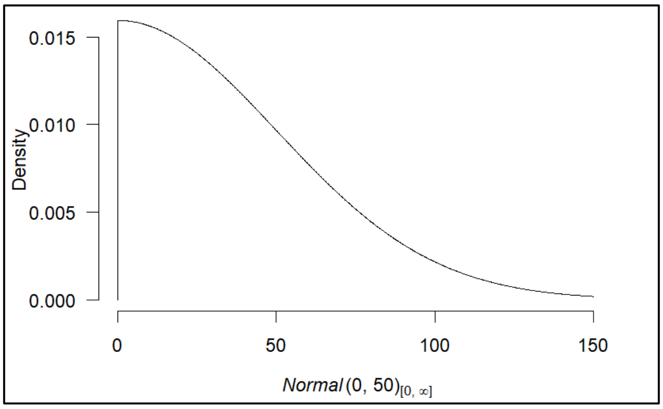

- **Correlación entre efectos aleatorios ~ LKJ(2).** La *prior* por defecto de *brms* LKJ(1) implica que todos los valores de correlación (i.e., [-1, +1]) son equiprobables (véase Bürkner, 2017). Sin embargo, siguiendo a Nalborczyk et al. (2019) y Vasishth et al. (2018), al especificar LKJ(2) consideramos muy improbables que las correlaciones entre efectos aleatorios sean perfectas (i.e., ±1,00). Esta especificación garantiza que dichas correlaciones puedan estimarse (Vasishth et al., 2018), lo cual supone una de las principales razones que subyacen a los problemas de los LMEMs frecuentistas.

```
### Visualizar la distribución LKJ(2)

library(rethinking)

dens(rlkjcorr(n = 1000000, K = 2, eta = 2)[,1,2])
```

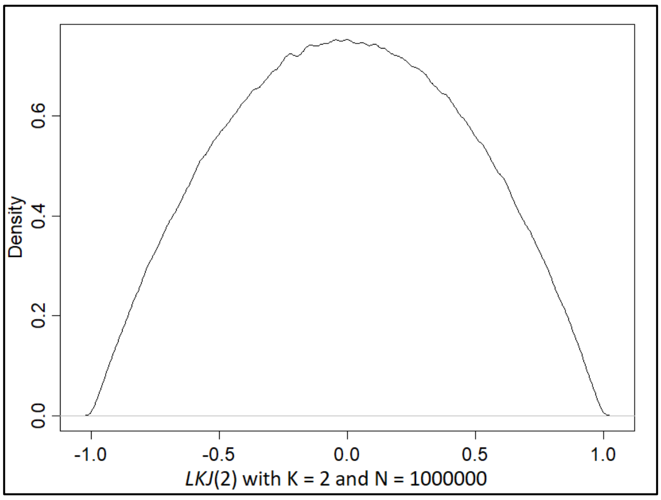

Hemos intentado justificar nuestras elecciones para las *priors*, pero otras personas podrían no estar de acuerdo con nosotros. No obstante, cabe destacar que la influencia de las *priors* disminuye a medida que aumenta el número de observaciones (p. ej., Kruschke, 2015, Capítulo 6; Smid & Winter, 2020), por lo que la elección de unas u otras *priors* no deberían suponer ninguna diferencia sustancial dada la gran cantidad de observaciones de este estudio (pero cuidado con *priors* muy informativas, véase Smeets & van de Schoot, 2019)[10].

5.3. Fórmula del modelo en el paquete *brms*

El código básico del paquete *brms* es idéntica a la del paquete *lme4*, si bien pueden ser necesarios algunos argumentos adicionales para espeficicar los LMEMs Bayesianos:

```
### Computar y guardar el modelo con la estructura máxima de efectos
aleatorios justificada por el diseño (Barr et al., 2013).

Model = brm(rt ~ 1 + P_AoA + P_Conc + P_Fam + P_Val + P_Aro +
P_NLD_Spanish_Catalan + P_prevalence_nts + P_log_frq + P_num_letters + P_N
+ P_abs_tok_MBOF + T_AoA + T_Conc + T_Fam + T_Val + T_Aro +
T_NLD_Spanish_Catalan + T_prevalence_nts + T_log_frq + T_num_letters + T_N
+ T_abs_tok_MBOF + trial + prevRT_man + prevERR + Relatedness + List + EUB
+ Relatedness:List + Relatedness:EUB + (1 + Relatedness | subj) + (1 +
Relatedness + EUB | Target), data = data, family = exgaussian(), prior =
custom_priors, iter = 10000, warmup = 2000, chains = 4, cores = 4)

saveRDS(Model, file = "Model.rds")
```

- **Family.** Seleccionamos la distribución Ex-Gaussiana como referencia para los RTs.
- **Prior.** En lugar de utilizar las *priors* por defecto de *brms*, seleccionamos las *priors* personalizadas especificadas en la subsección anterior.
- **Iter.** Establecimos en 10000 el número total de iteraciones/muestras que el procedimiento MCMC utiliza en cada cadena/muestreo independiente (por defecto es

[10] Smeets & van de Schoot (2019) muestran otra forma de visualizar las *priors*: debe añadirse el argumento `sample_priors = "yes"` a la función `brm()` (para indicarle al procedimiento MCMC que las muestras deben extraerse también de las *priors*, ya que por defecto solo extrae muestras de las distribuciones posteriores) y, a continuación, usar la función `plot(hypothesis(Model, "predictor > 0"))`. A título ilustrativo, el código para visualizar una pendiente (p. ej., efecto fijo de alguna EUB) sería `plot(hypothesis(Model, "EUB > 0", class = "b"))`, para la desviación de un efecto aleatorio (p. ej., pendiente aleatoria de Relación a través de *targets*) sería `plot(hypothesis(Model, "Target__Relatedness1 > 0", class = "sd"))`, y para la correlación entre efectos aleatorios (p. ej., pendientes aleatorias de Relación y EUB a través de *targets*) sería `plot(hypothesis(Model, "Target__Relatedness1__EUB > 0", class = "cor"))`. Sin embargo, esta forma de visualizar las *priors* tiene al menos dos desventajas. En primer lugar, en nuestra experiencia, el argumento `sample_priors = "yes"` aumenta el tiempo necesario para realizar el procedimiento MCMC. En segundo lugar, la *prior* para el intercepto no puede ser extraída por razones técnicas (véase la documentación de *brms*) y, por lo tanto, no estará disponible para ser visualizada.

2000)[11]. Este valor suma (1) el número de iteraciones/muestras utilizadas para la fase de calentamiento y (2) el número de iteraciones/muestras utilizadas para la fase de aproximación de la distribución posterior (McElreath, 2020, Capítulo 9).

- **Warmup.** Establecimos en 2000 el número de iteraciones/muestras que el procedimiento MCMC utiliza para la fase de calentamiento en cada cadena/muestreo independiente (por defecto es `Iter/2`)[11]. Dado que estas iteraciones/muestras de calentamiento no se consideran para aproximar la distribución posterior (Nalborczyk et al., 2019; Vasishth et al., 2018), en este caso el número de iteraciones/muestras utilizadas para aproximar la distribución posterior en cada cadena es `Iter – Warmup = 8000`.
- **Chains.** Establecimos el número de cadenas de muestreo independiente en 4 (corresponde con el valor por defecto de *brms*)[12].
- **Cores.** Establecimos el número de núcleos de procesamiento en 4 (en lugar del valor por defecto de *brms*) para que cada cadena se procese en paralelo[13].
- **SaveRDS.** Dado que los LMEMs Bayesianos complejos pueden tardar tiempo en computarse (en este caso, aproximadamente 5 horas cada modelo), conviene guardar cada modelo como un archivo .rds, lo que permite volver a cargarlo más tarde.

5.4. Comprobaciones del modelo

Primero, comprobamos los **factores de inflación de la varianza (VIF)** para asegurar que no había grados importantes de colinealidad entre predictores.

```
### Comprobaciones del modelo
check_collinearity(Model) #VIFs (todos deben ser ≤5, y mejor si son ≤3)
```

[11] Primero se utilizó el número de iteraciones por defecto de *brms* (i.e., `iter = 2000`, `warmup = 1000`). Sin embargo, como apareció un aviso de ESS bajo, seguimos la recomendación de ejecutar más iteraciones (Stan Development Team, 2022).

[12] Dada la naturaleza estocástica del procedimiento MCMC, una sola cadena de muestreo podría comportarse de forma idiosincrática y producir resultados no fiables. En consecuencia, se recomienda utilizar >1 cadena para garantizar que los muestreos independientes convergen en la misma solución (McElreath, 2020, Capítulo 9).

[13] El número de núcleos de procesamiento varía entre ordenadores, por lo que es posible que tengas que ajustar el valor si ejecutas el código. Si quieres saber cuantos núcleos tiene tu ordenador, usa la función `detectCores()`.

Segundo, realizamos una ***posterior predictive check*** para asegurar que la distribución de referencia elegida para el modelo era adecuada para nuestros datos.

```
### Comprobaciones del modelo

pp_check(Model, ndraws = 100) #Posterior predictive check
```

Tercero, algunos diagnósticos para ver si el MCMC ha tenido problemas de convergencia y/o eficiencia (véase McElreath, 2020, Capítulo 9): ***trace and trank plots,*** diagnóstico de convergencia **R-hat ($\hat{R}$)** de Gelman-Rubin, ***effective sample size*** **(EES)**.

```
### Comprobaciones del modelo

plot(Model, combo = c("trace","rank_overlay")) #Trace and trank plots (para ver solo un parámetro a la vez, añade el argumento N = 1)

summary(Model) #R-hat (todos deben ser <1.01) + ESS (todos deben ser >100 veces el número de cadenas) --> https://mc-stan.org/misc/warnings
```

5.5. Extraer información del modelo

Aunque la información del modelo puede extraerse de forma tabulada (como en los LMEMs frecuentistas), al menos para los efectos fijos resulta recomendable representar gráficamente las distribuciones posteriores (en los LMEMs Bayesianos el énfasis está en toda la distribución de valores posibles, no solo en estimaciones puntuales de tendencia central).

```
### Extraer información del modelo

summary(Model)

plot(Model, combo = c("dens","hist")) #Distribuciones posteriores de los parámetros (para ver solo una a la vez, añade el argumento N = 1)

plot(eti(Model, effects = "fixed", ci = 0.95)) + xlim(-40,40) #Distribución posterior de cada efecto fijo con su ICr 95% (Equally-Tailed Intervals)
```

De igual modo que en los LMEMs frecuentistas, se representaron gráficamente los efectos interactivos para comprenderlos mejor.[14]

```
### Representar gráficamente los efectos de interés

plot(ggemmeans(Model, terms = c("EUB[-2,-1.5,-1,-0.5,0,0.5,1,1.5,2]", "Relatedness"), ci_level = 0.95)) + ylim(475,600) #Medianas marginales estimadas y sus intervalos de credibilidad al 95% (Highest Posterior Density Interval)
```

[14] En este caso, aunque el código es el mismo que en los LMEMs frecuentistas, con los LMEMs Bayesianos las estimaciones puntuales son medianas (en lugar de medias) y el argumento CI produce intervalos de credibilidad (en lugar de intervalos de confianza).

6. Referencias

Adelman, J. S. (2012). Methodological issues with words. In: J. S. Adelman (Ed.) *Visual word recognition (vol. 1): Models and methods, orthography and phonology* (pp. 116-138). Psychology Press.

Avasthi, A., Sarkar, S., & Grover, S. (2014). Approaches to psychiatric nosology: A viewpoint. *Indian Journal of Psychiatry*, *56*(3), 301-304. https://doi.org/10.4103/0019-5545.120560

Baayen, R. H., Davidson, D. J., & Bates, D. M. (2008). Mixed-effects modeling with crossed random effects for subjects and items. *Journal of Memory and Language*, *59*(4), 390-412. https://doi.org/10.1016/j.jml.2007.12.005

Barr, D. J., Levy, R., Scheepers, C., & Tily, H. J. (2013). Random effects structure for confirmatory hypothesis testing: Keep it maximal. *Journal of Memory and Language*, *68*(3), 255-278. https://doi.org/10.1016/j.jml.2012.11.001

Bates, D., Kliegl, R., Vasishth, S., Baayen, R. H. (2018). *Parsimonious mixed models.* arXiv. https://doi.org/10.48550/arXiv.1506.04967

Bates, D., Mächler, M., Bolker, B. M., Walker, S. C. (2015). Fitting linear mixed-effects models using lme4. *Journal of Statistical Software*, *67*(1), 1–48. https://doi.org/10.18637/jss.v067.i01

Bensley, D. A., Lilienfeld, S. O., Rowan, K. A., Masciocchi, C. M., & Grain, F. (2020). The generality of belief in unsubstantiated claims. *Applied Cognitive Psychology*, *34*(1), 16-28. https://doi.org/10.1002/acp.3581

Brehm, L., & Alday, P. M. (2022). Contrast coding choices in a decade of mixed models. *Journal of Memory and Language*, *125*, 104334. https://doi.org/10.1016/j.jml.2022.104334

Brown, V. A. (2021). An introduction to linear mixed-effects modeling in R. *Advances in Methods and Practices in Psychological Science*, *4*(1), 2515245920960351. https://doi.org/10.1177/2515245920960351

Brysbaert, M., Stevens, M., Mandera, P., & Keuleers, E. (2016). The impact of word prevalence on lexical decision times: Evidence from the Dutch Lexicon Project 2. *Journal of Experimental Psychology: Human Perception and Performance*, *42*(3), 441–458. https://doi.org/10.1037/xhp0000159

Bürkner, P. C. (2017). brms: An R package for Bayesian multilevel models using Stan. *Journal of Statistical Software*, *80*(1), 1–28. https://doi.org/10.18637/jss.v080.i01

Cohen, J., Cohen, P., West, S. G., & Aiken, L. S. (2003). *Applied multiple regression/correlation analysis for the behavioral sciences* (3rd ed.). Routledge.

Díez, E., Alonso, M. A., Rodríguez, N., & Fernández, A. (2018). *Free-association norms for a large set of words in Spanish.* Unpublished. https://doi.org/10.13140/RG.2.2.17703.70560

Dormann, C. F., Elith, J., Bacher, S., Buchmann, C., Carl, G., Carré, G., García-Márquez, J. R., Gruber, B., Lafourcade, B., Leitão, P. J., Münkemüller, T., McClean, C., Osborne, P. E., Reineking, B., Schröder, B., Skidmore, A. K., Zurell, D., & Lautenbach, S. (2013). Collinearity: a review of methods to deal with it and a simulation study evaluating their performance. *Ecography*, *36*(1), 27-46. https://doi.org/10.1111/j.1600-0587.2012.07348.x

Fasce, A., Avendaño, D., & Adrián-Ventura, J. (2021). Revised and short versions of the pseudoscientific belief scale. *Applied Cognitive Psychology*, *35*(3), 828-832. https://doi.org/10.1002/acp.3811

Fernández, A., Díez, E., & Alonso, M. A. (2019). *Normas de Asociación Libre en Castellano (NALC) de la Universidad de Salamanca* [Online database]. Retrieved February 17, 2023, from http://campus.usal.es/gimc/nalc

Fernández, A., Díez, E., Alonso, M. A., & Beato, M. S. (2004). Free-association norms for the Spanish names of the Snodgrass and Vanderwart pictures. *Behavior Research Methods, Instruments, & Computers*, *36*, 577-583. https://doi.org/10.3758/BF03195604

Forster K. I., & Forster, J. C. (2003). DMDX: A Windows display program with millisecond accuracy. *Behavior Research Methods, Instruments, & Computers, 35*(1), 116–124. https://doi.org/10.3758/BF03195503

Galbraith, N. (2021). Delusions and pathologies of belief: Making sense of conspiracy beliefs via the psychosis continuum. In V. Cardella & A. Gangemi (Eds.), *Psychopathology and Philosophy of Mind* (pp. 117-144). Routledge. https://doi.org/10.4324/9781003009856-8

González-Nosti, M., Barbón, A., Rodríguez-Ferreiro, J., & Cuetos, F. (2014). Effects of the psycholinguistic variables on the lexical decision task in Spanish: A study with 2,765 words. *Behavior Research Methods, 46*, 517-525. https://doi.org/10.3758/s13428-013-0383-5

Heathcote, A., Popiel, S. J., & Mewhort, D. J. (1991). Analysis of response time distributions: An example using the Stroop task. *Psychological Bulletin, 109*(2), 340-347. https://doi.org/10.1037/0033-2909.109.2.340

Hinojosa, J. A., Moreno, E. M., & Ferré, P. (2020). Affective neurolinguistics: towards a framework for reconciling language and emotion. *Language, Cognition and Neuroscience, 35*(7), 813-839. https://doi.org/10.1080/23273798.2019.1620957

Huete-Pérez, D., & Ferré, P. (2023). Individual differences in visual word recognition: the role of epistemically unwarranted beliefs on affective processing and signal detection. *Language and Cognition, 15*(2), 314-336. https://doi.org/10.1017/langcog.2022.38

Huete-Pérez, D., Morales-Vives, F., Gavilán, J. M., Boada, R., & Haro, J. (2022). Popular Epistemically Unwarranted Beliefs Inventory (PEUBI): A psychometric instrument for assessing paranormal, pseudoscientific and conspiracy beliefs. *Applied Cognitive Psychology, 36*(6), 1260-1276. https://doi.org/10.1002/acp.4010

Kiang, M. (2010). Schizotypy and language: A review. *Journal of Neurolinguistics, 23*(3), 193-203. https://doi.org/10.1016/j.jneuroling.2009.03.002

Kreher, D. A., Holcomb, P. J., Goff, D., & Kuperberg, G. R. (2008). Neural evidence for faster and further automatic spreading activation in schizophrenic thought disorder. *Schizophrenia Bulletin*, *34*(3), 473-482. https://doi.org/10.1093/schbul/sbm108

Kruschke, J. (2015). *Doing Bayesian data analysis: A tutorial with R, JAGS, and Stan* (2nd ed.). Academic Press.

Kuperberg, G. R. (2010). Language in schizophrenia part 1: an introduction. *Language and Linguistics Compass*, *4*(8), 576-589. https://doi.org/10.1111/j.1749-818X.2010.00216.x

Libben, G. (2008). Disorders of lexis. In B. Stemmer & H. A. Whitaker (Eds.), *Handbook of the Neuroscience of Language* (pp. 147-154). Elsevier. https://doi.org/10.1016/B978-0-08-045352-1.00014-8

Liben-Nowell, D., Strand, J., Sharp, A., Wexler, T., & Woods, K. (2019). The danger of testing by selecting controlled subsets, with applications to spoken-word recognition. *Journal of Cognition*, *2*(1), 2. https://doi.org/10.5334%2Fjoc.51

Lindeløv, J. K. (2019). *Reaction time distributions: An interactive overview*. https://lindeloev.github.io/shiny-rt/

Lo, S., & Andrews, S. (2015). To transform or not to transform: Using generalized linear mixed models to analyse reaction time data. *Frontiers in Psychology*, *6*, 1171. https://doi.org/10.3389/fpsyg.2015.01171

Lobato, E., Mendoza, J., Sims, V., & Chin, M. (2014). Examining the relationship between conspiracy theories, paranormal beliefs, and pseudoscience acceptance among a university population. *Applied Cognitive Psychology*, *28*(5), 617–625. https://doi.org/10.1002/acp.3042

Lüdecke, D., Ben-Shachar, M. S., Patil, I., Waggoner, P., Makowski, D., (2021). performance: An R package for assessment, comparison and testing of statistical models. *Journal of Open Source Software*, *6*(60), 3139. https://doi.org/10.21105/joss.03139

Massaro, D. W., Taylor, G. A., Venezky, R. L., Jastrzembski, J. E., Lucas, P. A. (1980). *Letter and word perception: Orthographic structure and visual processing in reading*. North-Holland.

Matzke, D., & Wagenmakers, E. J. (2009). Psychological interpretation of the ex-Gaussian and shifted Wald parameters: A diffusion model analysis. *Psychonomic Bulletin & Review, 16*, 798-817. https://doi.org/10.3758/PBR.16.5.798

McElreath, R. (2020). *Statistical rethinking: A Bayesian course with examples in R and STAN* (2nd Ed.). Champan and Hall/CRC Press. https://doi.org/10.1201/9780429029608

McNamara, T. P. (2005). *Semantic priming: Perspectives from memory and word recognition*. Psychology Press.

Meteyard, L., & Davies, R. A. I. (2020). Best practice guidance for linear mixed-effects models in psychological science. *Journal of Memory and Language, 112,* 104092. https://doi.org/10.1016/j.jml.2020.104092

Nalborczyk, L., Batailler, C., Lœvenbruck, H., Vilain, A., & Bürkner, P. C. (2019). An introduction to Bayesian multilevel models using brms: A case study of gender effects on vowel variability in standard Indonesian. *Journal of Speech, Language, and Hearing Research, 62*(5), 1225-1242. https://doi.org/10.1044/2018_JSLHR-S-18-0006

Pexman, P. M. (2012). Meaning-based influences on visual word recognition. In: J. S. Adelman (Ed.) *Visual word recognition (vol. 2): Meaning and context, individuals and development* (pp. 24-43). Psychology Press.

Ratcliff, R., & Hendrickson, A. T. (2021). Do data from mechanical Turk subjects replicate accuracy, response time, and diffusion modeling results? *Behavior Research Methods, 53*(6), 2302-2325. https://doi.org/10.3758/s13428-021-01573-x

Rizeq, J., Flora, D. B., & Toplak, M. E. (2020). An examination of the underlying dimensional structure of three domains of contaminated mindware: Paranormal

beliefs, conspiracy beliefs, and anti-science attitudes. *Thinking & Reasoning*, *27*(2), 187–211. https://doi.org/10.1080/13546783.2020.1759688

Rodríguez-Ferreiro, J., Aguilera, M., & Davies, R. (2020). Semantic priming and schizotypal personality: Reassessing the link between thought disorder and enhanced spreading of semantic activation. *PeerJ, 8*, e9511. https://doi.org/10.7717/peerj.9511

Schielzeth, H., Dingemanse, N. J., Nakagawa, S., Westneat, D. F., Allegue, H., Teplitsky, C., Réale, D., Dochtermann, N. A., Garamszegi, L. Z., Araya-Ajoy, Y. G. (2020). Robustness of linear mixed-effects models to violations of distributional assumptions. *Methods in Ecology and Evolution, 11*(9), 1141-1152. https://doi.org/10.1111/2041-210X.13434

Schramm, P., & Rouder, J. N. (2019). *Are reaction time transformations really beneficial?* https://doi.org/10.31234/osf.io/9ksa6

Smeets, L., & van de Schoot, R. (2019). *Influence of priors: Popularity data.* https://www.rensvandeschoot.com/tutorials/brms-priors/

Smid, S. C., & Winter, S. D. (2020). Dangers of the defaults: A tutorial on the impact of default priors when using Bayesian SEM with small samples. *Frontiers in Psychology*, *11*, 611963. https://doi.org/10.3389/fpsyg.2020.611963

Stan Development Team (2022). *Runtime warnings and convergence problems.* https://mc-stan.org/misc/warnings.html

Stan Development Team (2023). *Prior choice recommendations.* https://github.com/stan-dev/stan/wiki/Prior-Choice-Recommendations

van Casteren, M., & Davis, M. H. (2007). Match: A program to assist in matching the conditions of factorial experiments. *Behavior Research Methods, 39*(4), 973–978. https://doi.org/10.3758/BF03192992

van Os, J., Linscott, R. J., Myin-Germeys, I., Delespaul, P., & Krabbendam, L. (2009). A systematic review and meta-analysis of the psychosis continuum: Evidence for a psychosis proneness–persistence–impairment model of psychotic disorder.

Psychological Medicine, *39*(2), 179-195. https://doi.org/10.1017/S0033291708003814

Vasishth, S., Nicenboim, B., Beckman, M. E., Li, F., & Kong, E. J. (2018). Bayesian data analysis in the phonetic sciences: A tutorial introduction. *Journal of Phonetics*, *71*, 147-161. https://doi.org/10.1016/j.wocn.2018.07.008

Yap, M. J., & Balota, D. A. (2015). Visual word recognition. In: A. Pollatsek, R. Treiman (Eds.). *The Oxford handbook of reading* (pp. 26-43). Oxford University Press.
