# Supplementary material for: Individual differences in associative/semantic priming: Spreading of activation in semantic memory and epistemically unwarranted beliefs
Source: PLoS One. 2025 Feb 11;20(2):e0313239. doi: 10.1371/journal.pone.0313239 (PMC11813106; doi:10.1371/journal.pone.0313239)
Supplement: S3 File — Complete report of Bayesian LMEMs. (PDF) [file pone.0313239.s003.pdf]

Individual differences in associative/semantic priming: Spreading of activation in semantic memory and epistemically unwarranted beliefs – SUPPLEMENTARY MATERIAL: Complete reporting for Bayesian linear mixed-effects models (LMEMs) analyses over response times (RTs)

Daniel Huete-Pérez[1], Robert Davies[2], Javier Rodríguez-Ferreiro[3], and Pilar Ferré[1]

[1] Universitat Rovira i Virgili, Department of Psychology, Research Center for Behavior Assessment (CRAMC), Tarragona, Spain.

[2] Department of Psychology, Lancaster University, Bailrigg, Lancaster, United Kingdom.

[3] Grup de Recerca en Cognició i Llenguatge (GRECIL), Departament de Cognició, Desenvolupament i Psicologia de la Educació, Secció de Processos Cognitius, Institut de Neurociències (INUB), Universitat de Barcelona (UB), Barcelona, Spain.

Abstract: Starting from the *enhanced spreading of activation through semantic memory* (one of the explanatory mechanisms attempting to explain some manifestations observed in schizophrenia) and the *psychosis continuum* (a dimensional approach to psychotic disorders, where ‘normality’ and ‘psychopathology’ are not qualitatively different in nature but placed on varying levels of the same continuum), the main aim of the present research was to explore whether there are individual differences in associative/semantic priming in people with different levels of epistemically unwarranted beliefs (EUB). Participants varying in paranormal, pseudoscientific and conspiracy endorsement completed a primed lexical decision task containing related prime-target words (e.g., bulb-light) and unrelated prime-target words (e.g., sock-light). Bayesian linear mixed-effects models over response times (RTs) revealed a main direct priming effect (faster RTs in related pairs than in unrelated ones), a main facilitatory effect for some EUB scores (i.e., the higher the value for EUB score, the faster RTs), and an interactive effect between the experimental manipulation

and some EUB scores (the higher the EUB score, the smaller the direct priming effect). These results are consistent with predictions made from the enhanced spreading of activation explanatory mechanism, but other alternative accounts are also discussed.

Keywords: associative priming, semantic priming, paranormal beliefs, pseudoscientific beliefs, conspiracy beliefs.

Table S3.1

Bayesian LMEM summary (PEUBI-S as EUB score, RTs as dependant variable)

Fixed (population-level) effects						
	Estimate	95% CrI	*SE*	$\hat{R}$	ESS	
					Bulk	Tail
Intercept	534.50	[524.91, 544.08]	4.91	1.00	3210	7304
P_AoA	3.11	[0.70, 5.46]	1.22	1.00	27810	25506
P_Conc	2.45	[-0.00, 4.93]	1.27	1.00	21856	24649
P_Fam	-3.46	[-5.57, -1.38]	1.07	1.00	29589	26838
P_Val	-1.44	[-3.78, 0.93]	1.20	1.00	23103	24069
P_Aro	0.12	[-1.71, 1.94]	0.94	1.00	31523	27738
P_NLD_Spanish_Catalan	-0.65	[-2.34, 1.04]	0.86	1.00	31571	26678
P_prevalence_nts	-1.20	[-3.01, 0.63]	0.92	1.00	30305	25389
P_log_frq	-1.77	[-4.45, 0.91]	1.37	1.00	21077	23416
P_num_letters	2.39	[-0.19, 4.95]	1.31	1.00	22553	25178
P_N	2.60	[0.28, 4.93]	1.19	1.00	25795	25733
P_abs_tok_MBOF	0.60	[-1.06, 2.26]	0.85	1.00	31293	27294
T_AoA	2.53	[0.01, 5.04]	1.29	1.00	22823	25037
T_Conc	-0.83	[-3.35, 1.70]	1.28	1.00	22429	23539
T_Fam	-3.43	[-5.71, -1.17]	1.16	1.00	22511	23696
T_Val	-0.97	[-3.31, 1.37]	1.19	1.00	23798	24634
T_Aro	-2.13	[-4.42, 0.15]	1.17	1.00	23937	24867
T_NLD_Spanish_Catalan	-0.99	[-3.04, 1.04]	1.04	1.00	23658	24608
T_prevalence_nts	-0.64	[-2.60, 1.35]	1.01	1.00	24148	23883
T_log_frq	-8.74	[-11.25, -6.21]	1.27	1.00	20187	24485

T_num_letters	3.22	[0.24, 6.16]	1.51	1.00	20520	23280
T_N	2.30	[-0.46, 5.04]	1.40	1.00	21347	23774
T_abs_tok_MBOF	1.54	[-0.62, 3.69]	1.10	1.00	23684	25319
Trial	-2.66	[-3.68, -1.67]	0.51	1.00	78248	23314
PrevRT_man	10.42	[9.37, 11.48]	0.53	1.00	70349	24721
PrevERR	-2.13	[-4.33, 0.07]	1.13	1.00	77414	23502
Relatedness	-10.10	[-11.72, -8.48]	0.83	1.00	26499	25371
List	1.48	[-7.53, 10.30]	4.54	1.00	3306	7015
PEUBI_S	-12.14	[-21.32, -3.09]	4.61	1.00	3882	7154
Relatedness:List	-2.30	[-4.46, -0.14]	1.11	1.00	22529	24174
Relatedness:PEUBI_S	0.88	[-0.38, 2.14]	0.64	1.00	27635	25729
Random (group-level) effects – By-participant						
	Estimate	95% CrI	*SE*	$\hat{R}$	ESS	
					Bulk	Tail
sd(Intercept)	45.53	[39.48, 52.62]	3.36	1.00	5685	11151
sd(Relatedness)	4.09	[2.58, 5.63]	0.78	1.00	12131	16109
cor(Intercept,Relatedness)	-0.39	[-0.66, -0.09]	0.15	1.00	28318	21977
Random (group-level) effects – By-target						
	Estimate	95% CrI	*SE*	$\hat{R}$	ESS	
					Bulk	Tail
sd(Intercept)	11.83	[10.19, 13.63]	0.88	1.00	13568	21497
sd(Relatedness)	6.91	[5.43, 8.46]	0.77	1.00	13668	20935
sd(PEUBI_S)	1.58	[0.12, 3.22]	0.83	1.00	10525	13340
cor(Intercept,Relatedness)	-0.15	[-0.40, 0.13]	0.13	1.00	13998	22086
cor(Intercept,PEUBI_S)	-0.04	[-0.64, 0.59]	0.31	1.00	43460	23056

cor(Relatedness,PEUBI_S)	0.47	[-0.31, 0.90]	0.31	1.00	26990	20668
Family-specific parameters (Ex-Gaussian distribution)						
	Estimate	95% CrI	*SE*	$\hat{R}$	ESS	
					Bulk	Tail
sigma	35.90	[34.77, 37.05]	0.59	1.00	52574	26146
beta	92.72	[90.83, 94.66]	0.98	1.00	59053	26432

Note. **Model** = RT ~ 1 + P_AoA + P_Conc + P_Fam + P_Val + P_Aro + P_NLD_Spanish_Catalan + P_prevalence_nts + P_log_frq + P_num_letters + P_N + P_abs_tok_MBOF + T_AoA + T_Conc + T_Fam + T_Val + T_Aro + T_NLD_Spanish_Catalan + T_prevalence_nts + T_log_frq + T_num_letters + T_N + T_abs_tok_MBOF + Trial + PrevRT_man + PrevERR + Relatedness + List + PEUBI_S + Relatedness:List + Relatedness:PEUBI_S + (1 + Relatedness | Participant) + (1 + Relatedness + PEUBI_S | Target); **P_AoA =** prime word Age of acquisition; **P_Conc =** prime word Concreteness; **P_Fam =** prime word Familiarity; **P_Val =** prime word Valence; **P_Aro =** prime word Arousal; **P_NLD_Spanish_Catalan =** prime word Normalised Levenshtein distance between Spanish–Catalan translations; **P_prevalence_nts =** prime word Prevalence; **P_log_frq =** prime word Frequency (logarithmic scale); **P_num_letters =** prime word Length (number of letters); **P_N =** prime word Number of orthographic neighbours; **P_abs_tok_MBOF =** prime word Bigram frequency (logarithmic scale); **T_AoA =** target word Age of acquisition; **T_Conc =** target word Concreteness; **T_Fam =** target word Familiarity; **T_Val =** target word Valence; **T_Aro =** target word Arousal; **T_NLD_Spanish_Catalan =** target word Normalised Levenshtein distance between Spanish–Catalan translations; **T_prevalence_nts =** target word Prevalence; **T_log_frq =** target word Frequency (logarithmic scale); **T_num_letters =** target word Length (number of letters); **T_N =** target word Number of orthographic neighbours; **T_abs_tok_MBOF =** target word Bigram frequency (logarithmic scale); **Trial** = Trial order; **PrevRT_man =** RT of the preceding trial; **PrevERR =** Response accuracy of the preceding trial (sum-coded as *−1 = incorrect* and *+1 = correct*); **Relatedness =** Prime-target relatedness (sum-coded as *−1 = unrelated* and *+1 = related*); **List** (sum-coded as *−1 = list B* and *+1 = list A*); **PEUBI_S** = superstitions

(EUB score); $\boldsymbol{\hat{R}}$ = Gelman-Rubin convergence diagnostic; **ESS =** effective sample size; **sd() =** standard deviation for the random effect inside the parenthesis; **cor() =** correlation between the random effects inside the parenthesis.

Figure S3.1

Posterior distributions of fixed effects with 95% CrI in red (PEUBI-S as EUB score, RTs as dependant variable)

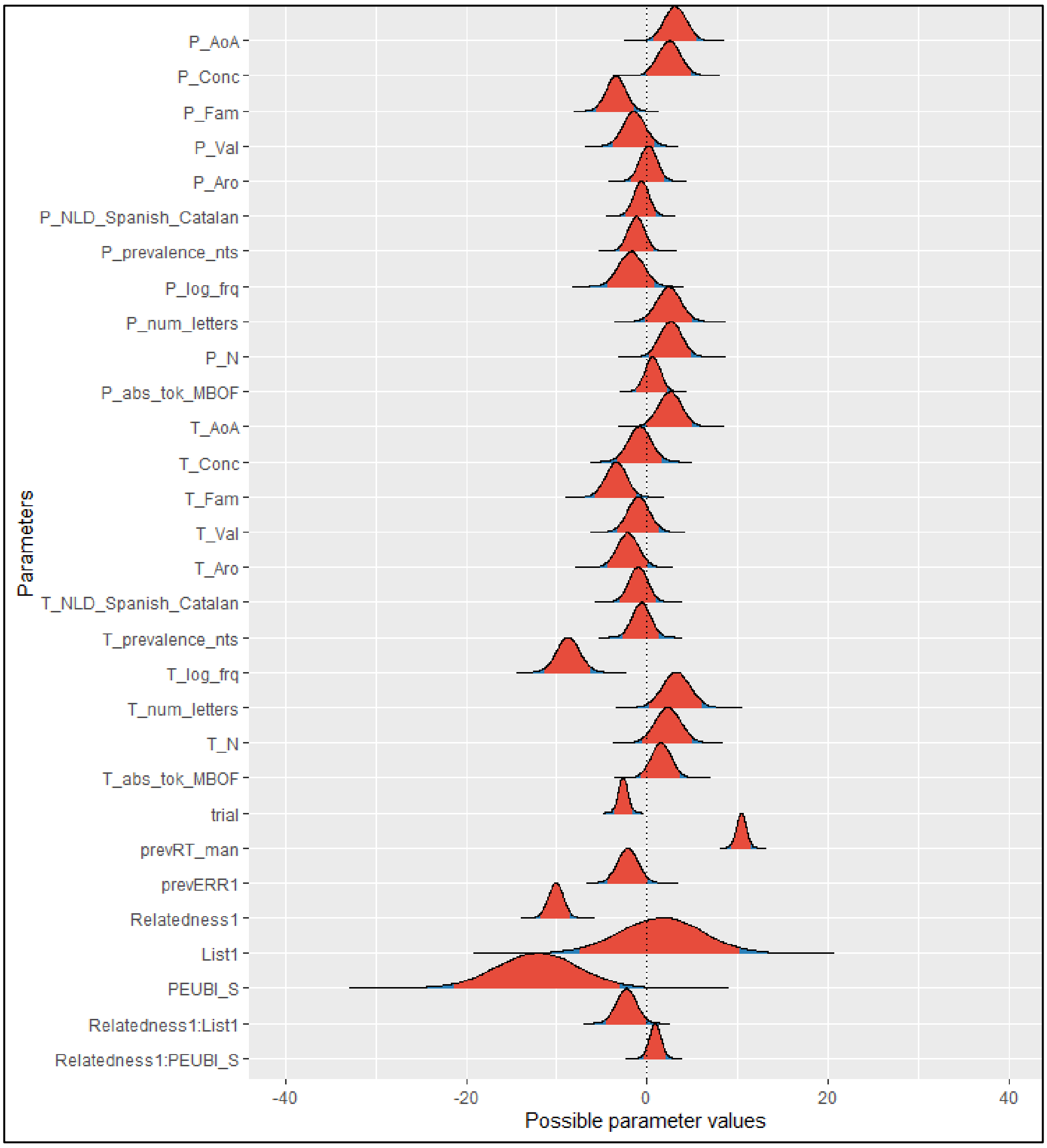

Note. **P_AoA** = prime word Age of acquisition; **P_Conc** = prime word Concreteness; **P_Fam** = prime word Familiarity; **P_Val** = prime word Valence; **P_Aro** = prime word Arousal; **P_NLD_Spanish_Catalan** = prime word Normalised Levenshtein distance between Spanish–Catalan translations; **P_prevalence_nts** = prime word Prevalence; **P_log_frq** = prime word Frequency (logarithmic scale); **P_num_letters** = prime word Length (number of letters); **P_N** = prime word Number of orthographic neighbours; **P_abs_tok_MBOF** = prime word Bigram frequency (logarithmic scale);

T_AoA = target word Age of acquisition; **T_Conc =** target word Concreteness; **T_Fam =** target word Familiarity; **T_Val =** target word Valence; **T_Aro =** target word Arousal; **T_NLD_Spanish_Catalan =** target word Normalised Levenshtein distance between Spanish–Catalan translations; **T_prevalence_nts =** target word Prevalence; **T_log_frq =** target word Frequency (logarithmic scale); **T_num_letters =** target word Length (number of letters); **T_N =** target word Number of orthographic neighbours; **T_abs_tok_MBOF =** target word Bigram frequency (logarithmic scale); **Trial** = Trial order; **PrevRT_man =** RT of the preceding trial; **PrevERR =** Response accuracy of the preceding trial (sum-coded as *−1 = incorrect* and *+1 = correct*); **Relatedness =** Prime-target relatedness (sum-coded as *−1 = unrelated* and *+1 = related*); **List** (sum-coded as *−1 = list B* and *+1 = list A*); **PEUBI_S** = superstitions (EUB score).

Table S3.2

Bayesian LMEM summary (PEUBI-OP as EUB score, RTs as dependant variable)

Fixed (population-level) effects						
	Estimate	95% CrI	*SE*	$\hat{R}$	ESS	
					Bulk	Tail
Intercept	534.39	[524.85, 543.98]	4.87	1.00	3403	6936
P_AoA	3.09	[0.70, 5.49]	1.22	1.00	27516	25454
P_Conc	2.42	[-0.09, 4.93]	1.27	1.00	22204	23247
P_Fam	-3.47	[-5.58, -1.36]	1.07	1.00	27929	24939
P_Val	-1.42	[-3.84, 0.98]	1.22	1.00	21401	23446
P_Aro	0.17	[-1.70, 2.02]	0.95	1.00	29822	24407
P_NLD_Spanish_Catalan	-0.61	[-2.31, 1.11]	0.87	1.00	28666	26548
P_prevalence_nts	-1.17	[-3.00, 0.64]	0.93	1.00	28427	25915
P_log_frq	-1.82	[-4.56, 0.90]	1.38	1.00	21080	23560
P_num_letters	2.34	[-0.22, 4.94]	1.31	1.00	21795	22285
P_N	2.53	[0.19, 4.84]	1.18	1.00	24535	24792
P_abs_tok_MBOF	0.65	[-1.03, 2.36]	0.86	1.00	28465	25913
T_AoA	2.59	[0.07, 5.13]	1.28	1.00	22746	24288
T_Conc	-0.81	[-3.32, 1.68]	1.28	1.00	22196	23850
T_Fam	-3.45	[-5.73, -1.21]	1.15	1.00	23510	24506
T_Val	-0.95	[-3.27, 1.43]	1.19	1.00	22035	23611
T_Aro	-2.16	[-4.44, 0.16]	1.17	1.00	22752	24120
T_NLD_Spanish_Catalan	-1.02	[-3.07, 1.01]	1.04	1.00	22977	23520
T_prevalence_nts	-0.67	[-2.67, 1.34]	1.01	1.00	22753	23439
T_log_frq	-8.68	[-11.19, -6.18]	1.27	1.00	19316	23330

T_num_letters	3.27	[0.31, 6.23]	1.50	1.00	20834	21142
T_N	2.29	[-0.46, 5.00]	1.39	1.00	21104	23005
T_abs_tok_MBOF	1.52	[-0.61, 3.71]	1.10	1.00	22475	24767
Trial	-2.66	[-3.66, -1.66]	0.51	1.00	67912	24378
PrevRT_man	10.42	[9.37, 11.48]	0.54	1.00	66057	24279
PrevERR	-2.08	[-4.31, 0.12]	1.13	1.00	64838	24457
Relatedness	-10.12	[-11.74, -8.50]	0.82	1.00	24270	23976
List	0.67	[-8.43, 9.93]	4.72	1.00	3161	6737
PEUBI_OP	-10.51	[-19.58, -1.27]	4.71	1.00	3867	8230
Relatedness:List	-2.25	[-4.42, -0.07]	1.10	1.00	22543	23730
Relatedness:PEUBI_OP	0.67	[-0.61, 1.96]	0.66	1.00	26528	25846
Random (group-level) effects – By-participant						
	Estimate	95% CrI	*SE*	$\hat{R}$	ESS	
					Bulk	Tail
sd(Intercept)	45.85	[39.82, 52.92]	3.35	1.00	6093	11402
sd(Relatedness)	4.14	[2.66, 5.65]	0.76	1.00	12366	16960
cor(Intercept,Relatedness)	-0.40	[-0.67, -0.10]	0.15	1.00	26634	21320
Random (group-level) effects – By-target						
	Estimate	95% CrI	*SE*	$\hat{R}$	ESS	
					Bulk	Tail
sd(Intercept)	11.85	[10.20, 13.67]	0.89	1.00	12231	21014
sd(Relatedness)	6.96	[5.46, 8.52]	0.78	1.00	12925	20515
sd(PEUBI_OP)	1.30	[0.06, 3.22]	0.87	1.00	10443	15412
cor(Intercept,Relatedness)	-0.15	[-0.40, 0.12]	0.13	1.00	13730	20231
cor(Intercept,PEUBI_OP)	-0.13	[-0.75, 0.60]	0.34	1.00	41388	21766

cor(Relatedness,PEUBI_OP)	0.21	[-0.57, 0.80]	0.35	1.00	37007	23738
Family-specific parameters (Ex-Gaussian distribution)						
	Estimate	95% CrI	*SE*	$\hat{R}$	ESS	
					Bulk	Tail
sigma	35.91	[34.79, 37.07]	0.58	1.00	47996	24711
beta	92.73	[90.83, 94.64]	0.97	1.00	52751	25703

Note. **Model** = RT ~ 1 + P_AoA + P_Conc + P_Fam + P_Val + P_Aro + P_NLD_Spanish_Catalan + P_prevalence_nts + P_log_frq + P_num_letters + P_N + P_abs_tok_MBOF + T_AoA + T_Conc + T_Fam + T_Val + T_Aro + T_NLD_Spanish_Catalan + T_prevalence_nts + T_log_frq + T_num_letters + T_N + T_abs_tok_MBOF + Trial + PrevRT_man + PrevERR + Relatedness + List + PEUBI_OP + Relatedness:List + Relatedness:PEUBI_OP + (1 + Relatedness | Participant) + (1 + Relatedness + PEUBI_OP | Target); **P_AoA =** prime word Age of acquisition; **P_Conc =** prime word Concreteness; **P_Fam =** prime word Familiarity; **P_Val =** prime word Valence; **P_Aro =** prime word Arousal; **P_NLD_Spanish_Catalan =** prime word Normalised Levenshtein distance between Spanish–Catalan translations; **P_prevalence_nts =** prime word Prevalence; **P_log_frq =** prime word Frequency (logarithmic scale); **P_num_letters =** prime word Length (number of letters); **P_N =** prime word Number of orthographic neighbours; **P_abs_tok_MBOF =** prime word Bigram frequency (logarithmic scale); **T_AoA =** target word Age of acquisition; **T_Conc =** target word Concreteness; **T_Fam =** target word Familiarity; **T_Val =** target word Valence; **T_Aro =** target word Arousal; **T_NLD_Spanish_Catalan =** target word Normalised Levenshtein distance between Spanish–Catalan translations; **T_prevalence_nts =** target word Prevalence; **T_log_frq =** target word Frequency (logarithmic scale); **T_num_letters =** target word Length (number of letters); **T_N =** target word Number of orthographic neighbours; **T_abs_tok_MBOF =** target word Bigram frequency (logarithmic scale); **Trial** = Trial order; **PrevRT_man =** RT of the preceding trial; **PrevERR =** Response accuracy of the preceding trial (sum-coded as *−1 = incorrect* and *+1 = correct*); **Relatedness =** Prime-target relatedness (sum-coded as *−1 = unrelated* and *+1 = related*); **List** (sum-coded as *−1 = list B* and *+1 = list A*); **PEUBI_OP** = occultism

and pseudoscience (EUB score); $\hat{\boldsymbol{R}}$ = Gelman-Rubin convergence diagnostic; **ESS =** effective sample size; **sd() =** standard deviation for the random effect inside the parenthesis; **cor() =** correlation between the random effects inside the parenthesis.

Figure S3.2

Posterior distributions of fixed effects with 95% CrI in red (PEUBI-OP as EUB score, RTs as dependant variable)

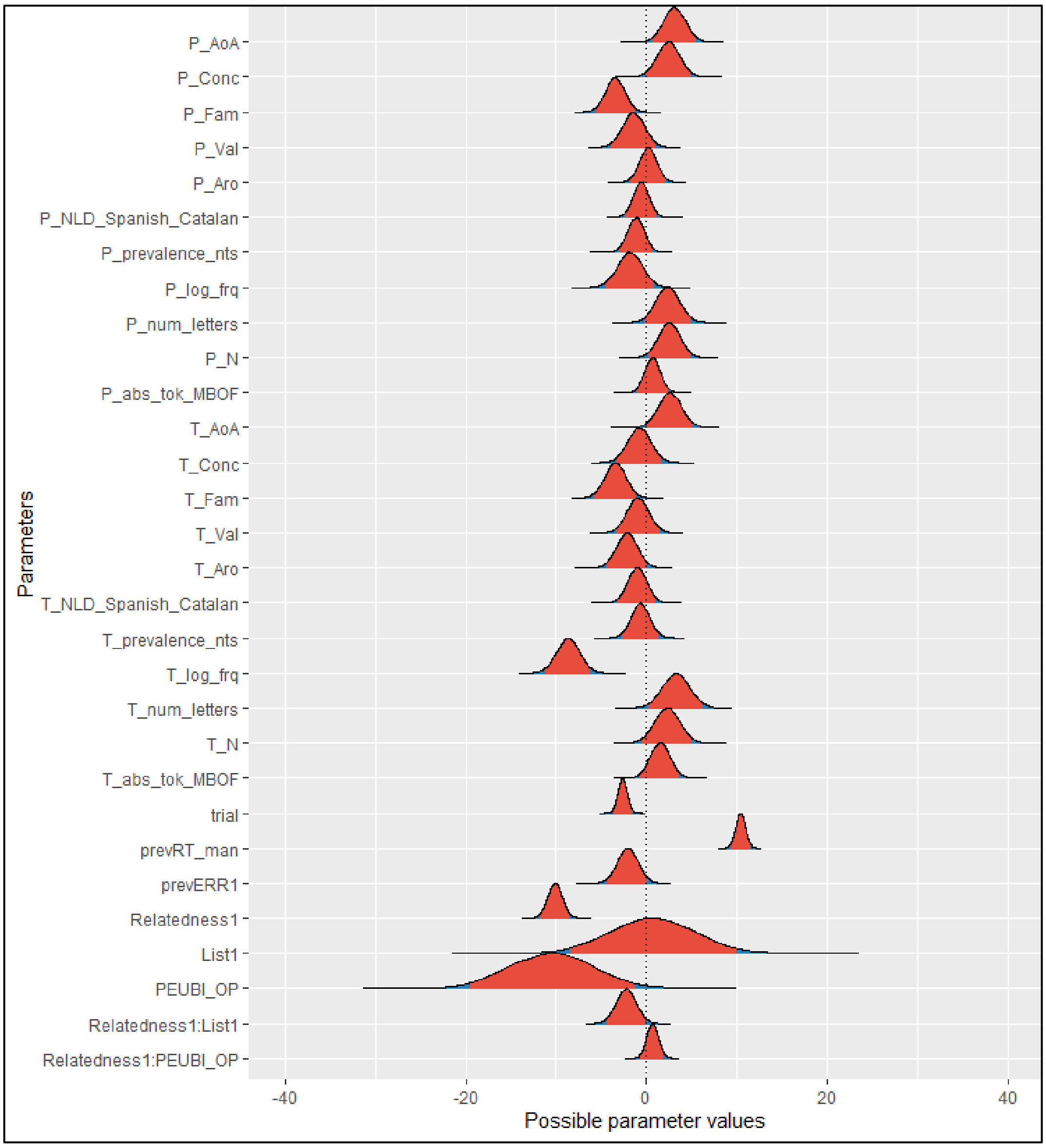

Note. **P_AoA** = prime word Age of acquisition; **P_Conc** = prime word Concreteness; **P_Fam** = prime word Familiarity; **P_Val** = prime word Valence; **P_Aro** = prime word Arousal; **P_NLD_Spanish_Catalan** = prime word Normalised Levenshtein distance between Spanish–Catalan translations; **P_prevalence_nts** = prime word Prevalence; **P_log_frq** = prime word Frequency (logarithmic scale); **P_num_letters** = prime word Length (number of letters); **P_N** = prime word Number of orthographic neighbours; **P_abs_tok_MBOF** = prime word Bigram frequency (logarithmic scale);

T_AoA = target word Age of acquisition; **T_Conc =** target word Concreteness; **T_Fam =** target word Familiarity; **T_Val =** target word Valence; **T_Aro =** target word Arousal; **T_NLD_Spanish_Catalan =** target word Normalised Levenshtein distance between Spanish–Catalan translations; **T_prevalence_nts =** target word Prevalence; **T_log_frq =** target word Frequency (logarithmic scale); **T_num_letters =** target word Length (number of letters); **T_N =** target word Number of orthographic neighbours; **T_abs_tok_MBOF =** target word Bigram frequency (logarithmic scale); **Trial** = Trial order; **PrevRT_man =** RT of the preceding trial; **PrevERR =** Response accuracy of the preceding trial (sum-coded as *−1 = incorrect* and *+1 = correct*); **Relatedness =** Prime-target relatedness (sum-coded as *−1 = unrelated* and *+1 = related*); **List** (sum-coded as *−1 = list B* and *+1 = list A*); **PEUBI_OP** = occultism and pseudoscience (EUB score).

Table S3.3

Bayesian LMEM summary (PEUBI-TR as EUB score, RTs as dependant variable)

Fixed (population-level) effects						
	Estimate	95% CrI	*SE*	$\hat{R}$	ESS	
					Bulk	Tail
Intercept	534.38	[524.71, 544.04]	4.91	1.00	2242	4952
P_AoA	3.27	[0.84, 5.69]	1.23	1.00	19195	22354
P_Conc	2.58	[0.11, 5.04]	1.26	1.00	16587	21497
P_Fam	-3.40	[-5.51, -1.30]	1.07	1.00	21198	23027
P_Val	-1.38	[-3.72, 0.93]	1.19	1.00	17029	21504
P_Aro	0.02	[-1.83, 1.89]	0.95	1.00	19819	22130
P_NLD_Spanish_Catalan	-0.64	[-2.33, 1.03]	0.86	1.00	21601	23120
P_prevalence_nts	-1.04	[-2.86, 0.78]	0.92	1.00	21787	23053
P_log_frq	-1.84	[-4.55, 0.82]	1.37	1.00	15297	20601
P_num_letters	2.22	[-0.37, 4.79]	1.31	1.00	17312	20339
P_N	2.42	[0.12, 4.73]	1.17	1.00	18209	22945
P_abs_tok_MBOF	0.80	[-0.88, 2.51]	0.86	1.00	22344	23596
T_AoA	2.45	[-0.07, 5.01]	1.29	1.00	16170	19386
T_Conc	-0.86	[-3.37, 1.65]	1.28	1.00	16344	20445
T_Fam	-3.57	[-5.84, -1.32]	1.15	1.00	15789	20349
T_Val	-0.92	[-3.26, 1.41]	1.19	1.00	16585	21407
T_Aro	-1.97	[-4.25, 0.36]	1.17	1.00	15887	21367
T_NLD_Spanish_Catalan	-0.99	[-3.01, 1.03]	1.03	1.00	17145	23688
T_prevalence_nts	-0.78	[-2.76, 1.20]	1.01	1.00	17133	21379
T_log_frq	-8.71	[-11.20, -6.25]	1.27	1.00	14451	18960

T_num_letters	3.13	[0.21, 6.02]	1.49	1.00	15688	20469
T_N	2.38	[-0.34, 5.10]	1.38	1.00	15756	20247
T_abs_tok_MBOF	1.45	[-0.70, 3.61]	1.10	1.00	16256	20278
Trial	-2.66	[-3.67, -1.66]	0.51	1.00	58632	23611
PrevRT_man	10.42	[9.38, 11.45]	0.53	1.00	53852	24028
PrevERR	-2.10	[-4.30, 0.14]	1.14	1.00	57275	23686
Relatedness	-10.13	[-11.77, -8.51]	0.83	1.00	18774	22992
List	1.00	[-7.90, 10.16]	4.63	1.00	2108	4172
PEUBI_TR	-10.78	[-19.99, -1.59]	4.66	1.00	2933	5518
Relatedness:List	-2.35	[-4.51, -0.16]	1.11	1.00	15096	22129
Relatedness:PEUBI_TR	0.56	[-0.72, 1.82]	0.65	1.00	19229	22562
Random (group-level) effects – By-participant						
	Estimate	95% CrI	*SE*	$\hat{R}$	ESS	
					Bulk	Tail
sd(Intercept)	45.85	[39.82, 53.05]	3.41	1.00	4526	8323
sd(Relatedness)	4.18	[2.68, 5.69]	0.77	1.00	13026	16493
cor(Intercept,Relatedness)	-0.40	[-0.67, -0.11]	0.14	1.00	21418	20844
Random (group-level) effects – By-target						
	Estimate	95% CrI	*SE*	$\hat{R}$	ESS	
					Bulk	Tail
sd(Intercept)	11.88	[10.25, 13.65]	0.87	1.00	12530	20601
sd(Relatedness)	6.97	[5.47, 8.51]	0.77	1.00	12928	19858
sd(PEUBI_TR)	2.02	[0.26, 3.70]	0.88	1.00	8555	7223
cor(Intercept,Relatedness)	-0.15	[-0.40, 0.12]	0.13	1.00	11413	18831
cor(Intercept,PEUBI_TR)	-0.42	[-0.84, 0.17]	0.26	1.00	24965	18153

cor(Relatedness,PEUBI_TR)	0.40	[-0.24, 0.85]	0.28	1.00	23428	18801
Family-specific parameters (Ex-Gaussian distribution)						
	Estimate	95% CrI	*SE*	$\hat{R}$	ESS	
					Bulk	Tail
sigma	35.89	[34.77, 37.04]	0.58	1.00	42206	25167
beta	92.70	[90.82, 94.64]	0.97	1.00	47553	25735

Note. **Model** = RT ~ 1 + P_AoA + P_Conc + P_Fam + P_Val + P_Aro + P_NLD_Spanish_Catalan + P_prevalence_nts + P_log_frq + P_num_letters + P_N + P_abs_tok_MBOF + T_AoA + T_Conc + T_Fam + T_Val + T_Aro + T_NLD_Spanish_Catalan + T_prevalence_nts + T_log_frq + T_num_letters + T_N + T_abs_tok_MBOF + Trial + PrevRT_man + PrevERR + Relatedness + List + PEUBI_TR + Relatedness:List + Relatedness:PEUBI_TR + (1 + Relatedness | Participant) + (1 + Relatedness + PEUBI_TR | Target); **P_AoA =** prime word Age of acquisition; **P_Conc =** prime word Concreteness; **P_Fam =** prime word Familiarity; **P_Val =** prime word Valence; **P_Aro =** prime word Arousal; **P_NLD_Spanish_Catalan =** prime word Normalised Levenshtein distance between Spanish–Catalan translations; **P_prevalence_nts =** prime word Prevalence; **P_log_frq =** prime word Frequency (logarithmic scale); **P_num_letters =** prime word Length (number of letters); **P_N =** prime word Number of orthographic neighbours; **P_abs_tok_MBOF =** prime word Bigram frequency (logarithmic scale); **T_AoA =** target word Age of acquisition; **T_Conc =** target word Concreteness; **T_Fam =** target word Familiarity; **T_Val =** target word Valence; **T_Aro =** target word Arousal; **T_NLD_Spanish_Catalan =** target word Normalised Levenshtein distance between Spanish–Catalan translations; **T_prevalence_nts =** target word Prevalence; **T_log_frq =** target word Frequency (logarithmic scale); **T_num_letters =** target word Length (number of letters); **T_N =** target word Number of orthographic neighbours; **T_abs_tok_MBOF =** target word Bigram frequency (logarithmic scale); **Trial** = Trial order; **PrevRT_man =** RT of the preceding trial; **PrevERR =** Response accuracy of the preceding trial (sum-coded as −*1* = *incorrect* and +*1* = *correct*); **Relatedness =** Prime-target relatedness (sum-coded as −*1* = *unrelated* and +*1* = *related*); **List** (sum-coded as −*1* = *list B* and +*1* = *list A*); **PEUBI_TR** = traditional

religion (EUB score); $\hat{R}$ = Gelman-Rubin convergence diagnostic; **ESS =** effective sample size; **sd() =** standard deviation for the random effect inside the parenthesis; **cor() =** correlation between the random effects inside the parenthesis.

Figure S3.3

Posterior distributions of fixed effects with 95% CrI in red (PEUBI-TR as EUB score, RTs as dependant variable)

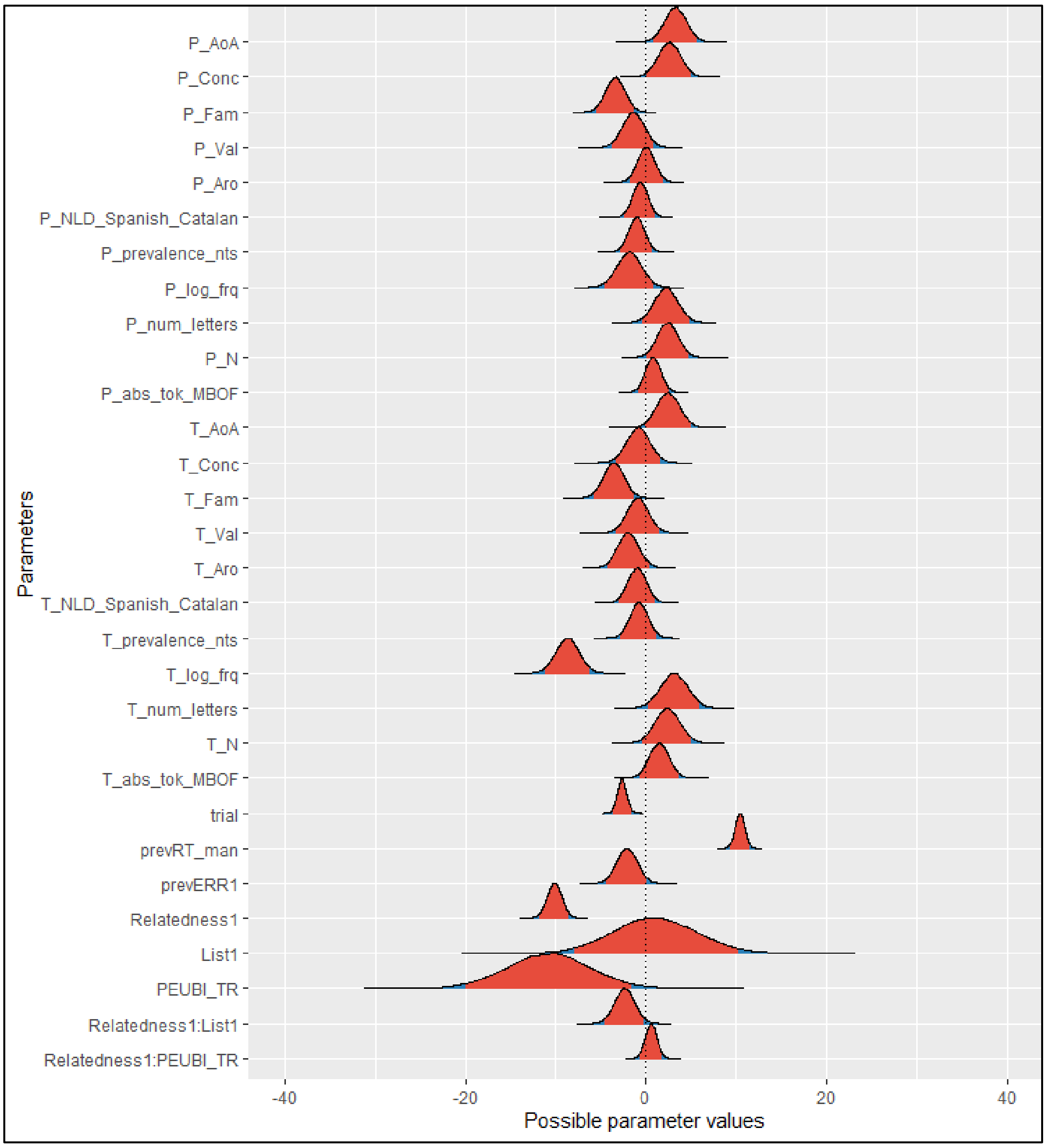

Note. **P_AoA** = prime word Age of acquisition; **P_Conc** = prime word Concreteness; **P_Fam** = prime word Familiarity; **P_Val** = prime word Valence; **P_Aro** = prime word Arousal; **P_NLD_Spanish_Catalan** = prime word Normalised Levenshtein distance between Spanish–Catalan translations; **P_prevalence_nts** = prime word Prevalence; **P_log_frq** = prime word Frequency (logarithmic scale); **P_num_letters** = prime word Length (number of letters); **P_N** = prime word Number of orthographic neighbours; **P_abs_tok_MBOF** = prime word Bigram frequency (logarithmic scale);

T_AoA = target word Age of acquisition; **T_Conc =** target word Concreteness; **T_Fam =** target word Familiarity; **T_Val =** target word Valence; **T_Aro =** target word Arousal; **T_NLD_Spanish_Catalan =** target word Normalised Levenshtein distance between Spanish–Catalan translations; **T_prevalence_nts =** target word Prevalence; **T_log_frq =** target word Frequency (logarithmic scale); **T_num_letters =** target word Length (number of letters); **T_N =** target word Number of orthographic neighbours; **T_abs_tok_MBOF =** target word Bigram frequency (logarithmic scale); **Trial** = Trial order; **PrevRT_man =** RT of the preceding trial; **PrevERR =** Response accuracy of the preceding trial (sum-coded as *−1 = incorrect* and *+1 = correct*); **Relatedness =** Prime-target relatedness (sum-coded as *−1 = unrelated* and *+1 = related*); **List** (sum-coded as *−1 = list B* and *+1 = list A*); **PEUBI_TR** = traditional religion (EUB score).

Table S3.4

Bayesian LMEM summary (PEUBI-ELF as EUB score, RTs as dependant variable)

Fixed (population-level) effects						
	Estimate	95% CrI	*SE*	$\hat{R}$	ESS	
					Bulk	Tail
Intercept	534.38	[524.49, 544.29]	5.05	1.00	3027	6796
P_AoA	3.10	[0.70, 5.49]	1.22	1.00	26576	26057
P_Conc	2.46	[-0.08, 4.96]	1.28	1.00	20538	24330
P_Fam	-3.49	[-5.61, -1.38]	1.08	1.00	27487	25435
P_Val	-1.42	[-3.81, 0.93]	1.21	1.00	21478	24689
P_Aro	0.16	[-1.67, 2.01]	0.94	1.00	29148	26610
P_NLD_Spanish_Catalan	-0.58	[-2.24, 1.10]	0.85	1.00	31525	26843
P_prevalence_nts	-1.20	[-3.01, 0.61]	0.92	1.00	27149	25356
P_log_frq	-1.80	[-4.51, 0.88]	1.38	1.00	18765	23640
P_num_letters	2.35	[-0.21, 4.93]	1.31	1.00	20724	23461
P_N	2.57	[0.23, 4.91]	1.19	1.00	22951	25100
P_abs_tok_MBOF	0.62	[-1.04, 2.30]	0.86	1.00	28464	26661
T_AoA	2.54	[0.04, 5.05]	1.28	1.00	20832	24270
T_Conc	-0.83	[-3.34, 1.68]	1.28	1.00	21399	23260
T_Fam	-3.44	[-5.65, -1.21]	1.14	1.00	21653	24608
T_Val	-0.97	[-3.31, 1.40]	1.20	1.00	21435	24631
T_Aro	-2.15	[-4.46, 0.18]	1.18	1.00	22796	23633
T_NLD_Spanish_Catalan	-1.00	[-3.02, 1.05]	1.04	1.00	21961	23299
T_prevalence_nts	-0.63	[-2.63, 1.38]	1.01	1.00	22435	24370
T_log_frq	-8.71	[-11.20, -6.22]	1.27	1.00	18516	22266

T_num_letters	3.23	[0.29, 6.19]	1.51	1.00	20500	23946
T_N	2.30	[-0.42, 5.00]	1.38	1.00	21174	24107
T_abs_tok_MBOF	1.56	[-0.60, 3.70]	1.10	1.00	21802	24188
Trial	-2.66	[-3.65, -1.68]	0.51	1.00	79199	22076
PrevRT_man	10.43	[9.37, 11.47]	0.53	1.00	73645	25051
PrevERR	-2.10	[-4.33, 0.14]	1.15	1.00	76830	24086
Relatedness	-10.10	[-11.74, -8.48]	0.83	1.00	23909	25108
List	0.49	[-9.03, 10.04]	4.82	1.00	2943	5747
PEUBI_ELF	-2.49	[-11.86, 6.82]	4.72	1.00	3542	7890
Relatedness:List	-2.22	[-4.39, -0.02]	1.11	1.00	19760	24362
Relatedness:PEUBI_ELF	0.28	[-1.02, 1.55]	0.66	1.00	25689	25374
Random (group-level) effects – By-participant						
	Estimate	95% CrI	*SE*	$\hat{R}$	ESS	
					Bulk	Tail
sd(Intercept)	47.05	[40.84, 54.43]	3.47	1.00	5320	10749
sd(Relatedness)	4.18	[2.68, 5.70]	0.76	1.00	12966	17226
cor(Intercept,Relatedness)	-0.41	[-0.69, -0.12]	0.14	1.00	25103	20687
Random (group-level) effects – By-target						
	Estimate	95% CrI	*SE*	$\hat{R}$	ESS	
					Bulk	Tail
sd(Intercept)	11.84	[10.22, 13.67]	0.88	1.00	13479	21041
sd(Relatedness)	6.95	[5.47, 8.51]	0.77	1.00	13743	22192
sd(PEUBI_ELF)	1.18	[0.05, 2.94]	0.79	1.00	11273	15564
cor(Intercept,Relatedness)	-0.15	[-0.40, 0.12]	0.13	1.00	11867	20087
cor(Intercept,PEUBI_ELF)	-0.03	[-0.68, 0.65]	0.34	1.00	53837	23039

cor(Relatedness,PEUBI_ELF)	0.29	[-0.53, 0.85]	0.36	1.00	33586	23496
Family-specific parameters (Ex-Gaussian distribution)						
	Estimate	95% CrI	*SE*	$\hat{R}$	ESS	
					Bulk	Tail
sigma	35.91	[34.77, 37.06]	0.58	1.00	54516	25767
beta	92.73	[90.83, 94.64]	0.98	1.00	62716	25782

Note. **Model** = RT ~ 1 + P_AoA + P_Conc + P_Fam + P_Val + P_Aro + P_NLD_Spanish_Catalan + P_prevalence_nts + P_log_frq + P_num_letters + P_N + P_abs_tok_MBOF + T_AoA + T_Conc + T_Fam + T_Val + T_Aro + T_NLD_Spanish_Catalan + T_prevalence_nts + T_log_frq + T_num_letters + T_N + T_abs_tok_MBOF + Trial + PrevRT_man + PrevERR + Relatedness + List + PEUBI_ELF + Relatedness:List + Relatedness:PEUBI_ELF + (1 + Relatedness | Participant) + (1 + Relatedness + PEUBI_ELF | Target); **P_AoA =** prime word Age of acquisition; **P_Conc =** prime word Concreteness; **P_Fam =** prime word Familiarity; **P_Val =** prime word Valence; **P_Aro =** prime word Arousal; **P_NLD_Spanish_Catalan** = prime word Normalised Levenshtein distance between Spanish–Catalan translations; **P_prevalence_nts =** prime word Prevalence; **P_log_frq =** prime word Frequency (logarithmic scale); **P_num_letters =** prime word Length (number of letters); **P_N =** prime word Number of orthographic neighbours; **P_abs_tok_MBOF =** prime word Bigram frequency (logarithmic scale); **T_AoA =** target word Age of acquisition; **T_Conc =** target word Concreteness; **T_Fam =** target word Familiarity; **T_Val =** target word Valence; **T_Aro =** target word Arousal; **T_NLD_Spanish_Catalan =** target word Normalised Levenshtein distance between Spanish–Catalan translations; **T_prevalence_nts =** target word Prevalence; **T_log_frq =** target word Frequency (logarithmic scale); **T_num_letters =** target word Length (number of letters); **T_N =** target word Number of orthographic neighbours; **T_abs_tok_MBOF =** target word Bigram frequency (logarithmic scale); **Trial** = Trial order; **PrevRT_man =** RT of the preceding trial; **PrevERR =** Response accuracy of the preceding trial (sum-coded as *−1 = incorrect* and *+1 = correct*); **Relatedness =** Prime-target relatedness (sum-coded as *−1 = unrelated* and *+1 = related*); **List** (sum-coded as *−1 = list B* and *+1 = list A*); **PEUBI_ELF =**

extraordinary life forms (EUB score); $\hat{R}$ = Gelman-Rubin convergence diagnostic; **ESS =** effective sample size; **sd() =** standard deviation for the random effect inside the parenthesis; **cor() =** correlation between the random effects inside the parenthesis.

Figure S3.4

Posterior distributions of fixed effects with 95% CrI in red (PEUBI-ELF as EUB score, RTs as dependant variable)

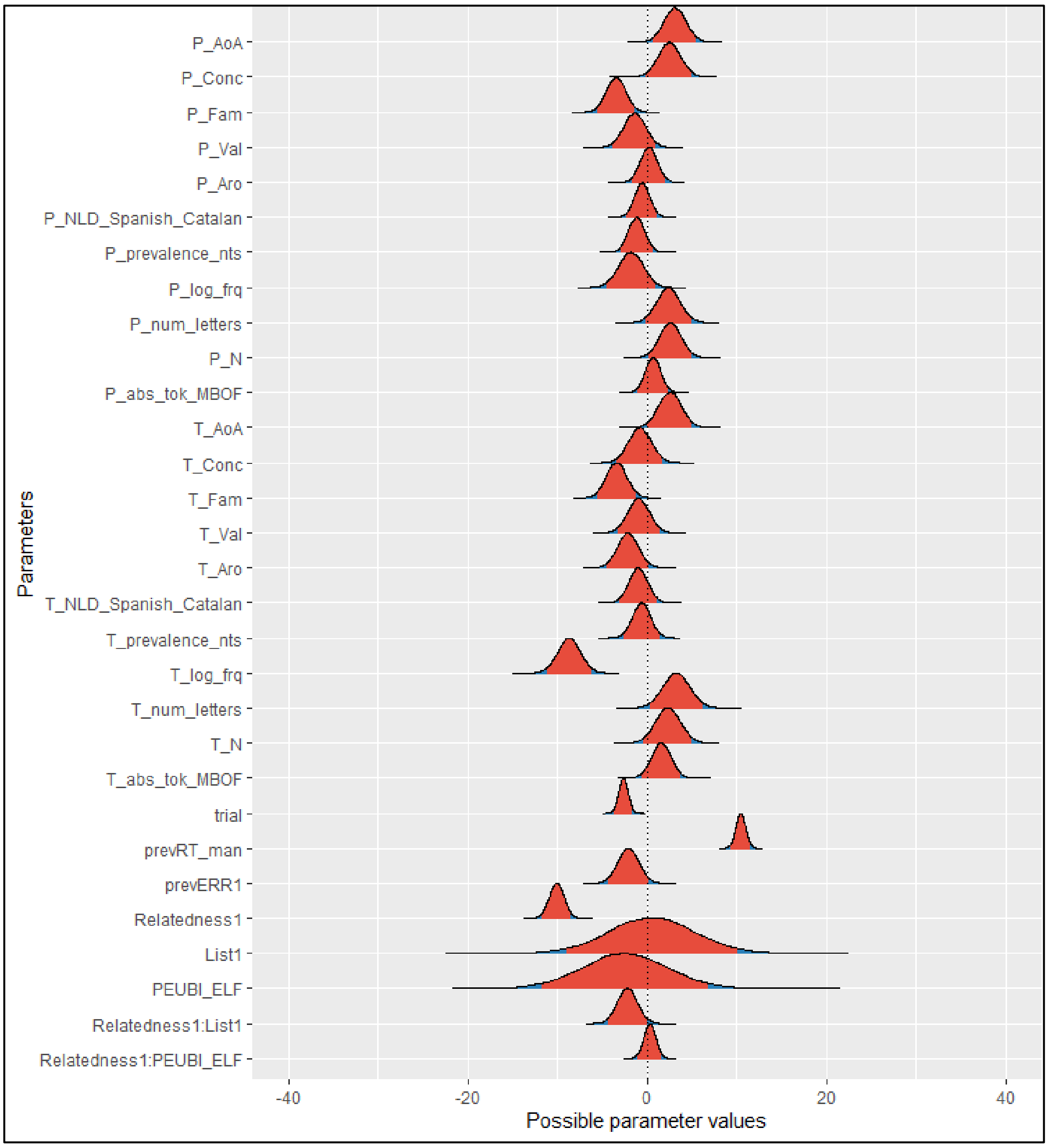

Note. **P_AoA** = prime word Age of acquisition; **P_Conc** = prime word Concreteness; **P_Fam** = prime word Familiarity; **P_Val** = prime word Valence; **P_Aro** = prime word Arousal; **P_NLD_Spanish_Catalan** = prime word Normalised Levenshtein distance between Spanish–Catalan translations; **P_prevalence_nts** = prime word Prevalence; **P_log_frq** = prime word Frequency (logarithmic scale); **P_num_letters** = prime word Length (number of letters); **P_N** = prime word Number of orthographic neighbours; **P_abs_tok_MBOF** = prime word Bigram frequency (logarithmic scale);

T_AoA = target word Age of acquisition; **T_Conc =** target word Concreteness; **T_Fam =** target word Familiarity; **T_Val =** target word Valence; **T_Aro =** target word Arousal; **T_NLD_Spanish_Catalan =** target word Normalised Levenshtein distance between Spanish–Catalan translations; **T_prevalence_nts =** target word Prevalence; **T_log_frq =** target word Frequency (logarithmic scale); **T_num_letters =** target word Length (number of letters); **T_N =** target word Number of orthographic neighbours; **T_abs_tok_MBOF =** target word Bigram frequency (logarithmic scale); **Trial** = Trial order; **PrevRT_man =** RT of the preceding trial; **PrevERR =** Response accuracy of the preceding trial (sum-coded as *−1 = incorrect* and *+1 = correct*); **Relatedness =** Prime-target relatedness (sum-coded as *−1 = unrelated* and *+1 = related*); **List** (sum-coded as *−1 = list B* and *+1 = list A*); **PEUBI_ELF** = extraordinary life forms (EUB score).

Table S3.5

Bayesian LMEM summary (PEUBI-CT as EUB score, RTs as dependant variable)

Fixed (population-level) effects						
	Estimate	95% CrI	*SE*	$\hat{R}$	ESS	
					Bulk	Tail
Intercept	534.37	[524.55, 544.26]	5.02	1.00	2751	6719
P_AoA	3.09	[0.68, 5.45]	1.22	1.00	24420	26260
P_Conc	2.49	[-0.01, 4.97]	1.26	1.00	19514	22938
P_Fam	-3.49	[-5.58, -1.39]	1.08	1.00	26222	25010
P_Val	-1.35	[-3.73, 1.01]	1.22	1.00	21246	23833
P_Aro	0.20	[-1.62, 2.04]	0.93	1.00	26411	26489
P_NLD_Spanish_Catalan	-0.60	[-2.27, 1.08]	0.86	1.00	29315	27140
P_prevalence_nts	-1.16	[-2.98, 0.64]	0.93	1.00	27250	26616
P_log_frq	-1.82	[-4.54, 0.86]	1.37	1.00	19872	23733
P_num_letters	2.38	[-0.23, 5.00]	1.33	1.00	20623	23958
P_N	2.55	[0.24, 4.90]	1.19	1.00	23054	24931
P_abs_tok_MBOF	0.65	[-1.02, 2.32]	0.85	1.00	27272	27254
T_AoA	2.58	[0.08, 5.11]	1.28	1.00	19401	21785
T_Conc	-0.83	[-3.34, 1.67]	1.28	1.00	19681	23171
T_Fam	-3.44	[-5.68, -1.18]	1.14	1.00	20379	23699
T_Val	-1.01	[-3.34, 1.36]	1.20	1.00	20441	23785
T_Aro	-2.18	[-4.45, 0.13]	1.17	1.00	20261	24067
T_NLD_Spanish_Catalan	-1.00	[-3.01, 1.02]	1.03	1.00	20578	24180
T_prevalence_nts	-0.64	[-2.63, 1.36]	1.01	1.00	21401	23724
T_log_frq	-8.70	[-11.17, -6.23]	1.26	1.00	18267	23252

T_num_letters	3.20	[0.28, 6.14]	1.49	1.00	19447	22614
T_N	2.29	[-0.44, 5.01]	1.39	1.00	19692	23621
T_abs_tok_MBOF	1.56	[-0.58, 3.72]	1.10	1.00	20231	23162
Trial	-2.66	[-3.66, -1.65]	0.51	1.00	80914	23789
PrevRT_man	10.43	[9.37, 11.49]	0.54	1.00	74895	24202
PrevERR	-2.09	[-4.29, 0.14]	1.14	1.00	77453	24262
Relatedness	-10.12	[-11.74, -8.50]	0.83	1.00	21610	24451
List	0.55	[-8.78, 9.81]	4.76	1.00	2947	5731
PEUBI_CT	-0.43	[-9.81, 8.99]	4.80	1.00	3730	7738
Relatedness:List	-2.24	[-4.42, -0.06]	1.11	1.00	19472	23191
Relatedness:PEUBI_CT	0.68	[-0.58, 1.93]	0.64	1.00	24248	26199
Random (group-level) effects – By-participant						
	Estimate	95% CrI	*SE*	$\hat{R}$	ESS	
					Bulk	Tail
sd(Intercept)	47.13	[40.81, 54.59]	3.51	1.00	4865	9176
sd(Relatedness)	4.13	[2.64, 5.65]	0.76	1.00	13823	18611
cor(Intercept,Relatedness)	-0.42	[-0.69, -0.12]	0.15	1.00	26528	19885
Random (group-level) effects – By-target						
	Estimate	95% CrI	*SE*	$\hat{R}$	ESS	
					Bulk	Tail
sd(Intercept)	11.85	[10.21, 13.66]	0.88	1.00	13908	21389
sd(Relatedness)	6.95	[5.46, 8.50]	0.77	1.00	14437	20546
sd(PEUBI_CT)	0.98	[0.04, 2.65]	0.72	1.00	12239	16930
cor(Intercept,Relatedness)	-0.15	[-0.40, 0.12]	0.13	1.00	13199	21284
cor(Intercept,PEUBI_CT)	-0.11	[-0.76, 0.64]	0.36	1.00	49419	22010

cor(Relatedness,PEUBI_CT)	0.12	[-0.65, 0.77]	0.37	1.00	45746	23484
Family-specific parameters (Ex-Gaussian distribution)						
	Estimate	95% CrI	*SE*	$\hat{R}$	ESS	
					Bulk	Tail
sigma	35.93	[34.80, 37.07]	0.58	1.00	50138	25235
beta	92.73	[90.84, 94.64]	0.97	1.00	61415	27154

Note. **Model** = RT ~ 1 + P_AoA + P_Conc + P_Fam + P_Val + P_Aro + P_NLD_Spanish_Catalan + P_prevalence_nts + P_log_frq + P_num_letters + P_N + P_abs_tok_MBOF + T_AoA + T_Conc + T_Fam + T_Val + T_Aro + T_NLD_Spanish_Catalan + T_prevalence_nts + T_log_frq + T_num_letters + T_N + T_abs_tok_MBOF + Trial + PrevRT_man + PrevERR + Relatedness + List + PEUBI_CT + Relatedness:List + Relatedness:PEUBI_CT + (1 + Relatedness | Participant) + (1 + Relatedness + PEUBI_CT | Target); **P_AoA =** prime word Age of acquisition; **P_Conc =** prime word Concreteness; **P_Fam =** prime word Familiarity; **P_Val =** prime word Valence; **P_Aro =** prime word Arousal; **P_NLD_Spanish_Catalan =** prime word Normalised Levenshtein distance between Spanish–Catalan translations; **P_prevalence_nts =** prime word Prevalence; **P_log_frq =** prime word Frequency (logarithmic scale); **P_num_letters =** prime word Length (number of letters); **P_N =** prime word Number of orthographic neighbours; **P_abs_tok_MBOF =** prime word Bigram frequency (logarithmic scale); **T_AoA =** target word Age of acquisition; **T_Conc =** target word Concreteness; **T_Fam =** target word Familiarity; **T_Val =** target word Valence; **T_Aro =** target word Arousal; **T_NLD_Spanish_Catalan =** target word Normalised Levenshtein distance between Spanish–Catalan translations; **T_prevalence_nts =** target word Prevalence; **T_log_frq =** target word Frequency (logarithmic scale); **T_num_letters =** target word Length (number of letters); **T_N =** target word Number of orthographic neighbours; **T_abs_tok_MBOF =** target word Bigram frequency (logarithmic scale); **Trial** = Trial order; **PrevRT_man =** RT of the preceding trial; **PrevERR =** Response accuracy of the preceding trial (sum-coded as *−1 = incorrect* and *+1 = correct*); **Relatedness =** Prime-target relatedness (sum-coded as *−1 = unrelated* and *+1 = related*); **List** (sum-coded as *−1 = list B* and *+1 = list A*); **PEUBI_CT** = conspiracy

theories (EUB score); $\hat{\boldsymbol{R}}$ = Gelman-Rubin convergence diagnostic; **ESS =** effective sample size; **sd() =** standard deviation for the random effect inside the parenthesis; **cor() =** correlation between the random effects inside the parenthesis.

Figure S3.5

Posterior distributions of fixed effects with 95% CrI in red (PEUBI-CT as EUB score, RTs as dependant variable)

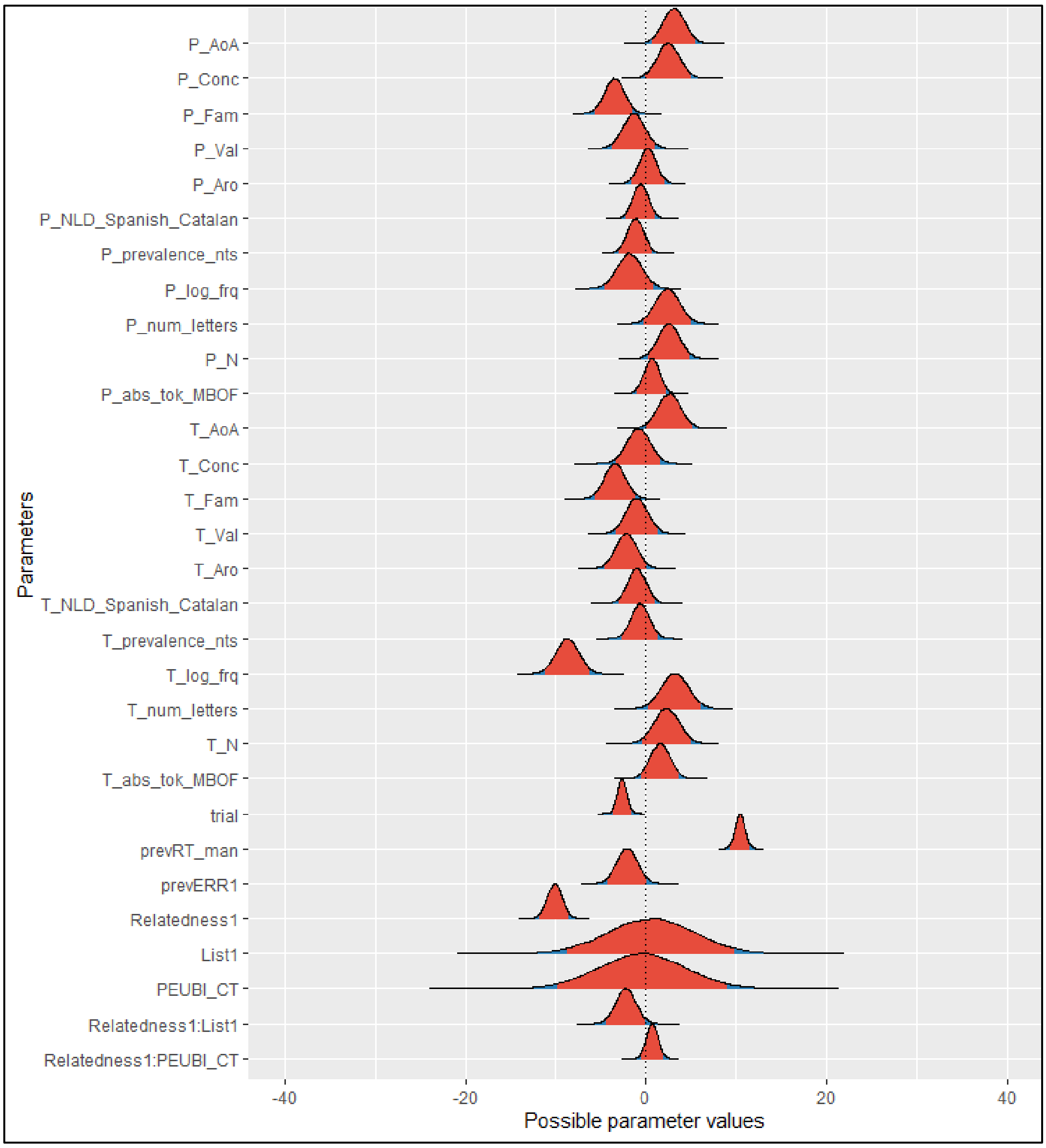

Note. **P_AoA** = prime word Age of acquisition; **P_Conc** = prime word Concreteness; **P_Fam** = prime word Familiarity; **P_Val** = prime word Valence; **P_Aro** = prime word Arousal; **P_NLD_Spanish_Catalan** = prime word Normalised Levenshtein distance between Spanish–Catalan translations; **P_prevalence_nts** = prime word Prevalence; **P_log_frq** = prime word Frequency (logarithmic scale); **P_num_letters** = prime word Length (number of letters); **P_N** = prime word Number of orthographic neighbours; **P_abs_tok_MBOF** = prime word Bigram frequency (logarithmic scale);

T_AoA = target word Age of acquisition; **T_Conc =** target word Concreteness; **T_Fam =** target word Familiarity; **T_Val =** target word Valence; **T_Aro =** target word Arousal; **T_NLD_Spanish_Catalan =** target word Normalised Levenshtein distance between Spanish–Catalan translations; **T_prevalence_nts =** target word Prevalence; **T_log_frq =** target word Frequency (logarithmic scale); **T_num_letters =** target word Length (number of letters); **T_N =** target word Number of orthographic neighbours; **T_abs_tok_MBOF =** target word Bigram frequency (logarithmic scale); **Trial** = Trial order; **PrevRT_man =** RT of the preceding trial; **PrevERR =** Response accuracy of the preceding trial (sum-coded as *−1 = incorrect* and *+1 = correct*); **Relatedness =** Prime-target relatedness (sum-coded as *−1 = unrelated* and *+1 = related*); **List** (sum-coded as *−1 = list B* and *+1 = list A*); **PEUBI_CT** = conspiracy theories (EUB score).

Table S3.6

Bayesian LMEM summary (PSEUDO-R as EUB score, RTs as dependant variable)

Fixed (population-level) effects						
	Estimate	95% CrI	*SE*	$\hat{R}$	ESS	
					Bulk	Tail
Intercept	534.36	[524.41, 544.28]	5.06	1.00	1776	4046
P_AoA	3.10	[0.73, 5.47]	1.21	1.00	19622	23774
P_Conc	2.48	[-0.00, 4.94]	1.26	1.00	15521	20756
P_Fam	-3.49	[-5.59, -1.38]	1.08	1.00	20052	23047
P_Val	-1.38	[-3.72, 1.00]	1.21	1.00	16972	21431
P_Aro	0.21	[-1.65, 2.07]	0.95	1.00	20943	21951
P_NLD_Spanish_Catalan	-0.58	[-2.27, 1.10]	0.86	1.00	23341	24065
P_prevalence_nts	-1.16	[-2.97, 0.67]	0.93	1.00	21804	22982
P_log_frq	-1.80	[-4.46, 0.89]	1.38	1.00	15027	21053
P_num_letters	2.35	[-0.27, 4.94]	1.32	1.00	15638	20625
P_N	2.53	[0.18, 4.88]	1.20	1.00	17748	21669
P_abs_tok_MBOF	0.66	[-1.03, 2.35]	0.86	1.00	21531	22439
T_AoA	2.56	[0.09, 5.08]	1.27	1.00	16063	20564
T_Conc	-0.83	[-3.28, 1.66]	1.27	1.00	15962	20547
T_Fam	-3.47	[-5.70, -1.22]	1.15	1.00	16335	19906
T_Val	-0.99	[-3.35, 1.36]	1.21	1.00	16551	21582
T_Aro	-2.18	[-4.50, 0.12]	1.17	1.00	15947	19132
T_NLD_Spanish_Catalan	-1.02	[-3.09, 1.04]	1.05	1.00	16700	19647
T_prevalence_nts	-0.64	[-2.62, 1.34]	1.01	1.00	16457	21421
T_log_frq	-8.71	[-11.19, -6.21]	1.27	1.00	15022	21415

T_num_letters	3.21	[0.30, 6.16]	1.50	1.00	14619	20886
T_N	2.28	[-0.46, 5.03]	1.40	1.00	14660	19498
T_abs_tok_MBOF	1.56	[-0.57, 3.71]	1.09	1.00	15784	19922
Trial	-2.67	[-3.66, -1.67]	0.51	1.00	60015	24733
PrevRT_man	10.42	[9.36, 11.48]	0.54	1.00	50699	22474
PrevERR	-2.11	[-4.36, 0.13]	1.14	1.00	55839	24018
Relatedness	-10.13	[-11.74, -8.52]	0.82	1.00	19997	22616
List	0.64	[-8.81, 9.90]	4.73	1.00	2426	4468
PSEUDO_R	-7.02	[-16.35, 2.32]	4.75	1.00	2647	4968
Relatedness:List	-2.26	[-4.45, -0.08]	1.12	1.00	16017	20030
Relatedness:PSEUDO_R	1.41	[0.16, 2.66]	0.63	1.00	19921	20459
Random (group-level) effects – By-participant						
	Estimate	95% CrI	*SE*	$\hat{R}$	ESS	
					Bulk	Tail
sd(Intercept)	46.52	[40.32, 53.84]	3.45	1.00	3586	6877
sd(Relatedness)	3.90	[2.34, 5.43]	0.78	1.00	11056	13217
cor(Intercept,Relatedness)	-0.40	[-0.69, -0.08]	0.15	1.00	21517	19354
Random (group-level) effects – By-target						
	Estimate	95% CrI	*SE*	$\hat{R}$	ESS	
					Bulk	Tail
sd(Intercept)	11.83	[10.20, 13.61]	0.87	1.00	12731	20365
sd(Relatedness)	6.93	[5.44, 8.47]	0.77	1.00	13154	19409
sd(PSEUDO_R)	0.97	[0.04, 2.70]	0.73	1.00	10411	14241
cor(Intercept,Relatedness)	-0.15	[-0.40, 0.12]	0.13	1.00	11002	19355
cor(Intercept,PSEUDO_R)	-0.09	[-0.75, 0.66]	0.37	1.00	39946	20174

cor(Relatedness,PSEUDO_R)	0.09	[-0.67, 0.76]	0.37	1.00	38813	22838
Family-specific parameters (Ex-Gaussian distribution)						
	Estimate	95% CrI	*SE*	$\hat{R}$	ESS	
					Bulk	Tail
sigma	35.95	[34.81, 37.10]	0.58	1.00	40631	25158
beta	92.70	[90.82, 94.60]	0.97	1.00	47484	26063

Note. **Model** = RT ~ 1 + P_AoA + P_Conc + P_Fam + P_Val + P_Aro + P_NLD_Spanish_Catalan + P_prevalence_nts + P_log_frq + P_num_letters + P_N + P_abs_tok_MBOF + T_AoA + T_Conc + T_Fam + T_Val + T_Aro + T_NLD_Spanish_Catalan + T_prevalence_nts + T_log_frq + T_num_letters + T_N + T_abs_tok_MBOF + Trial + PrevRT_man + PrevERR + Relatedness + List + PSEUDO_R + Relatedness:List + Relatedness:PSEUDO_R + (1 + Relatedness | Participant) + (1 + Relatedness + PSEUDO_R | Target); **P_AoA =** prime word Age of acquisition; **P_Conc =** prime word Concreteness; **P_Fam =** prime word Familiarity; **P_Val =** prime word Valence; **P_Aro =** prime word Arousal; **P_NLD_Spanish_Catalan =** prime word Normalised Levenshtein distance between Spanish–Catalan translations; **P_prevalence_nts =** prime word Prevalence; **P_log_frq =** prime word Frequency (logarithmic scale); **P_num_letters =** prime word Length (number of letters); **P_N =** prime word Number of orthographic neighbours; **P_abs_tok_MBOF =** prime word Bigram frequency (logarithmic scale); **T_AoA =** target word Age of acquisition; **T_Conc =** target word Concreteness; **T_Fam =** target word Familiarity; **T_Val =** target word Valence; **T_Aro =** target word Arousal; **T_NLD_Spanish_Catalan =** target word Normalised Levenshtein distance between Spanish–Catalan translations; **T_prevalence_nts =** target word Prevalence; **T_log_frq =** target word Frequency (logarithmic scale); **T_num_letters =** target word Length (number of letters); **T_N =** target word Number of orthographic neighbours; **T_abs_tok_MBOF =** target word Bigram frequency (logarithmic scale); **Trial** = Trial order; **PrevRT_man =** RT of the preceding trial; **PrevERR =** Response accuracy of the preceding trial (sum-coded as *−1 = incorrect* and *+1 = correct*); **Relatedness =** Prime-target relatedness (sum-coded as *−1 = unrelated* and *+1 = related*); **List** (sum-coded as *−1 = list B* and *+1 = list A*); **PSEUDO_R =**

pseudoscience (EUB score); $\hat{\boldsymbol{R}}$ = Gelman-Rubin convergence diagnostic; **ESS =** effective sample size; **sd() =** standard deviation for the random effect inside the parenthesis; **cor() =** correlation between the random effects inside the parenthesis.

Figure S3.6

Posterior distributions of fixed effects with 95% CrI in red (PSEUDO-R as EUB score, RTs as dependant variable)

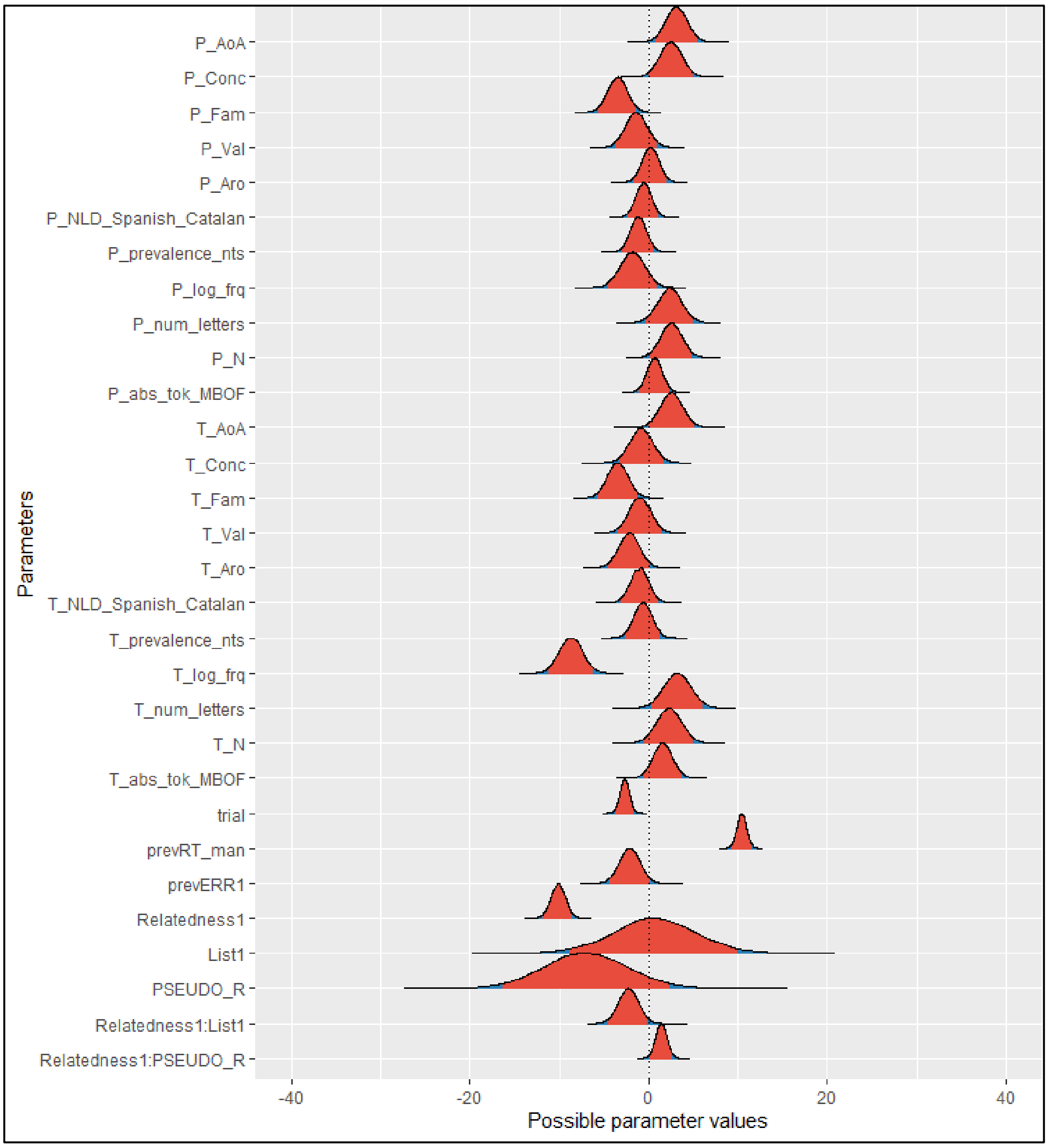

Note. **P_AoA** = prime word Age of acquisition; **P_Conc** = prime word Concreteness; **P_Fam** = prime word Familiarity; **P_Val** = prime word Valence; **P_Aro** = prime word Arousal; **P_NLD_Spanish_Catalan** = prime word Normalised Levenshtein distance between Spanish–Catalan translations; **P_prevalence_nts** = prime word Prevalence; **P_log_frq** = prime word Frequency (logarithmic scale); **P_num_letters** = prime word Length (number of letters); **P_N** = prime word Number of orthographic neighbours; **P_abs_tok_MBOF** = prime word Bigram frequency (logarithmic scale);

T_AoA = target word Age of acquisition; **T_Conc =** target word Concreteness; **T_Fam =** target word Familiarity; **T_Val =** target word Valence; **T_Aro =** target word Arousal; **T_NLD_Spanish_Catalan =** target word Normalised Levenshtein distance between Spanish–Catalan translations; **T_prevalence_nts =** target word Prevalence; **T_log_frq =** target word Frequency (logarithmic scale); **T_num_letters =** target word Length (number of letters); **T_N =** target word Number of orthographic neighbours; **T_abs_tok_MBOF =** target word Bigram frequency (logarithmic scale); **Trial** = Trial order; **PrevRT_man =** RT of the preceding trial; **PrevERR =** Response accuracy of the preceding trial (sum-coded as *−1 = incorrect* and *+1 = correct*); **Relatedness =** Prime-target relatedness (sum-coded as *−1 = unrelated* and *+1 = related*); **List** (sum-coded as *−1 = list B* and *+1 = list A*); **PSEUDO_R** = pseudoscience (EUB score).
