# Supplementary material for: Individual differences in associative/semantic priming: Spreading of activation in semantic memory and epistemically unwarranted beliefs
Source: PLoS One. 2025 Feb 11;20(2):e0313239. doi: 10.1371/journal.pone.0313239 (PMC11813106; doi:10.1371/journal.pone.0313239)
Supplement: S4 File — Refer to this document for references from S4.1 to S4.10. (PDF) [file pone.0313239.s004.pdf]

Individual differences in associative/semantic priming: Spreading of activation in semantic memory and epistemically unwarranted beliefs – SUPPLEMENTARY MATERIAL: Endnotes

Endnote 1. This conceptualization of semantic memory is not shared by all models. For instance, distributed feature-based network accounts consider that the nodes of the semantic network are not concepts, but binary features/characteristics. According to these models, a particular concept (e.g., penguin) would reside in a specific activation pattern involving its defining features (e.g., has wings, is white, is black, can't fly, does swim, does waddle…), that is, would emerge from integrating this set of activated characteristics [1, 2 Chapter five].

Endnote 2. Traditionally, the literature of semantic memory has distinguished two types of relationships between concepts: associative (i.e., based on co-occurrence, such as free-association norms) and semantic (i.e., based on shared semantic features, such as exemplars of the same semantic category and synonyms). However, these two types of relationships are usually intermixed and can be difficult to differentiate (see [1, 2 Chapter ten]). This is the reason why the inclusive term 'associative/semantic' was used throughout this work.

Endnote 3. The degree of lexical overlap between Spanish-Catalan translations (i.e., cognate status; see [3]) was controlled because our participants generally were bilinguals of these two languages.

Endnote 4. Bayesian independent samples t-tests' was always calculated with JASP's default prior distribution (Cauchy distribution, location = 0, scale = 0.707).

Endnote 5. The abbreviation CrI is adopted for referring to Bayesian credible interval, to differentiate it from the CI abbreviation conventionally used for referring to frequentist confidence interval.

Endnote 6. Although not reported here, frequentist versions of the LMEM analyses were also performed to explore the sensitivity of results to analytic approach, using *lme4* (version 1.1.35.1; [4]) and *lmerTest* (version 3.1.3; [5]) libraries.

Endnote 7. Relatedness predictor was sum-coded as *−1 = unrelated* and *+1 = related*. Following Brehm & Alday [6], in this case the estimate for this parameter represents "half the change in the y value between the two levels" (p. 2), moving from the negatively-coded level to the positively-coded level. Therefore, an estimate of $b = -10$ would represent a change of −20 ms moving from the unrelated condition to the related condition. Consider this rationale when interpreting the magnitude of any two-level categorical predictor (i.e., Relatedness, List, Previous error).

Endnote 8. Since the term 'marginal effects' can differ in meaning across studies and analyses, check the specific instructions used to generate the plots in *S1. Data analysis details* document at Supporting Information files.

Endnote 9. It must be noted that, when constructing the two lists, related pairs were distributed as evenly as possible in terms of FSG. Indeed, independent samples t-tests were performed to check that mean FSG did not significantly differ between lists (p = .939, 95% CI for means difference [-0.03, 0.03], BF_{01} = 6.48, 95% CrI for effect size [-0.26, 0.28]), and two-sample Kolmogorov-Smirnov tests were performed to check that FSG distributions did not significantly differ between lists (p = 1.000).

Endnote 10. Although it was not the purpose of the present research, our results also add evidence to the vast literature regarding the effects of word properties over visual word recognition (see *Control predictors* Results' subsection; e.g., [7, 8, 9]). Future studies could further explore the effects of prime word properties over primed LDT RTs, which is comparatively less studied than the effects of target word properties

References

1. Kumar AA. Semantic memory: A review of methods, models, and current challenges. Psychon Bull Rev. 2021 Feb;28(1):40-80. doi: 10.3758/s13423-020-01792-x.

2. McNamara TP. Semantic priming: Perspectives from memory and word recognition. Psychology Press; 2005.

3. Guasch M, Boada R, Ferré P, Sánchez-Casas R. NIM: A Web-based Swiss army knife to select stimuli for psycholinguistic studies. Behav Res Methods. 2013 Sep;45(3):765-71. doi: 10.3758/s13428-012-0296-8.

4. Bates D, Mächler M, Bolker BM, Walker SC. Fitting linear mixed-effects models using lme4. J Stat Softw. 2015;67(1):1-48. doi: 10.18637/jss.v067.i01.

5. Kuznetsova A, Brockhoff PB, Christensen RHB. lmerTest package: Tests in linear mixed effects models. J Stat Softw. 2017;82(13):1-26. doi: 10.18637/jss.v082.i13.

6. Brehm L, Alday PM. Contrast coding choices in a decade of mixed models. J Mem Lang. 2022 Aug;125:104334. doi: 10.1016/j.jml.2022.104334.

7. Adelman JS. Methodological issues with words. In: Adelman JS, editor. Visual word recognition (vol. 1): Models and methods, orthography and phonology. Psychology Press; 2012. pp. 116-38.

8. Pexman PM. Meaning-based influences on visual word recognition. In: Adelman JS, editor. Visual word recognition (vol. 2): Meaning and context, individuals and development. Psychology Press; 2012. pp. 24-43.

9. Yap MJ, Balota DA. Visual word recognition. In: Pollatsek A, Treiman R, editors. The Oxford handbook of reading. Oxford University Press; 2015. pp. 26-43.
