# Supplementary material for: Correction: Individual differences in associative/semantic priming: Spreading of activation in semantic memory and epistemically unwarranted beliefs
Source: PLoS One. 2025 Aug 18;20(8):e0330531. doi: 10.1371/journal.pone.0330531 (PMC12360512; doi:10.1371/journal.pone.0330531)
Supplement: S2 File — (S2_File.PDF) [file pone.0330531.s002.pdf]

PLOS ONE

RESEARCH ARTICLE

Individual differences in associative/semantic priming: Spreading of activation in semantic memory and epistemically unwarranted beliefs

Daniel Huete-Pérez[1]*, **Robert Davies**[2], **Javier Rodríguez-Ferreiro**[3], **Pilar Ferré**[1]

1 Universitat Rovira i Virgili, Department of Psychology, Research Center for Behavior Assessment (CRAMC), Tarragona, Spain, **2** Department of Psychology, Lancaster University, Bailrigg, Lancaster, United Kingdom, **3** Grup de Recerca en Cognició i Llenguatge (GRECIL), Departament de Cognició, Desenvolupament i Psicologia de la Educació, Secció de Processos Cognitius, Institut de Neurociències (INUB), Universitat de Barcelona (UB), Barcelona, Spain

* daniel.huete@urv.cat

Check for updates

OPEN ACCESS

Citation: Huete-Pérez D, Davies R, Rodríguez-Ferreiro J, Ferré P (2025) Individual differences in associative/semantic priming: Spreading of activation in semantic memory and epistemically unwarranted beliefs. PLoS One 20(2): e0313239. https://doi.org/10.1371/journal.pone.0313239

Editor: Muhammad Shahzad Aslam, Xiamen University - Malaysia Campus: Xiamen University - Malaysia, MALAYSIA

Received: April 26, 2024

Accepted: October 21, 2024

Published: February 11, 2025

Copyright: © 2025 Huete-Pérez et al. This is an open access article distributed under the terms of the Creative Commons Attribution License, which permits unrestricted use, distribution, and reproduction in any medium, provided the original author and source are credited.

Data availability statement: Data, analysis code (R scripts) and fitted models (Bayesian LMEMs) that support the findings of this study are openly available in Open Science Framework (OSF) repository at https://doi.org/10.17605/OSF.IO/G62ST. Data analysis details document (more detailed account of our analytical and technical decisions, along with

Abstract

Starting from the *enhanced spreading of activation through semantic memory* (one of the explanatory mechanisms attempting to explain some manifestations observed in schizophrenia) and the *psychosis continuum* (a dimensional approach to psychotic disorders, where 'normality' and 'psychopathology' are not qualitatively different in nature but placed on varying levels of the same continuum), the main aim of the present research was to explore whether there are individual differences in associative/semantic priming in people with different levels of epistemically unwarranted beliefs (EUB). Participants varying in paranormal, pseudoscientific and conspiracy endorsement completed a primed lexical decision task containing related prime-target words (e.g., bulb-light) and unrelated prime-target words (e.g., sock-light). Bayesian linear mixed-effects models over response times (RTs) revealed a main direct priming effect (faster RTs in related pairs than in unrelated ones), a main facilitatory effect for some EUB scores (i.e., the higher the value for EUB score, the faster RTs), and an interactive effect between the experimental manipulation and some EUB scores (the higher the EUB score, the smaller the direct priming effect). These results are consistent with predictions made from the enhanced spreading of activation explanatory mechanism, but other alternative accounts are also discussed.

Introduction

Schizophrenia is a diagnostic label used to designate a heterogenous combination of symptoms in perception (e.g., hallucinations, delusions), language and communication (e.g., disorganized speech, poverty of speech), affect (e.g., anhedonia, amotivation, flat emotional expression), and social behaviour (e.g., disorganized behaviour, social withdrawal), which cannot be attributed to other causes (e.g., direct effects of a drug) and that significantly impair a person's

relevant code) and LMEMs complete reporting document (full results for each model with all relevant estimates) are available as Supporting Information files.

Funding: This work was supported by Spanish Ministerio de Universidades (DHP's predoctoral contract FPU20/03345 from the call Ayudas para la formación de profesorado universitario – Convocatoria 2020 + DHP's mobility grant EST23/00260 from the call Ayudas complementarias de movilidad destinadas a beneficiarios del programa de formación del profesorado universitario – Convocatoria 2023). Open access Article Processing Charges (APC) were paid with funds from the Psychology Department of Universitat Rovira i Virgili. The funders had no role in study design, data collection and analysis, decision to publish, or preparation of the manuscript.

Competing interests: The authors have declared that no competing interests exist.

everyday functioning [1–3]. From the very beginning, the term *loosening of associations* has been used to describe one of the key underlying disturbances in schizophrenia [4], that is, a disruption in how concepts, thoughts and pieces of information are connected in a logical, warranted, and coherent way [5]. This associative disturbance could explain some manifestations of schizophrenia, such as disorganised speech (i.e., jumping incoherently from one word or idea to another) and delusions (i.e., associating pieces of information that are, in fact, not connected). Evidence supporting this loosening of associations in schizophrenia comes from observations including derailment and tangentiality in speech (see [6]), production of infrequent words in verbal fluency tasks (e.g., [7]) and word-association tests (e.g., [8]), and increased associative/semantic priming effects (e.g., [9]), among others.

One of the main explanatory mechanisms that attempts to account for the loosening of associations is the *enhanced spreading of activation through semantic memory* (e.g., [6,10–12]). According to localist network accounts, semantic memory can be understood as a network of interconnected concept nodes (S4.1 in S4 File) (e.g., [13]). When one node is activated (e.g., by thinking about the concept or reading the word that denominates it), part of its activation spreads through its links to other nodes [14, 15 Chapter two]. Nodes reached by this spread activation will be pre-activated to some extent and, accepting that activation level is what determines memory accessibility [16], these nodes' concepts would be more easily evoked. Of note, this activation is not propagated equally but depends on the characteristics of the connections such as directedness or distance (directly linked, linked through one intermediate node, linked through two intermediate nodes, etc.) and strength (some relationships are stronger than others) [14,15 Chapter eleven]. These characteristics, in turn, depend on factors like the degree of lexical co-occurrence and the amount of shared semantic features [6,17]. In this context, people diagnosed with schizophrenia may show loose associations because their semantic memory networks would exhibit an enhanced spreading of activation (e.g., [6,10–12]) in comparison to people without such a diagnosis. This would lead to co-activation of nodes that are distantly or weakly related.

Experimental evidence for the loosening of association phenomenon has also been obtained in non-clinical individuals scoring high in schizotypal personality traits (see [10]), with results analogous to those observed in schizophrenia. For instance, Kiang and Kutas [18] observed that schizotypal personality scores were positively correlated with an index on the atypicality of words produced in a verbal fluency task. This commonality with loosening of associations in schizophrenia is not entirely surprising following a dimensional perspective of psychopathology, where 'normality' and 'psychopathology' are not qualitatively different in nature but placed on varying levels of the same continuum (e.g., [19,20]; see also [21] for an example of dimensional nosology of psychopathology). Indeed, several proposals exist about a *psychosis continuum* where schizotypal personality can be accommodated (for an overview, see [22,23]). The psychosis continuum framework opens the possibility of studying schizophrenia-related issues in non-clinical populations: instead of comparing people diagnosed with schizophrenia with people without such diagnosis, these issues can be indirectly studied by examining individuals with varying levels of schizotypal or other related traits. This approach makes it possible to avoid some problems associated with studies with people diagnosed with schizophrenia, such as difficulty in finding and recruiting participants, and the confounding effects of variables like primary and secondary effects of medication [10,24].

Starting from the psychosis continuum framework, the present investigation focuses on the extent to which loosening of associations can be observed in non-clinical individuals during semantic processing, in particular, in an associative/semantic priming paradigm. A typical trial of this paradigm involves the sequential presentation of a pair of stimuli: the first is the *prime*, the second is the *target*. While the prime stimulus only needs to be attended, participants are

usually required to perform a task with regards to the target stimulus, such as lexical decision (LDT; to decide whether it is a real word or a string of letters that does not correspond to an existing word) and naming (to read it aloud) [15 Chapter one]. The *associative/semantic priming effect* refers to the consistently observed result that response times (RTs) and/or response accuracy for the target word (e.g., water) are facilitated when the prime word is associatively or semantically related (S4.2 in S4 File) (e.g., glass) in comparison to when it is unrelated (e.g. class). This effect can be understood as a consequence of the by-default functioning of semantic memory: the evocation of a target word is facilitated when preceded by a related prime (but not by an unrelated prime) because its concept node would be pre-activated, as a consequence of the spreading activation coming from the concept node of the prime word ([15] Chapter two, [25]). It should be mentioned that associative/semantic priming effects have been attributed not only to automatic processes (e.g., spreading activation), but also to controlled and strategic processes (e.g., expectancy, semantic matching) (for more details, see [15 Chapter nine, 26]).

Our study is motivated by inconsistencies in the evidence regarding associative/semantic priming effects in individuals diagnosed with schizophrenia or with high levels of schizotypal traits: exacerbated priming effects (hyperpriming); diminished priming effects (hypopriming); and non-significant differences have been reported in comparison to their respective control groups (for overviews, see [10,12,17]). These inconsistencies can be explained, in part, by methodological differences between studies regarding how the experimental priming paradigm has been implemented (see [10,12,17]). A first experimental variable that can modulate associative/semantic priming effects is the directness of relationship: prime-target words can be directly related (i.e., glass-water) or they can be indirectly related through one or more intermediate word/s (i.e., plate-water through glass) [15 Chapter eleven, 26]. Indirect priming seems to be more difficult to detect than direct priming in behavioural measures [27]. A second experimental variable that can modulate associative/semantic priming effects is stimulus-onset asynchrony (SOA; time between the beginning of the prime word and the beginning of the target word): short SOAs have been associated mainly with automatic processes, while the longer the SOA is, the more likely that controlled and strategic processes come into play [15 Chapter nine, 26]. A third experimental variable that can modulate associative/semantic priming effects is relatedness proportion (proportion of related prime-target trials out of the total word-word trials): especially in long SOAs, priming tends to increase in absolute size as the relatedness proportion increases [15 Chapter nine, 28].

Rodríguez-Ferreiro et al. [12] identified other methodological issues that may contribute to the between-studies variation in findings. Firstly, the comparison of categorical groups (e.g., high schizotypy vs. low schizotypy) instead of keeping the variable continuous (e.g., schizotypy score) usually leads to decreased statistical power to detect effects [12]. Secondly, the usage of difference scores as the dependent variable (priming effect = mean RT of the unrelated prime condition – mean RT of the related prime condition) may increase statistical noise [12]. Moreover, when these difference scores are the only measures reported (i.e., no RTs per condition), the amount of information provided is importantly reduced: for instance, a larger priming (i.e., greater RT difference between the unrelated and related conditions) can be due to both a reduction of RTs in the related condition and an increase of RTs in the unrelated condition [15 Chapter eight]. Thirdly, the traditional analytical approach in psycholinguistics has been to conduct separated by-participant (collapsing/averaging over items) and by-item (collapsing/averaging over participants) statistical analysis, as in ANOVA and linear regression. However, this statistical approach has several limitations, such as increased rates of faulty statistical inference (i.e., both false positives and false negatives; see [29–31]). Fortunately, better alternatives are now available: linear mixed-effects models (LMEMs; for an accessible introduction, see [30]). Finally, literature inconsistencies for

associative/semantic priming in schizophrenia and schizotypy could also be due to *within-diagnostic heterogeneity*, one of the intrinsic problems associated to categoric nosological systems. Indeed, two individuals diagnosed with schizophrenia can be very different in their symptomatology and underlying psychopathological mechanisms [32,33]. Therefore, it is possible that some individuals diagnosed with schizophrenia have a semantic network with enhanced spreading activation (e.g., those with predominantly positive symptoms, such as delusions), while others do not (e.g., those with predominantly negative symptoms, such as anhedonia). A similar logic can be applied to high-schizotypy individuals, since the underlying structure of manifestations of this construct is similar to schizophrenia [23]. To overcome this limitation, instead of starting from generic categoric labels (e.g., schizophrenia, schizotypy) or symptoms clusters (e.g., positive, negative or disorganized symptoms), one possibility is to focus on specific traits (e.g., unusual beliefs, [32]) which may be specifically related with the cognitive processes which are the focus of this research (spreading of activation through semantic networks).

The present study

Epistemically unwarranted beliefs (EUB; [34]) is a term used to encompass beliefs that are not logically or empirically grounded [35], being paranormal phenomena (e.g., certain numbers giving good/back luck), pseudoscientific speculations and practices (e.g., lie detection through polygraph), and conspiracy theories (e.g., COVID-19 'plandemic´) three popular instances in contemporary societies (see [34,36]). In a similar vein to schizotypal personality traits, there is evidence that EUB may be placed within the psychosis continuum (e.g., see [37–39]). Therefore, we might expect people with high levels of EUB to have some similarities with people diagnosed with schizophrenia, like an enhanced spreading activation in semantic networks, which would reflected in the associative/semantic priming paradigm.

To the best of our knowledge, there are no studies directly evaluating this hypothesis in relation to pseudoscientific or conspiracy beliefs, although there are a few preceding associative/semantic priming studies in relation to paranormal and magical beliefs [40–42].These three studies have in common that at least two groups of non-clinical individuals (high paranormal/magical beliefs vs. low paranormal/magical beliefs) performed an associative/semantic priming experimental procedure which contained related and unrelated prime-target word pairs. Nevertheless, they also differed in several aspects: how related pairs were defined (category-associated vs. category-unassociated vs. function-associated vs. function-unassociated in [40]; directly related vs. indirectly related in [41,42]); which was the main task (naming [40] vs. lexical decision [41,42]); or the inclusion of additional independent variables (e.g., stimulus lateralized presentation [41,42], substance administration [42]). Kerns and Berenbaum [40] found a significant main effect of prime-target relationship ("overall semantic priming" [40, p. 729] effect), with faster RTs to related than to unrelated prime-target pairs. Unfortunately, it is unclear whether there is a significant main effect of group or not, and a direct test of the interactive effect group x prime-target relationship is not available. However, groups were compared on the size of the priming effect, which can be understood as an approximation of the interactive effect of interest (but keep in mind that, given the aforementioned issues of using difference scores [12], a direct test of the interaction over raw RTs would be needed). This analysis revealed that believers showed a significantly increased priming effect in comparison to non-believers "for all prime conditions" and "averaging across all prime conditions" [40, p. 729]. Pizzagalli et al. [41], also found a significant main effect of prime-target relationship (directly related pairs had faster RTs than indirectly related pairs and unrelated pairs, and, in turn, indirectly related pairs had faster RTs than unrelated

pairs). However, the main effect of group was not significant. Moreover, a triple interaction was found between the independent variables of the study: paranormal believers and disbelievers only differed in indirectly related pairs when they were presented in the left visual field, with faster RTs for believers. Mohr et al. [42] had a complex design with four independent variables. Focusing on the analyses carried out with the placebo group (results without the interference of any psychoactive substance) and which collapsed the distinction of lateralized presentation (which is not of interest) [42, p. 78], the significant main effect of prime-target relationship was found again (directly related pairs had faster RTs than indirectly related pairs and unrelated pairs, and, in turn, indirectly related pairs had faster RTs than unrelated pairs). Additionally, a significant main effect of group was also found (believers produced faster RTs than non-believers). No interaction was found between these two variables.

Taking the three studies together [40–42], the only systematic result is the typical priming effect: faster RTs were produced to related pairs than to unrelated ones. However, they are not consistent with each other either in the main effect of group (believers with faster RTs than non-believers in [42], but without significant differences in [41]) or the group x prime-target relationship interactive effect (believers had an overall larger priming effect than non-believers in [40], believers had a stronger indirect –but not direct– priming effect than non-believers in [41], without significant differences in priming effects between believers and non-believers in [42]). In this context, our main aim was to explore whether there are individual differences in associative/semantic priming depending on participants' EUB level. With this purpose in mind, participants whose EUB levels were measured through self-report psychometric instruments performed a primed LDT. With this study we intended to contribute to resolving the inconsistencies in the literature regarding associative/semantic priming effects with respect to the psychosis continuum. Apart from focusing on a psychosis-related specific trait (i.e., unusual beliefs) to circumvent the within-diagnostic heterogeneity limitation, the methodological considerations and limitations previously identified were also taken into account in our study, in order to both examine the desired processes (i.e., automatic spreading activation) and to maximise statistical power. Firstly, we focused on direct rather than indirect priming: directly related and unrelated prime-target pairs were tested (from here on, this experimental manipulation will be referred to as Relatedness). Secondly, we used a short SOA (200 ms) stimuli presentation. Thirdly, the relatedness proportion was 50% (following [26]). Fourthly, individual differences in EUB were kept as a continuous variable (instead of dichotomizing to high vs. low groups). Fifthly, we did not focus on difference scores (i.e., the analysed data were raw RTs for related and unrelated prime-target pairs). Finally, we analysed our data through LMEMs (instead of conducting separate by-participant and by-item analyses).

We expected to replicate the typical direct priming effect, that is, a main effect of Relatedness with faster RTs for related prime-target pairs than for unrelated prime-target pairs. More importantly for our concerns, we hypothesised that if EUB believers generally experience a faster/greater and further reaching spreading of activation through semantic memory (e.g., [6,11]) compared to people with low levels of EUB, a main facilitatory effect of EUB would be expected. That is, high scores in EUB should be associated with shorter RTs in both related (faster/greater spreading to close associates; see [10]) and unrelated (further reaching spreading, which results in the activation of remote associates; see [10]) conditions. We supposed that, alternatively, if EUB facilitatory effects only occur in one of these two conditions (exclusively in related pairs or in unrelated pairs), that would suggest that only one of two possible mechanisms are in place: faster/greater spreading to close associates or further reaching spreading activating remote associates. This would be indicated by an interaction between EUB and Relatedness.

Method

Participants

Ninety-nine undergraduate Psychology students from Universitat Rovira i Virgili (URV, Tarragona, Spain) participated voluntarily (convenience sampling) in exchange for extra academic credits. One participant was removed from data analysis because that person exceeded the error rate limit (25%). The 98 valid participants were aged between 18-49 years (M = 19.61, SD = 3.65), with a sex distribution of 74 females and 24 males. The study was performed in accordance with the Declaration of Helsinki, and it was approved by the *Comitè Ètic d'Investigació en Persones*, *Societat i Medi Ambient* of URV (reference: CEIPSA-2021-TD-0023). Participants gave their informed written consent before starting the study.

Materials and instruments

Primed LDT. In the following, the psycholinguistic properties of the words used in this study (i.e., selected primes and targets) and their sources are listed: age of acquisition [43–45]; arousal [44,46–49]; bigram frequency (mean, token-absolute) [50] subtitle tokens database]; concreteness [44,46–48,50 subtitle tokens database]; contextual diversity (logarithmic scale) [50 subtitle contextual diversity database]; familiarity [44,45–47,50 subtitle tokens database]; Levenshtein distance between Spanish-Catalan translations (S4.3 in S4 File) [51]; mean Levenshtein distance of the 20 closest words [50 subtitle tokens database]; number of higher frequency orthographic neighbours [50 subtitle tokens database]; number of letters [50 subtitle tokens database]; number of orthographic neighbours [50 subtitle tokens database]; trigram frequency (mean, token-absolute) [50 subtitle tokens database]; valence [44,46–49]; word frequency (logarithmic scale) [50 subtitle tokens database]; and word prevalence (natives from Spain) [52]. Some of the normative values for some subjective variables (age of acquisition, concreteness, familiarity, valence, arousal) were accessed through EmoFinder [53].

Critical trials. 200 Spanish word triplets were selected. Each triplet contained a target word (e.g., *luz* [light]), and two possible prime words: one related to the target word (e.g., *bombilla* [bulb]) and another unrelated to it (e.g., *calcetín* [sock]). That is, there were two alternative prime-target pairings with the same target word (Relatedness: related vs. unrelated). We created two experimental lists (A vs. B) with one prime-target version in each list, so each participant only saw one prime-target version for each target word, either the related or the unrelated. This resulted in each list containing 100 related and 100 unrelated prime-target pairs.

Related prime-target pairs were obtained from the NALC free association norms [54–56]. The developers of the database provided us a file with all the cue-target pairs together with its forward associative strength (FSG, i.e., proportion of people producing the target word as the first thing that came into mind after being exposed to the cue word). We considered as candidate stimulus only those word pairs for which data were available for all the relevant word properties for both cue and target words. We ordered the list of candidates cue-target word pairs by descending FSG and selected those candidates for which the following restrictions were fulfilled: (1) no reference to proper nouns (e.g., *pantera-rosa* [Pink Panther]); (2) no word pairs that act as a single concept (e.g., *panda-oso* [panda bear]; (3) no shared lexeme between prime and target (e.g., *deshacer-hacer* [undo-do]); (4) no initial orthographic overlap between prime and target (e.g., *enojo-enfado* [annoyance-anger]; (5) no probably unknown words as primes; (6) no Spanish-Catalan false friends (e.g., *cama* [bed in Spanish, leg in Catalan]); and (7) no exact or very similar words in their lexical form to those already present in

Table 1. Descriptive statistics of properties of the words used in critical and filler trials.

	Critical trials									Filler trials		
	Related primes			Unrelated primes			Target words			Primes		
	M	SD	Range (min-max)	M	SD	Range (min-max)	M	SD	Range (min-max)	M	SD	Range (min-max)
Age of acquisition	6.75	1.92	1.74-10.68	6.82	1.97	2.42-10.78	4.63	1.63	1.12-9.98	6.76	1.95	2.32-10.60
Concreteness	4.93	0.97	1.99-6.63	4.90	0.96	2.39-6.68	5.07	1.01	2.15-6.72	4.89	0.93	2.35-6.64
Familiarity	5.33	0.92	2.74-6.84	5.30	1.01	2.44-6.88	6.18	0.60	3.15-7.00	5.31	0.94	2.32-7.00
Valence	5.49	1.40	1.20-8.60	5.52	1.37	1.45-8.25	5.83	1.60	1.45-8.70	5.42	1.34	1.35-8.35
Arousal	5.20	0.94	2.30-7.75	5.22	0.96	2.20-7.50	5.25	1.13	2.28-8.45	5.14	1.07	2.05-7.95
NLD Spanish-Catalan	0.71	0.29	0.00-1.00	0.71	0.28	0.00-1.00	0.64	0.30	0.00-1.00	0.71	0.27	0.00-1.00
Prevalence (in z-scores)	2.36	0.23	1.63-2.58	2.34	0.26	1.26-2.58	2.40	0.19	1.96-2.58	2.34	0.25	1.58-2.58
Word frequency (in logarithmic scale)	1.03	0.61	0.02-3.69	1.03	0.61	0.01-3.04	1.85	0.57	0.35-3.13	1.03	0.61	0.03-3.11
Length (in letters)	6.61	1.90	3-12	6.61	1.87	4-12	5.78	1.59	3-11	6.62	1.83	4-12
N	4.92	5.56	0-29	5.74	7.25	0-32	7.51	8.38	0-40	5.28	6.70	0-33
NHF	0.71	1.59	0-10	0.87	1.87	0-13	0.45	1.07	0-8	0.75	1.52	0-9
Lev_N	1.82	0.62	1.00-3.95	1.80	0.60	1.00-4.05	1.64	0.50	1.00-3.45	1.84	0.61	1.00-4.20
Bigram frequency (mean, token-absolute)	27124.38	11768.69	4234.40-66073.05	25408.93	10832.95	3057.30-61194.14	25729.68	11716.45	2099.43-64490.91	25892.75	11051.66	4986.46-55073.17
Trigram frequency (mean, token-absolute)	2723.67	2413.36	3.90-13668.18	2544.84	2304.73	57.96-12249.64	2488.85	2120.57	157.52-13647.86	2652.59	2517.68	23.16-15769.89
Contextual diversity (in logarithmic scale)	0.72	0.47	0.02-2.00	0.71	0.47	0.00-1.97	1.30	0.42	0.19-1.97	0.72	0.47	0.02-1.97

Note. NLD = normalised Levenshtein distance between Spanish–Catalan translations; N = orthographic neighbours; NHF = orthographic neighbours of higher frequency; Lev_N = mean Levenshtein distance of the 20 closest words.

https://doi.org/10.1371/journal.pone.0313239.t001

previously selected pairs. In all the selected pairs the target word was the first associate of the prime, with FSG values ranging from .43 to .94 (M = .56, SD = .11).

Unrelated primes were selected using the Match software [57]. Subsequently, the potential unrelated word prime candidate was checked to ensure that it was not: (1) related with the target word (i.e., not listed as an associate in NALC, plus a subjective validation performed by the authors); (2) equal or very similar to other previously selected words (either related primes, targets, or other unrelated primes); or (3) orthographically overlapped in its initial letters with the target word (e.g., *polen-potasio* [pollen-potassium]). The search-validation process was repeated as many times as necessary to find a word that was suitable to act as unrelated prime. Independent samples t-tests (JASP, version 0.18.2.0, [58]) (S4.4 in S4 File) indicated that unrelated primes did not significantly differ from related primes in their group means in any word property (all $p \geq .130$, all 95% CI for means difference containing the 0, all $BF_{01} \geq 2.98$, all 95% CrI (S4.5 in S4 File) for effect size containing the 0; see Table 1). Furthermore, word properties' distributions did not significantly differ between related and unrelated pairs, as

indicated by two-sample independent Kolmogorov-Smirnov tests (all $p \geq .327$) (SPSS, version 29.0, [59]). Finally, the related and unrelated primes of each target were matched in grammatical category: the two primes of a given triplet were both verbs (restricted to infinitive forms) or names/adjectives (considered together because it is quite frequent to have words that can behave both as noun and as adjective).

Filler trials. In order to have the same number of 'yes' and 'no' responses in the LDT, it was also necessary to create 200 word-pseudoword pairs (the pairing was performed randomly). These 200 filler trials were the same in both experimental lists.

Prime words for the filler trials were also selected with Match [57]. We selected these primes under the restriction that they should not be too similar in their lexical form to any of the words already selected as the critical items (either related primes, unrelated primes, or targets) or to other filler primes. Independent samples t-tests revealed that this word set did not significantly differ in their group mean in any word property from either the related primes (all $p \geq .281$, all 95% CI for means difference containing the 0, all $BF_{01} \geq 5.15$, all posterior distribution 95% CrI for effect size containing the 0) or the unrelated primes (all $p \geq .417$, all 95% CI for means difference containing the 0, all $BF_{01} \geq 6.57$, all posterior distribution 95% CrI for effect size containing the 0) of the critical trials (see Table 1). Two-sample independent Kolmogorov-Smirnov tests indicated that word properties' distributions for filler primes did not significantly differ from the distributions of either the related (all $p \geq .220$) or the unrelated critical primes (all $p \geq .142$). Furthermore, the proportion of words of different grammatical categories (number of verbs vs. number of nouns/adjectives) was the same for prime words in filler trials as for prime words in critical trials.

Target pseudowords of the filler trials were generated with Wuggy [60] starting from the target words of the critical trials. Pseudowords were matched to critical target words on sub-syllabic structure, length, and transition frequencies. Spanish and Catalan pseudohomophones were avoided, and accents were added to some pseudowords.

Popular Epistemically Unwarranted Beliefs Inventory (PEUBI). PEUBI is a psychometric instrument developed to measure EUB [36]. It consists of 36 items on a 5-point scale (*1 = Fully disagree*, *5 = Fully agree*) loading in five correlated factors: Superstitions (PEUBI-S); Occultism and Pseudoscience (PEUBI-OP); Traditional Religion (PEUBI-TR); Extraordinary Life Forms (PEUBI-ELF); and Conspiracy Theories (PEUBI-CT).

Pseudoscientific Belief Scale, revised version (PSEUDO-R). Given that pseudoscientific beliefs included in PEUBI are somewhat limited (i.e., only related to New Age movement and occultism), PSEUDO-R was included as it is a psychometric instrument developed to measure this specific subgroup of EUB more extensively [61]. It consists of 19 items on a 5-point scale (*1 = Strongly disagree*, *5 = Strongly agree*) loading on a single factor.

Procedure

The study was conducted in sessions in which groups of up to three participants were tested, from 13/April/2023 to 22/May/2023. Participants were first instructed to read and complete the informed written consent form. They were then asked to complete the primed LDT. After they completed the task, they were asked to complete PEUBI and PSEUDO-R questionnaires (in this order). Finally, participants were debriefed if they wanted to.

Primed LDT. The structure of a primed LDT trial can be seen in Fig 1. Each trial started with a fixation cross (+) which was presented in the centre of the screen for 500 ms. Then the fixation cross was replaced by the prime word (Arial font, size 11, lowercase), which was presented on screen for 200 ms. Participants were instructed not to respond to the prime stimulus but just to read it silently. Immediately after the prime offset, the prime word was replaced by the target stimulus (Arial font, size 11, uppercase). Participants

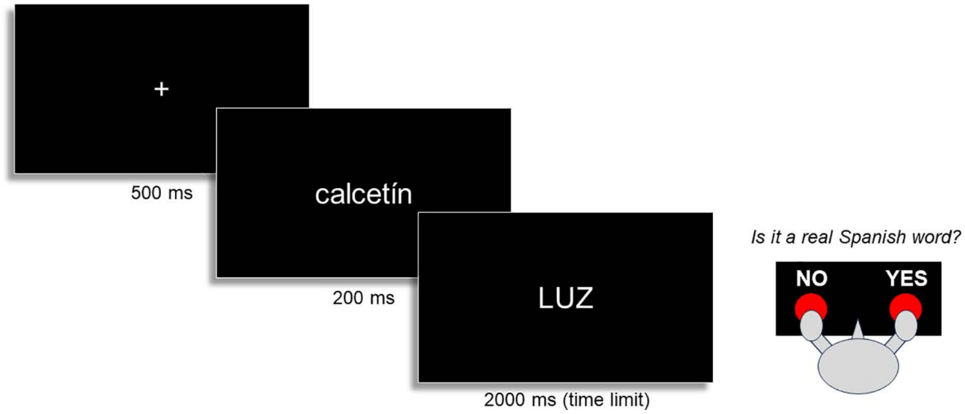

Fig 1. Example of a trial in the primed lexical decision task.

https://doi.org/10.1371/journal.pone.0313239.g001

had to indicate, as quickly and as accurately as possible, if the target stimulus was a real Spanish word ('yes' button pressed with the index finger of the dominant hand) or not ('no' button pressed with the index finger of the other hand). The target remained on screen until the participant produced a response or until the time limit of 2,000 ms was reached. Participants did not receive any feedback either on RT or accuracy. After an intertrial interval of 750 ms the next trial started automatically. Breaks were included every 100 trials. Participants pushed a foot pedal to finish the break and continue the experiment. Before starting the experimental trials, there were 10 practice trials during which the experimenter was present to help them if necessary. We used DMDX software [62] to present stimuli and record responses.

EUB assessment. Both PEUBI and PSEUDO-R were implemented in the computer. Participants only saw one item per screen, and they had to respond by clicking the desired option on the 5-point scale (option-button response format).

Data analysis

EUB scores. The value for each EUB score was obtained by summing its corresponding items (appropriately treating reverse scoring items). For each of these EUB scores, descriptive statistics (i.e., *M*, *SD*, range, skewness) and reliability estimates (McDonald's ω and Cronbach's α, with their corresponding 95% CI) were calculated in JASP. Additionally, pairwise Pearson correlation coefficients and their 95% CI between EUB scores were also calculated in JASP.

Primed LDT. Analyses were performed in RStudio (version 2023.12.0, [63]; R version 4.3.2, [64]) using the following libraries: *bayestestR* (version 0.13.1.2, [65]), *BayesTools* (version 0.2.16, [66]), *brms* (version 2.20.4, [67]), *datawizard* (version 0.9.1, [68]), *emmeans* (version 1.9.0, [69]), *ggeffects* (version 1.3.4, [70]), *ggplot2* (version 3.4.4, [71]), *LMERConvenienceFunctions* (version 3.0, [72]), *performance* (version 0.10.8; [73]), *psych* (version 2.3.12, [74]), *readxl* (version 1.4.3, [75]) *rethinking* (version 2.40, [76]), *splithalf* (version 0.8.2; [77]). (S4.6 in S4 File).

For brevity, many analytical and technical decisions are identified but not described in detail in the present report. A more detailed account of our analytical and technical decisions (along with relevant code) can be found in *S1 File* (see *S2 File* document for a Spanish translation).

Data trimming. The original dataset had 39,600 trial-level RTs (400 items x 99 participants). However, several data trimming criteria were applied to prepare these data for analysis. First, participants with an overall task error rate of >25% were excluded: the 400 observations of one

participant were not included in the analysis under this rule. Second, only data from critical trials were included in the analyses: the 19,600 observations corresponding to filler trials were not analysed. Third, we checked that none of the items had a mean error rate of >70%. No critical item had to be excluded for this reason. Fourth, we removed observations with either display or visualization errors or incorrect responses: 517 observations were excluded for this reason. Fifth, RTs < 300 ms or that reached the 2,000 ms time limit were removed: 37 observations were excluded for this reason. Finally, we excluded, for each participant, RTs observations beyond ±2.5 SD of the participant's mean RT: 610 observations were excluded for this reason. In sum, we conducted our analysis of primed LDT performance using 18,436 observations.

Reliability estimates were obtained for RTs after the data trimming procedure. More concretely, Spearman-Brown corrected split-half reliabilities (and their 95% CI) were estimated with a permutation-based computation in the *splithalf* package [77] for average RTs of the two Relatedness conditions with 5,000 random splits.

Variables adjustment. We retrieved $\log_{10}$-transformed word frequency and contextual diversity measures from the EsPal database [50]. We retrieved raw sublexical frequency measures (bigram frequency and trigram frequency) from EsPal, calculating the $\log_{10}$-transformed values for use in our LMEMs analyses (for a rationale for using log-transformed frequencies, see [78,79 Chapter five]).

Categorical predictors were sum-coded: previous error was coded as *−1 = yes* and *+1 = no*; Relatedness was coded as *−1 = unrelated* and *+1 = related*; list was coded as *−1 = list B* and *+1 = list A*). Continuous interval scale predictors were standardized.

Proxies of the same construct were expected to be collinear: the two variables of lexical frequency; the three variables of lexical neighbourhood; and the two variables of sublexical frequency. Therefore, a preventive removal of variables was performed: only one of each of these variables was finally included in the analyses. We selected a priori the proxy of each construct that subjectively felt most often used in the literature: word frequency as the lexical frequency measure; number of orthographical neighbours as the lexical neighbourhood measure; and bigram frequency as the sublexical frequency measure.

Analysis specifications. Raw RTs were analysed with Bayesian LMEMs using the *brms* package ([67]; for accessible introductions, see [80,81]). Given the typical right/positive skewness of RTs, the Ex-Gaussian function was used as the reference distribution. We specified the somewhat informative prior distribution $\alpha \sim \text{Normal}(700, 200)$ for the intercept, the weakly informative prior $\beta \sim \text{Normal}(0, 50)$ for the slopes of fixed effects, the weakly informative prior $\sigma \sim \text{Normal}_{+}(0, 50)$ for standard deviations of random effects, and the weakly informative prior $\rho \sim \text{LKJ}(2)$ for correlations between random effects. A total of four sampling chains were run in parallel, with 10,000 samples each (including 2,000 for the warm-up phase).

We specified fixed-effects structure based on our theoretical assumptions. Critical predictors were set based on our aims and predictions: Relatedness (we expect to replicate the typical direct priming effect); EUB (a main facilitatory effect is expected if semantic networks of EUB believers generally experience an enhanced spreading of activation); and Relatedness x EUB (if EUB facilitation occurs either only for related prime-target pairs or only for unrelated prime-target pairs). Control predictors were specified based on the identification of potential confounding variables in the literature (see [29,78,82–84]). The maximal random-effects structure was motivated by experimental design [85], with participants and target words as grouping units. Therefore, the model formula was the following:

RTs ~ 1 + prime word Age of acquisition + prime word Concreteness + prime word Familiarity + prime word Valence + prime word Arousal + prime word NLD Spanish-Catalan + prime word Prevalence + prime word Frequency + prime word Length + prime word

Number of orthographic neighbours + prime word Bigram frequency + target word Age of acquisition + target word Concreteness + target word Familiarity + target word Valence + target word Arousal + target word NLD Spanish-Catalan + target word Prevalence + target word Frequency + target word Length + target word Number of orthographic neighbours + target word Bigram frequency + Trial order + Previous RT + Previous error + Relatedness + List + EUB + Relatedness:List + Relatedness:EUB + (1 + Relatedness | Participant) + (1 + Relatedness + EUB | Target)

We performed a separate analysis incorporating each possible EUB score on its own (PEUBI-S, PEUBI-OP, PEUBI-TR, PEUBI-ELF, PEUBI-CT, PSEUDO-R).

Model checks. First, we computed variance inflation factor (VIF) values to check that there were no important degrees of collinearity between predictors (all predictors had VIF ≤ 2.44). Second, posterior predictive checks were performed to ensure that the Ex-Gaussian distribution was appropriate to model our RTs data. Third, some diagnostics were inspected to check whether the MCMC procedure had any convergence or efficiency issue [86 Chapter five, 87]: trace and trank plots (all chains for each estimate had appropriate visual pattern); $\hat{R}$ Gelman-Rubin convergence diagnostic (all estimates had $\hat{R} < 1.01$); and effective sample size (all estimates had EES >100 times the number of chains).

Sample size and sensitivity/power. Starting from the rule of thumb of having a minimum of 30-50 participants and 30-50 items (i.e., 900-2,500 observations; see [88]), the number of observations finally analysed in this study (i.e., 18,436) was deemed to be enough.

Results

EUB scores

Descriptive statistics and reliability estimates for each EUB score can be found in Table 2, and the intercorrelations between EUB scores are presented in Table 3.

Primed LDT

For the sake of brevity, only a qualitative summary of the Bayesian LMEMs results is provided here. For a complete report of these analyses, please see the S3 File.

Reliability. Reliability estimates for RTs were r_{SB} = .98, 95% CI [.97, .99] for the related condition and r_{SB} = .98, 95% CI [.97, .99] for the unrelated condition.

Critical predictors. Posterior distribution estimates for Relatedness were consistently negative (95% CrI). This provides evidence for a main effect of this predictor: we observed

Table 2. Descriptive statistics and reliability estimates of epistemically unwarranted beliefs' scores for the 98 final participants of the primed lexical decision task.

	Descriptive statistics				Reliability estimates	
	M	*SD*	Range (Min–Max)	Skewness	McDonald's ω [95% CI]	Cronbach's α [95% CI]
PEUBI-S	15.78	6.47	7-32	0.50	.89 [.85, .92]	.88 [.84, .91]
PEUBI-OP	29.09	8.98	11-52	0.24	.90 [.86, .93]	.89 [.86, .92]
PEUBI-TR	11.01	5.50	6-27	0.97	.91 [.89, .94]	.91 [.88, .93]
PEUBI-ELF	10.55	3.88	6-23	0.73	.75 [.68, .83]	.75 [.66, .81]
PEUBI-CT	18.58	4.30	7-29	-0.05	.79 [.72, .85]	.77 [.69, .83]
PSEUDO-R	56.56	8.73	38-79	-0.26	.81 [.75, .86]	.79 [.73, .85]

Note. PEUBI-S = superstitions; PEUBI-OP = occultism and pseudoscience; PEUBI-TR = traditional religion; PEUBI-ELF = extraordinary life forms; PEUBI-CT = conspiracy theories; PSEUDO-R = pseudoscience.

https://doi.org/10.1371/journal.pone.0313239.t002

Table 3. Correlation matrix between epistemically unwarranted beliefs' scores for the 98 final participants of the primed lexical decision task.

	PEUBI-S	PEUBI-OP	PEUBI-TR	PEUBI-ELF	PEUBI-CT	PSEUDO-R
PEUBI-S	—					
PEUBI-OP	.57*** [.42, .69]	—				
PEUBI-TR	.32** [.13, .48]	.20 [-.00, .38]	—			
PEUBI-ELF	.55*** [.39, .68]	.68*** [.56, .77]	.30** [.10, .47]	—		
PEUBI-CT	.26** [.07, .44]	.45*** [.27, .59]	.28** [.09, .45]	.39*** [.21, .55]	—	
PSEUDO-R	.46*** [.29, .60]	.64*** [.51, .75]	.26** [.07, .44]	.49*** [.33, .63]	.41*** [.23, .57]	—

Note. Pearson correlation coefficient (95% CI in brackets). PEUBI-S = superstitions; PEUBI-OP = occultism and pseudoscience; PEUBI-TR = traditional religion; PEUBI-ELF = extraordinary life forms; PEUBI-CT = conspiracy theories; PSEUDO-R = pseudoscience.

* $p < .05$, ** $p < .01$, *** $p < .001$.

https://doi.org/10.1371/journal.pone.0313239.t003

faster RTs in the related condition than in the unrelated condition, that is, a direct priming effect. (S4.7 in S4 File).

Posterior distribution estimates for PEUBI-S, PEUBI-OP and PEUBI-TR were consistently negative (95% CrI). This provides evidence for a facilitatory main effect of these predictors: the higher the value for scores on these EUB dimensions, the faster RTs. However, posterior distribution estimates for PEUBI-ELF, PEUBI-CT and PSEUDO-R encompassed the zero (95% CrI). This implies that the data are compatible with null or near-null main effects of these predictors.

Finally, posterior distribution estimates for Relatedness x PSEUDO-R were consistently positive (95% CrI). This provides evidence for the effect of an interaction between these two predictors: the higher the PSEUDO-R score, the lower the direct priming effect (smaller RTs difference between related and unrelated conditions). However, posterior distribution estimates for Relatedness x PEUBI-S, Relatedness x PEUBI-OP, Relatedness x PEUBI-TR, Relatedness x PEUBI-ELF, and Relatedness x PEUBI-CT encompassed the zero (95% CrI). This implies that the data are compatible with null or near-null interactions between Relatedness and the remaining EUB dimensions. Figs 2–7 show the marginal effects (S4.8 in S4 File) of the critical predictors of the present study.

Control predictors. Posterior distribution estimates for some control predictors were consistently negative (95% CrI). This provides evidence for a facilitatory main effect of the following predictors: prime familiarity (i.e., the higher the familiarity of the prime word, the faster RTs); target familiarity (i.e., the higher the familiarity of the target word, the faster RTs); target frequency (i.e., the higher the frequency of the target word, the faster RTs); and trial (i.e., faster RTs as the task progresses).

Posterior distribution estimates for some control predictors were consistently positive (95% CrI). This provides evidence for a inhibitory main effect of the following predictors: prime age of acquisition (i.e., the higher the age of acquisition of the prime word, the slower RTs); prime number of orthographic neighbours (i.e., the higher the number of orthographic neighbours of the prime word, the slower RTs); target age of acquisition (i.e., the higher the age of acquisition of the target word, the slower RTs; with the exception of the model with PEUBI-TR as EUB score, in which the posterior distribution 95% CrI encompassed the zero and, therefore, was compatible with null main effects); target length (i.e., the higher the number of letters of

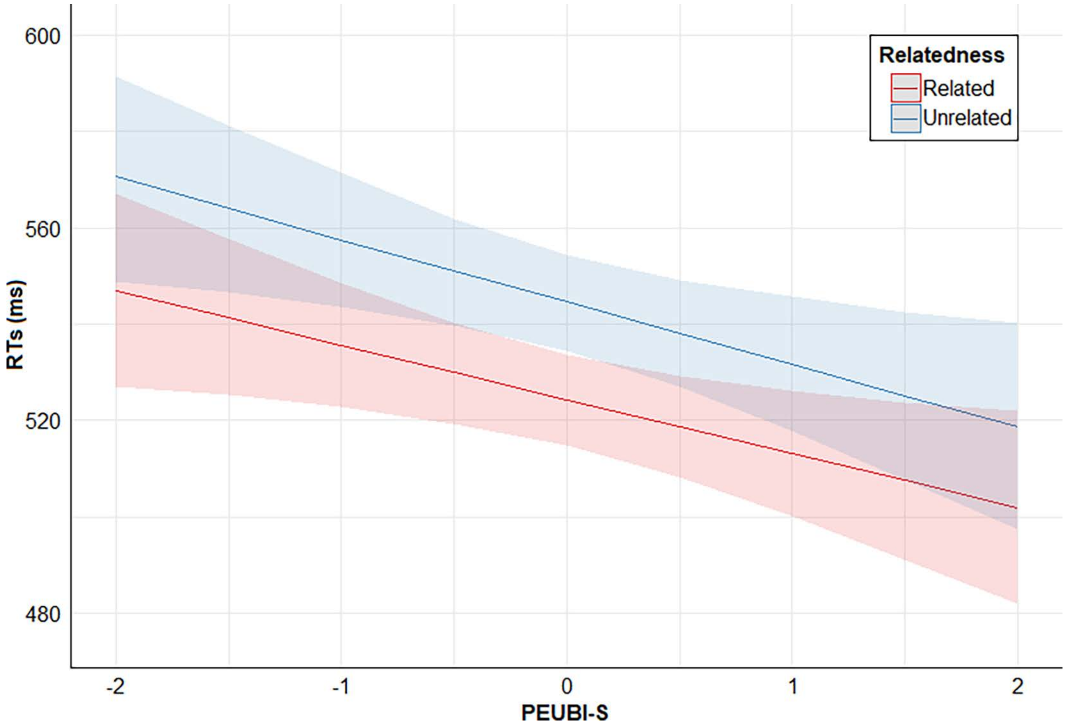

Fig 2. Marginal effects (estimated marginal medians with 95% CrI) for the interaction between Relatedness and PEUBI-S. *Note*. Relatedness = associative/semantic relationship between prime-target words (related [e.g., bulb-light] vs. unrelated [e.g., sock-light]); PEUBI-S = superstitions (epistemically unwarranted beliefs' score).

https://doi.org/10.1371/journal.pone.0313239.g002

the target word, the slower RTs); and previous RT (i.e., the slower the RT of the previous trial, the slower the RT of the current trial).

Posterior distribution estimates for some control predictors encompassed the zero (95% CrI). This implies that the data are compatible with null or near-null main effects for the following predictors: prime concreteness (with the exception of the model with PEUBI-TR as EUB score, in which posterior distribution 95% CrI was consistently positive and, therefore, provided evidence for an inhibitory main effect); prime valence; prime arousal; prime NLD Spanish-Catalan; prime prevalence; prime word frequency; prime length; prime bigram frequency; target concreteness; target valence; target arousal; target NLD Spanish-Catalan; target prevalence; target number of orthographic neighbours; target bigram frequency; previous error; and list.

Posterior distribution estimates for Relatedness x List were consistently negative (95% CrI). This provides evidence for an interactive effect between these two predictors. Though there was a direct priming effect in both lists, this effect was a bit larger in list A than in list B. This seems to be mainly driven by a between-lists difference in unrelated prime-target pairs (i.e., in list A they produced slower RTs than in list B), while the between-lists differences for related prime-target pairs is tiny (S4.9 in S4 File). However, we do not consider the Relatedness x List interaction to be a threat to the validity of our results. First, the pattern of priming effects was similar in both lists and differences were small. Second, this interactive effect is included (and controlled for) in the models. Third, EUB scores were similar between lists (independent samples t-tests: all $p \geq .394$, all 95% CI for means difference containing the 0, all $BF_{01} \geq 3.39$, all posterior distribution 95% CrI for effect size containing the 0; two-samples Kolmogorov-Smirnov tests: all $p \geq .640$).

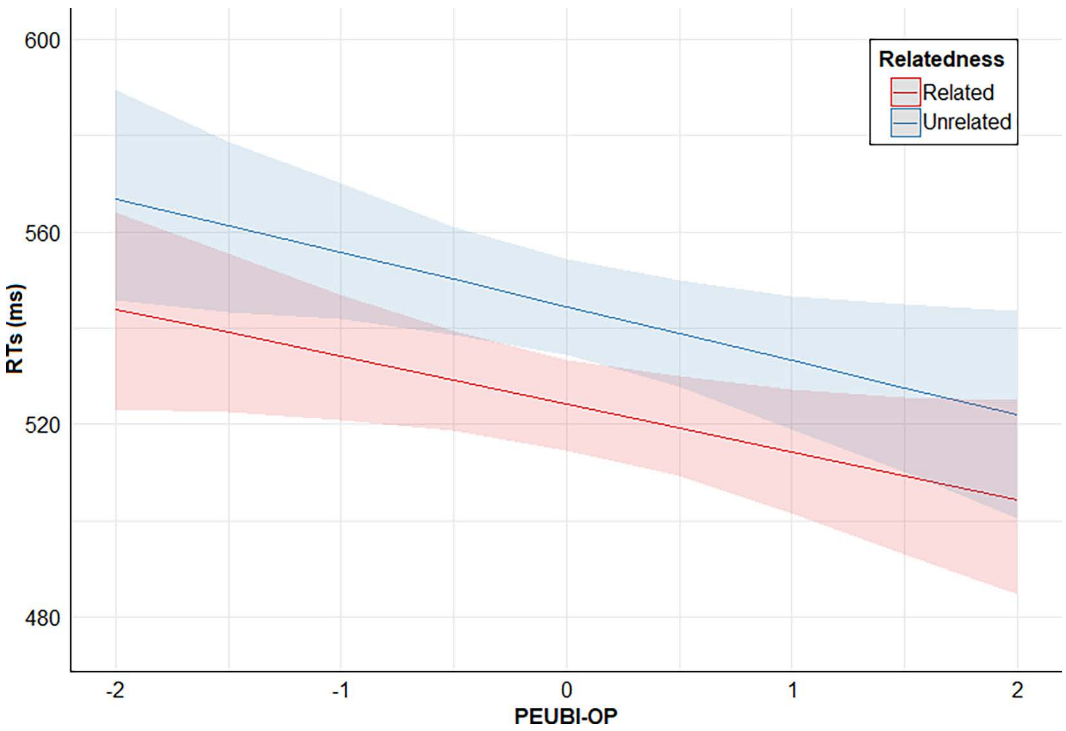

Fig 3. Marginal effects (estimated marginal medians with 95% CrI) for the interaction between Relatedness and PEUBI-OP. *Note*. Relatedness = associative/semantic relationship between prime-target words (related [e.g., bulb-light] vs. unrelated [e.g., sock-light]); PEUBI-OP = occultism and pseudoscience (epistemically unwarranted beliefs' score).

https://doi.org/10.1371/journal.pone.0313239.g003

Discussion and conclusions

Loosening of associations is considered to be as a key underlying disturbance in schizophrenia [5]. One of the explanatory mechanisms that attempts to account for this phenomenon is based on how semantic networks are activated: people diagnosed with schizophrenia would perform illogical or unwarranted associations because of an enhanced spreading of activation through semantic memory (e.g., [6,10–12]), which would lead to co-activation of nodes that are distantly or weakly related. Evidence for this explanatory mechanism partly comes from studies of associative/semantic priming, although there are inconsistencies in the literature [10,12,17]. Grounded in a dimensional approach to psychopathology ([19]; psychosis continuum, [23,38]), the main purpose of the present research was to explore if there are individual differences in associative/semantic priming in people with different levels of EUB. To do so, participants completed a primed LDT containing both related and unrelated prime-target pairs and filled two EUB questionnaires. Bayesian LMEMs over RTs revealed main effects of Relatedness (direct priming effect: faster RTs in the related condition than in the unrelated condition), facilitatory main effects for some EUB scores (i.e., the higher the PEUBI-S, PEUBI-OP or PEUBI-TR scores, the faster RTs) but null main effects for the others (PEUBI-ELF, PEUBI-CT, PSEUDO-R), and an interactive Relatedness x EUB effect for PSEUDO-R only (the higher the PSEUDO-R score, the smaller the priming effect) (S4.10 in S4 File).

As reviewed in the Introduction, we are not aware of previous research to which our results can be directly compared concerning pseudoscientific and conspiracy beliefs, but we can compare our results to those from a small set of prior associative/semantic priming studies concerned with paranormal and magical beliefs [40–42]. Firstly, the direct priming effect

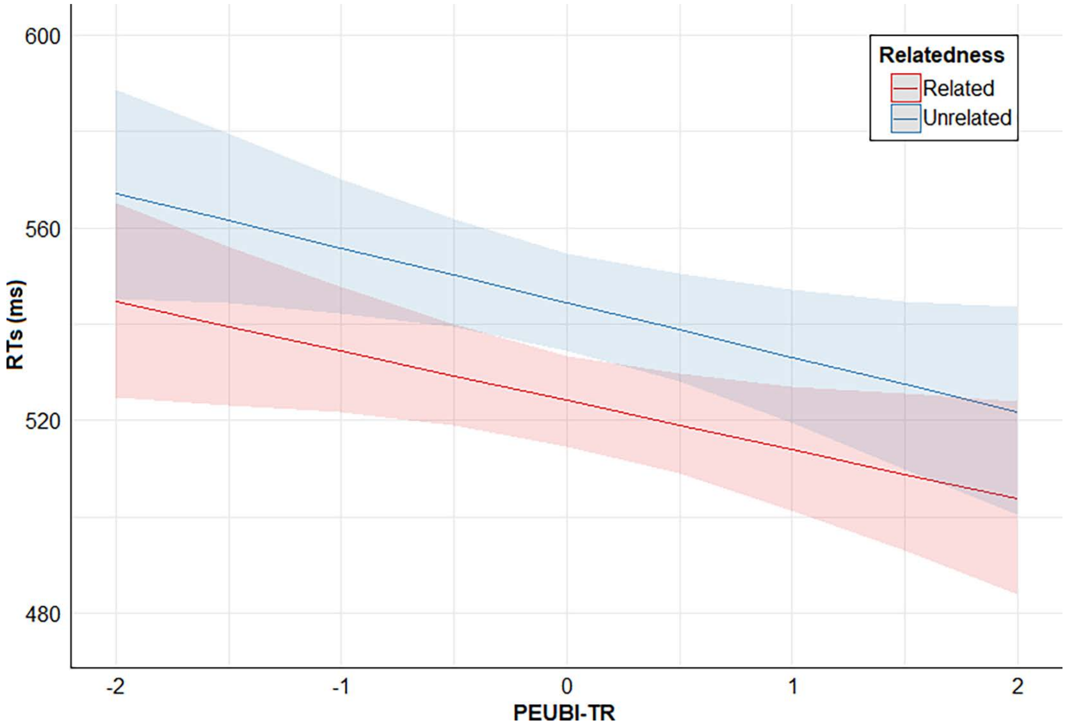

Fig 4. Marginal effects (estimated marginal medians with 95% CrI) for the interaction between Relatedness and PEUBI-TR. *Note*. Relatedness = associative/semantic relationship between prime-target words (related [e.g., bulb-light] vs. unrelated [e.g., sock-light]); PEUBI-TR = traditional religion (epistemically unwarranted beliefs' score).

https://doi.org/10.1371/journal.pone.0313239.g004

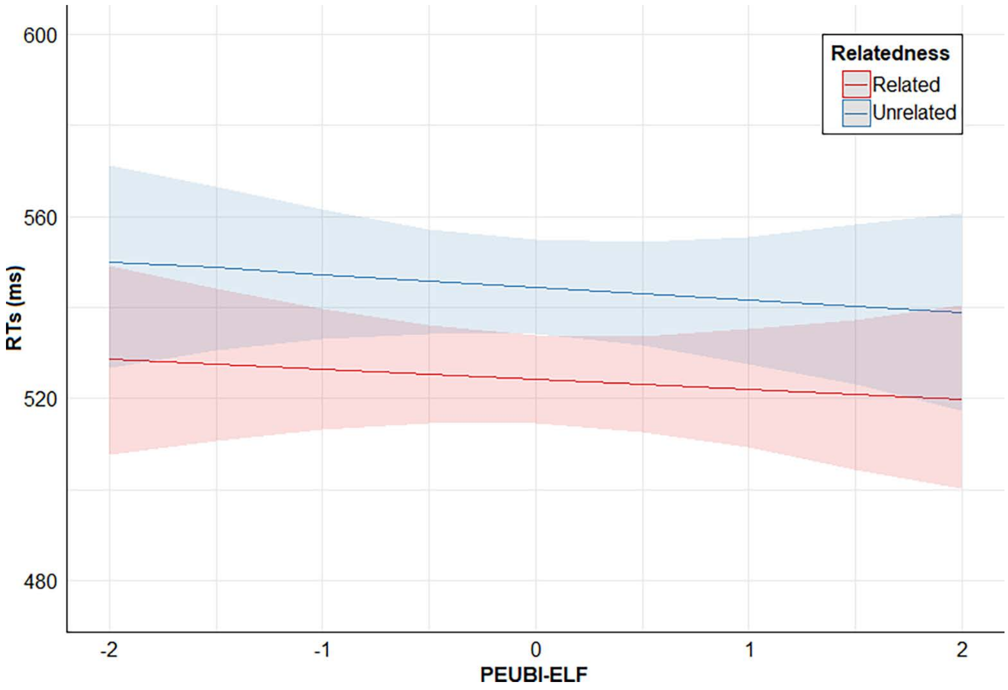

Fig 5. Marginal effects (estimated marginal medians with 95% CrI) for the interaction between Relatedness and PEUBI-ELF. *Note*. Relatedness = associative/semantic relationship between prime-target words (related [e.g., bulb-light] vs. unrelated [e.g., sock-light]); PEUBI-ELF = extraordinary life forms (epistemically unwarranted beliefs' score).

https://doi.org/10.1371/journal.pone.0313239.g005

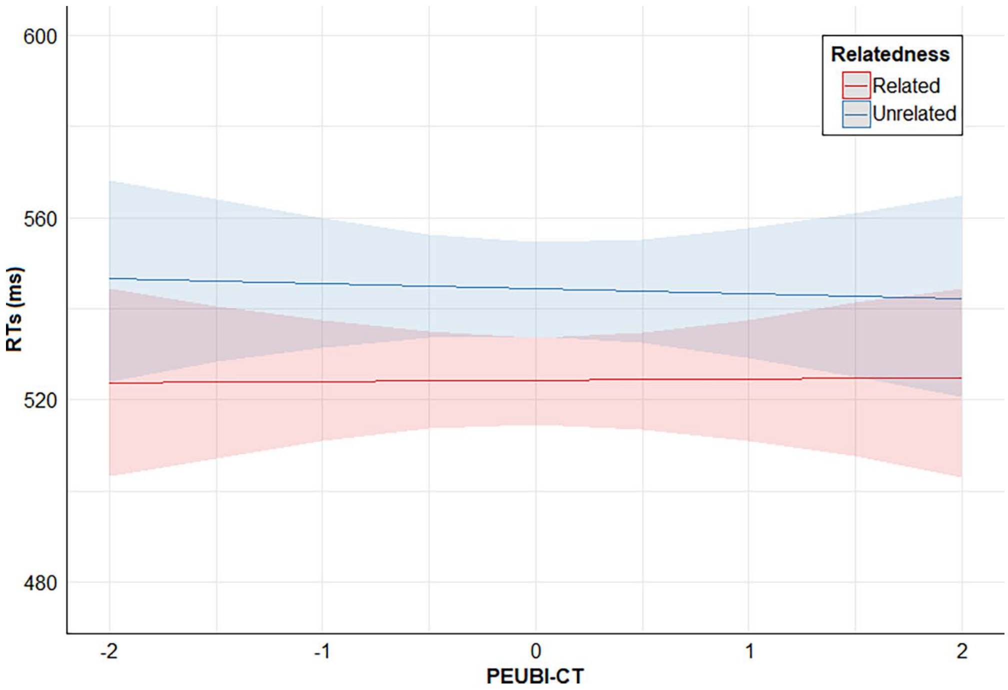

Fig 6. Marginal effects (estimated marginal medians with 95% CrI) for the interaction between Relatedness and PEUBI-CT. *Note*. Relatedness = associative/semantic relationship between prime-target words (related [e.g., bulb-light] vs. unrelated [e.g., sock-light]); PEUBI-CT = conspiracy theories (epistemically unwarranted beliefs' score).

https://doi.org/10.1371/journal.pone.0313239.g006

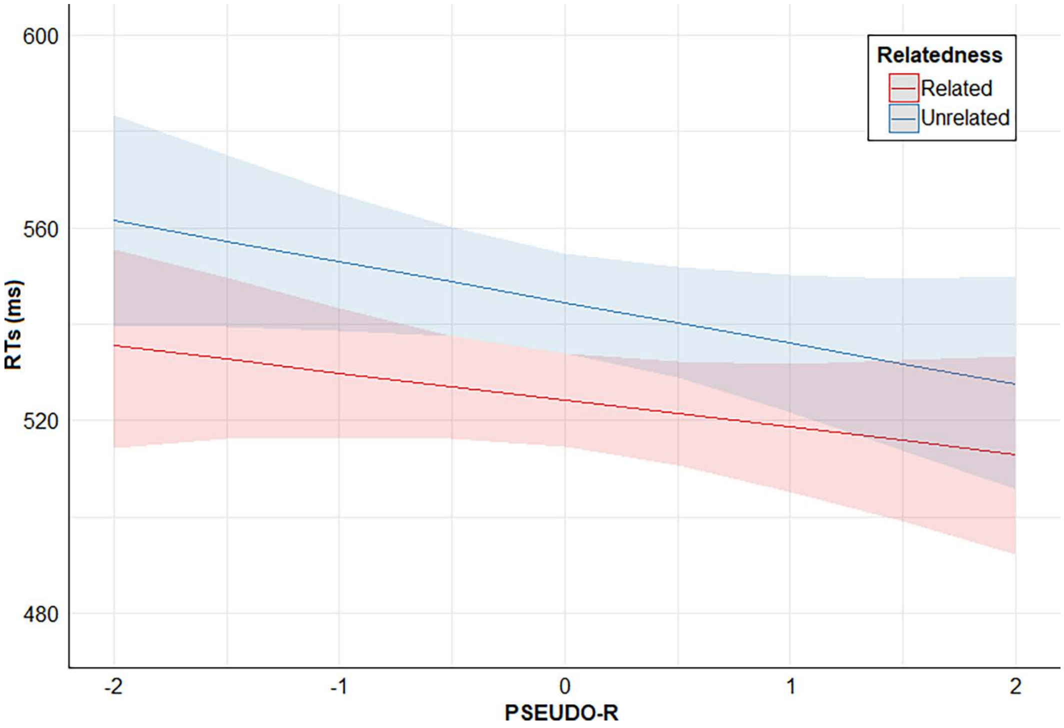

Fig 7. Marginal effects (estimated marginal medians with 95% CrI) for the interaction between prime-target Relatedness and PSEUDO-R. *Note*. Relatedness = associative/semantic relationship between prime-target words (related [e.g., bulb-light] vs. unrelated [e.g., sock-light]); PSEUDO-R = pseudoscience (epistemically unwarranted beliefs' score).

https://doi.org/10.1371/journal.pone.0313239.g007

we found was consistently reported in those previous studies [40–42]. Secondly, facilitatory main effects found for PEUBI-S, PEUBI-OP and PEUBI-TR are consistent with the results of Mohr et al. [42], but not with those of Pizzagalli et al. [41]. A visual inspection of the Fig 1 in Pizzagalli et al. [41] suggests that believers were faster in the three prime-target conditions (i.e., directly related, indirectly related, and unrelated). Perhaps the apparent main effect of the level of paranormal belief was not detected in that study due to lack of statistical power (in their sample there were only 12 believers and 12 disbelievers). Thirdly, the effects of the interaction between Relatedness and PSEUDO-R is not consistent with any of the previous studies [40–42]. While Pizzagalli et al. [41] and Mohr et al. [42] did not observe a modulation of the direct priming effect by the level of paranormal/magical beliefs, Kerns and Berenbaum [40] found this interactive effect but in the opposite direction (i.e., believers showed an increased – not decreased– priming effect in comparison to the control group). Of note, the Relatedness x PSEUDO-R effect in our results seems to be quite small-sized (see Fig 7). Therefore, it should be interpreted with caution and explored in future studies.

Overall, the key finding is that our results are consistent with the predictions made assuming the enhanced spreading of activation mechanism (e.g., [6,10–12]). That is, believers in certain EUB instances may have both a faster/greater spreading activation to close associates (reflected in facilitation in related prime-target pairs) and further reaching spreading activation to remote associates (reflected in facilitation in unrelated prime-target pairs) in comparison to non-believers. One could argue that the facilitatory main effects of some EUB scores could be due to faster general processing/response speed in believers than non-believers. We do not believe this to be the case for the following reasons. Firstly, by-participant random intercepts of LMEMs account/control to some extent for between-participants average/basal differences in RTs. Secondly, facilitatory EUB main effects were not present in response to pseudowords. We conducted Bayesian LMEMs over RTs of the correct responses to pseudowords (filler trials), and the data are compatible with null or near-null main effects for all EUB dimensions (i.e., all 95% CrI encompassed the zero). Thirdly, facilitatory EUB main effects have not been found when the priming paradigm is absent. Huete-Pérez and Ferré [89] performed a study where EUB levels were measured with the same psychometric instruments; the sample of participants was similar to the present one in size, education level, age, sex and EUB distributions; and participants also completed a LDT. However, in that case the LDT was non-primed (i.e., standard lexical decision over words/pseudowords, that is, without being preceded by any prime word) and no main effects for any EUB score was found over LDT RTs. Together, these three pieces of evidence lead us to believe that present EUB facilitatory main effects are specifically due to people with higher EUB levels experiencing an enhanced spreading of activation from both related and unrelated primes to the target (and not to a general faster processing/response speed) in comparison to people with lower levels of EUB. Nevertheless, we cannot fully discard this possibility, just as we cannot rule out the role of other individual differences that can modulate associative/semantic priming (e.g., attentional control and reading ability; [90]) and which may be associated with individual differences in EUB. Future studies should attempt to replicate the main EUB effects found here and, in addition, explore whether they can be accounted for by individual differences in more general cognitive variables.

There is an alternative explanation of the present results more specifically related to semantic memory. Recent findings suggest that differences in associative/semantic priming between people with and without diagnosis of schizophrenia may be due to structural differences in semantic memory [91]. That is, faster RTs to both related and unrelated prime-target pairs may be explained in dynamic terms (i.e., greater speed/strength and reaching of the activation propagation through semantic memory; [6,11]), but could also be explained in structural

terms (i.e., shorter and more 'disorganized' connections in semantic networks; [91]). Future studies could try to disentangle between these two non-exclusive explanatory mechanisms. In any case, given the exploratory nature of the present work together with the few preceding comparable studies, it would be very appropriate for future studies to evaluate whether the present results can be replicated in different participants, items and languages, thus also contributing to assess their generalizability. Going further, a test-retest study could be carried out to assess the temporal stability/reliability of our findings. In this sense, it should be kept in mind the considerations made in the Introduction: varying certain methodological aspects (e.g., short SOA vs. long SOA) may produce in itself different results between studies. Therefore, if the aim is direct replication, future studies should be methodologically as comparable as possible. However, a legitimate aim of future research could be to explore the generalizability of these results to other experimental paradigms and tasks. For instance, although in the present study we minimized the action of controlled and strategic processes by using a short SOA [[15] Chapter nine, [26]], this can be achieved also using a masked priming paradigm (e.g., [92]), in which the conscious exposure of the prime is substantially reduced.

A legitimate question to ask is why the pattern of results varies depending on the specific EUB dimension: we observed facilitatory main effects for some EUB scores but null for others. Although the EUB term is used to designate jointly beliefs that are not logically or empirically grounded [35], and that different EUB instances tend to be related (e.g., [34,36,93]), the multidimensional nature of this construct should not be overlooked (e.g., see [94]). In this regard, the mechanisms underlying different instances of EUB do not necessarily need to be the same (e.g., see [89 Discussions section, 93,94]). Therefore, it could be perfectly plausible that, for instance, high superstitious beliefs (PEUBI-S) but not high conspiracy beliefs (PEUBI-CT) were associated with an enhanced spreading of activation through semantic memory. Going further, unwarranted associations in conspiracy beliefs could be explained in terms of an attitudinal bias of suspiciousness against some individuals, groups and entities (e.g., see [93 Discussion section]), a fact that does not necessarily have to be reflected on semantic memory.

Despite the contributions and strengths of this study, some limitations must be also mentioned. First, the effects of all predictors were explored in a linear fashion, but non-linear relationships could also be possible. Exploring every possible statistical modelling alternative could be an endless process. However, the data are openly available, so that the interested reader can explore how the results would be with different analytical decisions. Second, the sample of participants (undergraduate university students) may not be representative of the general population. As mentioned, future studies could explore whether the present results can be replicated with a different sample of participants.

To conclude, this study suggests that there are individual differences in associative/semantic priming driven by participants' individual differences in EUB. This finding adds to the literature regarding associative/semantic priming effects with respect to the psychosis continuum [10,12,17,40–42], and fits to the predictions made from the enhanced spreading of activation explanatory mechanism (e.g., [6,10–12]).

Supporting information

S1 File. Data analysis details (English). More detailed account of our analytical and technical decisions (along with relevant code).
(PDF)

S2 File. Data analysis details (Spanish). The same document as S1 but in Spanish.
(PDF)

S3 File. LMEMs complete reporting. Complete report of Bayesian LMEMs.
(PDF)

S4 File. Endnotes. Refer to this document for references from S4.1 to S4.10.
(PDF)

Author contributions

Conceptualization: Daniel Huete-Pérez, Robert Davies, Javier Rodríguez-Ferreiro, Pilar Ferré.

Data curation: Daniel Huete-Pérez, Robert Davies.

Formal analysis: Daniel Huete-Pérez, Robert Davies.

Funding acquisition: Daniel Huete-Pérez.

Investigation: Daniel Huete-Pérez.

Methodology: Daniel Huete-Pérez, Robert Davies, Javier Rodríguez-Ferreiro, Pilar Ferré.

Project administration: Daniel Huete-Pérez, Pilar Ferré.

Resources: Daniel Huete-Pérez, Robert Davies, Javier Rodríguez-Ferreiro, Pilar Ferré.

Software: Daniel Huete-Pérez, Robert Davies.

Supervision: Daniel Huete-Pérez, Pilar Ferré.

Validation: Daniel Huete-Pérez, Robert Davies.

Visualization: Daniel Huete-Pérez.

Writing – original draft: Daniel Huete-Pérez, Pilar Ferré.

Writing – review & editing: Daniel Huete-Pérez, Robert Davies, Javier Rodríguez-Ferreiro, Pilar Ferré.

References

1. American Psychiatric Association (APA). Schizophrenia spectrum and other psychotic disorders. In: Diagnostic and statistical manual of mental disorders (5th ed.). American Psychiatric Publishing; 2013. pp. 87–122.
2. Correll CU, Schooler NR. Negative symptoms in schizophrenia: A review and clinical guide for recognition, assessment, and treatment. Neuropsychiatr Dis Treat. 2020 Feb 21;16:519–34. https://doi.org/10.2147/NDT.S225643 PMID: 32110026
3. McCutcheon RA, Reis Marques T, Howes OD. Schizophrenia-An overview. JAMA Psychiatry. 2020 Feb 1;77(2):201–210. https://doi.org/10.1001/jamapsychiatry.2019.3360 PMID: 31664453
4. Bleuler E. Dementia praecox or the group of schizophrenias. International Universities Press; 1950.
5. Nestor PG, Levitt JJ, Ohtani T, Newell DT, Shenton ME, Niznikiewicz M. Loosening of associations in chronic schizophrenia: Intersectionality of verbal learning, negative symptoms, and brain structure. Schizophr Bull Open. 2022 Mar 8;3(1):sgac004. https://doi.org/10.1093/schizbullopen/sgac004 PMID: 35295655
6. Kuperberg GR. Language in schizophrenia part 1: An introduction. Lang Linguist Compass. 2010 Aug;4(8):576–589. https://doi.org/10.1111/j.1749-818X.2010.00216.x PMID: 20936080
7. Baskak B, Ozel ET, Atbasoglu EC, Baskak SC. Peculiar word use as a possible trait marker in schizophrenia. Schizophr Res. 2008 Aug;103(1-3):311–7. https://doi.org/10.1016/j.schres.2008.04.025 PMID: 18538546
8. Johnson DE, Shean GD. Word associations and schizophrenic symptoms. J Psychiatr Res. 1993 Jan-Mar;27(1):69–77. https://doi.org/10.1016/0022-3956(93)90051-3 PMID: 8515391
9. Pomarol-Clotet E, Oh TM, Laws KR, McKenna PJ. Semantic priming in schizophrenia: Systematic review and meta-analysis. Br J Psychiatry. 2008 Feb;192(2):92–7. https://doi.org/10.1192/bjp.bp.106.032102 PMID: 18245021

10. Kiang M. Schizotypy and language: A review. J Neurolinguistics. 2010 May;23(3):193–203. https://doi.org/10.1016/j.jneuroling.2009.03.002

11. Kreher DA, Holcomb PJ, Goff D, Kuperberg GR. Neural evidence for faster and further automatic spreading activation in schizophrenic thought disorder. Schizophr Bull. 2008 May;34(3):473–82. https://doi.org/10.1093/schbul/sbm108 PMID: 17905785

12. Rodríguez-Ferreiro J, Aguilera M, Davies R. Semantic priming and schizotypal personality: Reassessing the link between thought disorder and enhanced spreading of semantic activation. PeerJ. 2020 Jul 30;8:e9511. https://doi.org/10.7717/peerj.9511 PMID: 32821532

13. Collins AM, Loftus EF. A spreading-activation theory of semantic processing. Psychol Rev. 1975;82(6):407–28. https://doi.org/10.1037/0033-295X.82.6.407

14. Kumar AA. Semantic memory: A review of methods, models, and current challenges. Psychon Bull Rev. 2021 Feb;28(1):40–80. https://doi.org/10.3758/s13423-020-01792-x PMID: 32885404

15. McNamara TP. Semantic priming: Perspectives from memory and word recognition. Psychology Press; 2005.

16. Anderson MC. Retrieval. In: Baddeley A, Eysenck MW, Anderson MC, editors. Memory (3rd ed.). Routledge; 2020. pp. 237–74.

17. Almeida VN, Radanovic M. Semantic priming and neurobiology in schizophrenia: A theoretical review. Neuropsychologia. 2021 Dec 10;163:108058. https://doi.org/10.1016/j.neuropsychologia.2021.108058 PMID: 34655651

18. Kiang M, Kutas M. Abnormal typicality of responses on a category fluency task in schizotypy. Psychiatry Res. 2006 Dec 7;145(2-3):119–26. https://doi.org/10.1016/j.psychres.2005.12.010 PMID: 17070931

19. Avasthi A, Sarkar S, Grover S. Approaches to psychiatric nosology: A viewpoint. Indian J Psychiatry. 2014 Jul;56(3):301–4. https://doi.org/10.4103/0019-5545.120560 PMID: 25316945

20. Widiger TA, Mullins-Sweatt S. Mental disorders as discrete clinical conditions: Dimensional versus categorical classification. In: Hersen M, Turner SM, Beidel DC, editors. Adult psychopathology and diagnosis (5th ed.). John Wiley & Sons; 2007. pp. 3–33.

21. Ringwald WR, Forbes MK, Wright AGC. Meta-analysis of structural evidence for the Hierarchical Taxonomy of Psychopathology (HiTOP) model. Psychol Med. 2023 Jan;53(2):533–546. https://doi.org/10.1017/S0033291721001902 PMID: 33988108

22. DeRosse P, Karlsgodt KH. Examining the psychosis continuum. Curr Behav Neurosci Rep. 2015 May 1;2(2):80–9. 10.1007/s40473-015-0040-7 PMID: 26052479

23. van Os J, Linscott RJ, Myin-Germeys I, Delespaul P, Krabbendam L. A systematic review and meta-analysis of the psychosis continuum: Evidence for a psychosis proneness-persistence-impairment model of psychotic disorder. Psychol Med. 2009 Feb;39(2):179–95. https://doi.org/10.1017/S0033291708003814 PMID: 18606047

24. Tonelli HA. How semantic deficits in schizotypy help understand language and thought disorders in schizophrenia: A systematic and integrative review. Trends Psychiatry Psychother. 2014 Jun;36(2):75–88. https://doi.org/10.1590/2237-6089-2013-0053 PMID: 27000707

25. Eysenck MW. Semantic memory and stored knowledge. In: Baddeley A, Eysenck MW, Anderson MC, editors. Memory (3rd ed.). Routledge; 2020. pp. 207–35.

26. Hutchison KA, Balota DA, Neely JH, Cortese MJ, Cohen-Shikora ER, Tse CS, et al. The semantic priming project. Behav Res Methods. 2013 Dec;45(4):1099–114. https://doi.org/10.3758/s13428-012-0304-z PMID: 23344737

27. Kreher DA, Holcomb PJ, Kuperberg GR. An electrophysiological investigation of indirect semantic priming. Psychophysiology. 2006 Nov;43(6):550–63. https://doi.org/10.1111/j.1469-8986.2006.00460.x PMID: 17076811

28. Hutchison KA. Attentional control and the relatedness proportion effect in semantic priming. J Exp Psychol Learn Mem Cogn. 2007 Jul;33(4):645–62. https://doi.org/10.1037/0278-7393.33.4.645 PMID: 17576145

29. Baayen RH, Davidson DJ, Bates DM. Mixed-effects modeling with crossed random effects for subjects and items. J Mem Lang. 2008 Nov;59(4):390–412. https://doi.org/10.1016/j.jml.2007.12.005

30. Brown VA. An introduction to linear mixed-effects modeling in R. Adv Methods Pract Psychol Sci. 2021;4(1):251524592096035. https://doi.org/10.1177/2515245920960351

31. Liben-Nowell D, Strand J, Sharp A, Wexler T, Woods K. The danger of testing by selecting controlled subsets, with applications to spoken-word recognition. J Cogn. 2019 Jan 24;2(1):2. https://doi.org/10.5334/joc.51 PMID: 31517221

32. Jonas KG, Cannon TD, Docherty AR, Dwyer D, Gur RC, Gur RE, et al. Psychosis superspectrum I: Nosology, etiology, and lifespan development. Mol Psychiatry. 2024 Jan 10. https://doi.org/10.1038/s41380-023-02388-2 PMID: 38200290

33. Orsolini L, Pompili S, Volpe U. Schizophrenia: A narrative review of etiopathogenetic, diagnostic and treatment aspects. J Clin Med. 2022 Aug 27;11(17):5040. https://doi.org/10.3390/jcm11175040 PMID: 36078967

34. Lobato E, Mendoza J, Sims V, Chin M. Examining the relationship between conspiracy theories, paranormal beliefs, and pseudoscience acceptance among a university population. Appl Cogn Psychol. 2014 Jun;28(5):617–25. https://doi.org/10.1002/acp.3042

35. Dyer KD, Hall RE. Effect of critical thinking education on epistemically unwarranted beliefs in college students. Res High Educ. 2019;60(3):293–314. https://doi.org/10.1007/s11162-018-9513-3

36. Huete-Pérez D, Morales-Vives F, Gavilán JM, Boada R, Haro J. Popular Epistemically Unwarranted Beliefs Inventory (PEUBI): A psychometric instrument for assessing paranormal, pseudoscientific and conspiracy beliefs. Appl Cogn Psychol. 2022 Oct;36(6):1260–76. https://doi.org/10.1002/acp.4010

37. Escolà-Gascón Á, Marín FX, Rusiñol J, Gallifa J. Pseudoscientific beliefs and psychopathological risks increase after COVID-19 social quarantine. Global Health. 2020 Jul 30;16(1):72. https://doi.org/10.1186/s12992-020-00603-1 PMID: 32731864

38. Galbraith N. Delusions and pathologies of belief: Making sense of conspiracy beliefs via the psychosis continuum. In: Cardella V, Gangemi A, editors. Psychopathology and Philosophy of Mind. Routledge; 2021. pp. 117–44. https://doi.org/10.4324/9781003009856-8

39. Hinterbuchinger B, Litvan Z, Meyer EL, Friedrich F, Kaltenboeck A, Gruber M, et al. Psychotic-like experiences in esoterism: A twilight zone? Schizophr Res. 2018 Mar;193:240–243. https://doi.org/10.1016/j.schres.2017.08.009 PMID: 28826998

40. Kerns JG, Berenbaum H. Aberrant semantic and affective processing in people at risk for psychosis. J Abnorm Psychol. 2000 Nov;109(4):728–32. https://doi.org/10.1037//0021-843x.109.4.728 PMID: 11195997

41. Pizzagalli D, Lehmann D, Brugger P. Lateralized direct and indirect semantic priming effects in subjects with paranormal experiences and beliefs. Psychopathology. 2001 Mar-Apr;34(2):75–80. https://doi.org/10.1159/000049284 PMID: 11244378

42. Mohr C, Landis T, Brugger P. Lateralized semantic priming: modulation by levodopa, semantic distance, and participants' magical beliefs. Neuropsychiatr Dis Treat. 2006 Mar;2(1):71–84. https://doi.org/10.2147/ndt.s12160175 PMID: 19412448

43. Alonso MA, Fernandez A, Díez E. Subjective age-of-acquisition norms for 7,039 Spanish words. Behav Res Methods. 2015 Mar;47(1):268–74. https://doi.org/10.3758/s13428-014-0454-2 PMID: 24578152

44. Ferré P, Haro J, Huete-Pérez D, Fraga I. Emotionality effects in ambiguous word recognition: The crucial role of the affective congruence between distinct meanings of ambiguous words. Q J Exp Psychol (Hove). 2021 Jul;74(7):1234–1243. https://doi.org/10.1177/1747021821990003 PMID: 33438523

45. Hinojosa JA, Rincón-Pérez I, Romero-Ferreiro MV, Martínez-García N, Villalba-García C, Montoro PR, et al. The Madrid Affective Database for Spanish (MADS): Ratings of dominance, familiarity, subjective age of acquisition and sensory experience. PLoS One. 2016 May 26;11(5):e0155866. https://doi.org/10.1371/journal.pone.0155866 PMID: 27227521

46. Ferré P, Guasch M, Moldovan C, Sánchez-Casas R. Affective norms for 380 Spanish words belonging to three different semantic categories. Behav Res Methods. 2012 Jun;44(2):395–403. https://doi.org/10.3758/s13428-011-0165-x PMID: 22042646

47. Guasch M, Ferré P, Fraga I. Spanish norms for affective and lexico-semantic variables for 1,400 words. Behav Res Methods. 2016 Dec;48(4):1358–1369. https://doi.org/10.3758/s13428-015-0684-y PMID: 26542969

48. Hinojosa JA, Martínez-García N, Villalba-García C, Fernández-Folgueiras U, Sánchez-Carmona A, Pozo MA, et al. Affective norms of 875 Spanish words for five discrete emotional categories and two emotional dimensions. Behav Res Methods. 2016 Mar;48(1):272–84. https://doi.org/10.3758/s13428-015-0572-5 PMID: 25740761

49. Stadthagen-Gonzalez H, Imbault C, Pérez Sánchez MA, Brysbaert M. Norms of valence and arousal for 14,031 Spanish words. Behav Res Methods. 2017 Feb;49(1):111–123. https://doi.org/10.3758/s13428-015-0700-2 PMID: 26850056

50. Duchon A, Perea M, Sebastián-Gallés N, Martí A, Carreiras M. EsPal: One-stop shopping for Spanish word properties. Behav Res Methods. 2013 Dec;45(4):1246–58. https://doi.org/10.3758/s13428-013-0326-1 PMID: 23468181

51. Guasch M, Boada R, Ferré P, Sánchez-Casas R. NIM: A Web-based Swiss army knife to select stimuli for psycholinguistic studies. Behav Res Methods. 2013 Sep;45(3):765–71. https://doi.org/10.3758/s13428-012-0296-8 PMID: 23271155

52. Aguasvivas JA, Carreiras M, Brysbaert M, Mandera P, Keuleers E, Duñabeitia JA. SPALEX: A Spanish lexical decision database from a massive online data collection. Front Psychol. 2018 Nov 12;9:2156. https://doi.org/10.3389/fpsyg.2018.02156 PMID: 30483181

53. Fraga I, Guasch M, Haro J, Padrón I, Ferré P. EmoFinder: The meeting point for Spanish emotional words. Behav Res Methods. 2018 Feb;50(1):84–93. https://doi.org/10.3758/s13428-017-1006-3 PMID: 29313247

54. Díez E, Alonso MA, Rodríguez N, Fernández A. Free-association norms for a large set of words in Spanish [poster]. 2018. Forthcoming. https://doi.org/10.13140/RG.2.2.17703.70560

55. Fernández A, Díez E, Alonso MA. Normas de Asociación Libre en Castellano (NALC) de la Universidad de Salamanca [online database]. 2019. Available from: http://campus.usal.es/gimc/nalc

56. Fernandez A, Diez E, Alonso MA, Beato MS. Free-association norms for the Spanish names of the Snodgrass and Vanderwart pictures. Behav Res Methods Instrum Comput. 2004 Aug;36(3):577–83. https://doi.org/10.3758/bf03195604 PMID: 15641446

57. van Casteren M, Davis MH. Match: A program to assist in matching the conditions of factorial experiments. Behav Res Methods. 2007 Nov;39(4):973–8. https://doi.org/10.3758/bf03192992 PMID: 18183914

58. JASP Team. Jeffreys's Amazing Statistics Program (JASP) [software]. Version 0.18.2.0. 2024. Available from: https://jasp-stats.org/

59. IBM Corp. IBM SPSS Statistics for Windows [software]. Version 29. 2022.

60. Keuleers E, Brysbaert M. Wuggy: A multilingual pseudoword generator. Behav Res Methods. 2010 Aug;42(3):627–33. https://doi.org/10.3758/BRM.42.3.627 PMID: 20805584

61. Fasce A, Avendaño D, Adrián-Ventura J. Revised and short versions of the pseudoscientific belief scale. Appl Cogn Psychol. 2021 Feb;35(3):828–32. https://doi.org/10.1002/acp.3811

62. Forster KI, Forster JC. DMDX: A windows display program with millisecond accuracy. Behav Res Methods Instrum Comput. 2003 Feb;35(1):116–24. https://doi.org/10.3758/bf03195503 PMID: 12723786

63. RStudio Team. RStudio: Integrated development for R [software]. Version 2023.12.0. 2023. Available from: http://www.rstudio.com/

64. R Core Team. R: A language and environment for statistical computing [software]. Version 4.3.2. 2023. Available from: https://www.r-project.org/

65. Makowski D, Ben-Shachar MS, Lüdecke D. bayestestR: Describing effects and their uncertainty, existence and significance within the Bayesian framework. J Open Source Softw. 2019;4(40):1541. https://doi.org/10.21105/joss.01541

66. Bartoš F. BayesTools: Tools for Bayesian analyses [software]. Version 0.2.16. 2023. Available from: https://cran.r-project.org/web/packages/BayesTools

67. Bürkner PC. brms: An R package for Bayesian multilevel models using Stan. J Stat Softw. 2017;80(1):1–28. https://doi.org/10.18637/jss.v080.i01

68. Patil I, Makowski D, Ben-Shachar MS, Wiernik BM, Bacher E, Lüdecke D. datawizard: An R package for easy data preparation and statistical transformations. J Open Source Softw. 2022;7(78):4684. https://doi.org/10.21105/joss.04684

69. Lenth RV. emmeans: Estimated marginal means, aka least-squares means [software]. Version 1.9.0. 2023. Available from: https://cran.r-project.org/web/packages/emmeans

70. Lüdecke D. ggeffects: Tidy data frames of marginal effects from regression models. J Open Source Softw. 2018;3(26):772. https://doi.org/10.21105/joss.00772

71. Wickham H. ggplot2: Elegant graphics for data analysis. Springer; 2016.

72. Tremblay A, Ransijn J. LMERConvenienceFunctions: Model selection and post-hoc analysis for (G)LMER models [software]. Version 3.0. 2020. Available from: https://cran.r-project.org/web/packages/LMERConvenienceFunctions

73. Lüdecke D, Ben-Shachar MS, Patil I, Waggoner P, Makowski D. performance: An R package for assessment, comparison and testing of statistical models. J Open Source Softw. 2021;60:3139. https://doi.org/10.21105/joss.03139

74. Revelle W. psych: Procedures for psychological, psychometric, and personality research [software]. Version 2.3.12. 2023. Available from: https://cran.r-project.org/web/packages/psych

75. Wickham H, Bryan J. readxl: Read excel files [software]. Version 1.4.3. 2023. Available from: https://cran.r-project.org/web/packages/readxl

76. McElreath R. rethinking: Statistical rethinking book package [software]. Version 2.40. Available from: https://github.com/rmcelreath/rethinking

77. Parsons S. splithalf: Robust estimates of split half reliability. J Open Source Softw. 2021;6(60):3041. https://doi.org/10.21105/joss.03041

78. Adelman JS. Methodological issues with words. In: Adelman JS, editor. Visual word recognition (vol. 1): Models and methods, orthography and phonology. Psychology Press; 2012. pp. 116–38.

79. Massaro DW, Taylor GA, Venezky RL, Jastrzembski JE, Lucas PA. Letter and word perception: Orthographic structure and visual processing in reading. North-Holland; 1980.

80. Nalborczyk L, Batailler C, Lœvenbruck H, Vilain A, Bürkner PC. An introduction to bayesian multilevel models using brms: A case study of gender effects on vowel variability in standard Indonesian. J Speech Lang Hear Res. 2019 May 21;62(5):1225–1242. https://doi.org/10.1044/2018_JSLHR-S-18-0006 PMID: 31082309

81. Vasishth S, Nicenboim B, Beckman ME, Li F, Kong EJ. Bayesian data analysis in the phonetic sciences: A tutorial introduction. J Phon. 2018 Nov;71:147–161. https://doi.org/10.1016/j.wocn.2018.07.008 PMID: 30197458

82. Brysbaert M, Stevens M, Mandera P, Keuleers E. The impact of word prevalence on lexical decision times: Evidence from the Dutch Lexicon Project 2. J Exp Psychol Hum Percept Perform. 2016 Mar;42(3):441–58. https://doi.org/10.1037/xhp0000159 PMID: 26501839

83. Pexman PM. Meaning-based influences on visual word recognition. In: Adelman JS, editor. Visual word recognition (vol. 2): Meaning and context, individuals and development. Psychology Press; 2012. pp. 24–43.

84. Yap MJ, Balota DA. Visual word recognition. In: Pollatsek A, Treiman R, editors. The Oxford handbook of reading. Oxford University Press; 2015. pp. 26–43.

85. Barr DJ, Levy R, Scheepers C, Tily HJ. Random effects structure for confirmatory hypothesis testing: Keep it maximal. J Mem Lang. 2013 Apr;68(3):10.1016/j.jml.2012.11.001. https://doi.org/10.1016/j.jml.2012.11.001 PMID: 24403724

86. McElreath R. Statistical rethinking: A Bayesian course with examples in R and STAN (2nd ed.). Champan and Hall/CRC Press; 2020. https://doi.org/10.1201/9780429029608

87. Stan Development Team. Runtime warnings and convergence problems. 2022. Available from: https://mc-stan.org/misc/warnings.html

88. Meteyard L, Davies R. Best practice guidance for linear mixed-effects models in psychological science. J Mem Lang. 2020 Jun;112:104092. https://doi.org/10.1016/j.jml.2020.104092

89. Huete-Pérez D, Ferré P. Individual differences in visual word recognition: The role of epistemically unwarranted beliefs on affective processing and signal detection. Lang Cog. 2023;15(2):314–36. https://doi.org/10.1017/langcog.2022.38

90. Yap M. J., Hutchison K. A., & Tan L. C. (2017). Individual differences in semantic priming performance: Insights from the semantic priming project. In Jones M. N. (Ed.), *Big data in cognitive science* (pp. 203–226). Routledge.

91. Matsumoto Y, Nishida S, Hayashi R, Son S, Murakami A, Yoshikawa N, et al. Disorganization of semantic brain networks in schizophrenia revealed by fMRI. Schizophr Bull. 2023 Mar 15;49(2):498–506. https://doi.org/10.1093/schbul/sbac157 PMID: 36542452

92. Altarriba J., & Basnight-Brown D. M. (2007). Methodological considerations in performing semantic-and translation-priming experiments across languages. *Behavior Research Methods*, 39(1), 1–18. https://doi.org/10.3758/bf03192839 PMID: 17552467

93. Bensley DA, Lilienfeld SO, Rowan KA, Masciocchi CM, Grain F. The generality of belief in unsubstantiated claims. Appl Cogn Psychol. 2020;34(1):16–28. https://doi.org/10.1002/acp.3581

94. Rizeq J, Flora DB, Toplak ME. An examination of the underlying dimensional structure of three domains of contaminated mindware: Paranormal beliefs, conspiracy beliefs, and anti-science attitudes. Think Reasoning. 2020;27(2):187–211. https://doi.org/10.1080/13546783.2020.1759688
